# Warming-driven rise in soil moisture entropy signals growing instability risk in the Asian Water Tower

**DOI:** 10.1038/s41467-026-76955-w

**Published:** 2026-08-21

**Authors:** Yiran Xie, Teng Liu, Xuan Ma, Yingshuo Lyu, Xu Wang, Yatong Qian, Yongwen Zhang, Ming Wang, Xiaosong Chen

**Affiliations:** 1 School of National Safety and Emergency Management, Beijing Normal University, Beijing, China; 2 School of Systems Science and Institute of Nonequilibrium Systems, Beijing Normal University, Beijing, China; 3 Munich Climate Center and Earth System Modelling Group, Department of Aerospace and Geodesy, TUM School of Engineering and Design, Technical University of Munich, Munich, Germany; 4 Potsdam Institute for Climate Impact Research, Potsdam, Germany; 5 State Key Laboratory of Disaster Weather Science and Technology, Chinese Academy of Meteorological Sciences, Beijing, China; 6 Department of Earth System Science, Ministry of Education Key Laboratory for Earth System Modeling, Institute for Global Change Studies, Tsinghua University, Beijing, China; 7 Yunnan Key Laboratory of Complex Systems and Brain-Inspired Intelligence, Kunming University of Science and Technology, Kunming, China; 8 Faculty of Science, Kunming University of Science and Technology, Kunming, China; 9 Joint International Research Laboratory of Catastrophe Simulation and Systemic Risk Governance, Beijing Normal University, Zhuhai, China; 10 Institute for Advanced Study in Physics and School of Physics, Zhejiang University, Hangzhou, China

**Keywords:** Hydrology, Hydrology, Climate change, Nonlinear phenomena

## Abstract

The Tibetan Plateau (TP), known as the Asian Water Tower, supplies water to billions of people but is undergoing rapid warming and wetting. Whether this increased moisture stabilizes the regional hydrological system or merely provides a transient buffer remains unclear. Here we apply an entropy-based framework to quantify the structural organization of the TP’s soil moisture system. During 2000–2024, regional wetting has driven a long-term decline in entropy, reflecting an increase in system order and enhanced hydrological buffering capacity. This ordering is modulated by ENSO, which regulates regional heterogeneity via a spatial dipole. However, CMIP6 projections reveal a reversal: entropy increases under continued warming and regional contrasts intensify, with some models exhibiting transition-like behavior. Our findings suggest that while current wetting provides a stabilizing buffer, continued warming is projected to amplify spatial heterogeneity, thereby elevating instability risk in the Asian Water Tower and threatening downstream water security.

## Abstract (web summary)

This study finds that recent wetting has increased the organization of the Tibetan Plateau’s soil moisture system, enhancing the resilience of the Asian Water Tower, but ENSO-driven variability and projected warming are expected to amplify regional contrasts and instability risk.

## Introduction

Serving as the Asian Water Tower, the Tibetan Plateau (TP) is a central regulator of the Asian and global hydrological cycle, storing and redistributing vast quantities of freshwater through its glaciers, snowpack, lakes, permafrost and river networks^1–4^. As the world’s highest and most expansive plateau, it shapes atmospheric circulations, monsoon dynamics, and ecological stability across Asia and beyond, sustaining the water supply of nearly two billion people downstream^5^. In recent decades, the TP has warmed at roughly twice the global average rate^6^, triggering widespread glacial retreat, permafrost degradation, lake expansion, and shifts in snowmelt timing^3,4,7^, increasingly threatening regional water availability and downstream water security^1,2,7–9^. These changes have raised a concern that extends beyond gradual trends: the TP may be behaving as a climate tipping element, an Earth-system component prone to abrupt and potentially irreversible transitions under small perturbations^10,11^, and approaching a critical threshold. The consequences of such a transition would not remain local: emerging teleconnections link the TP to other tipping elements such as the Amazon rainforest, so its destabilization could trigger cascading effects across the broader Earth system and amplify global climate risk^12–14^. Evaluating this potential tipping behavior is therefore critical, yet existing assessments remain limited. Current early-warning analyses of the TP, which have identified such destabilization within individual subsystems such as snow cover and vegetation^12,15–18^, assess stability largely on a local, location-by-location basis and reveal little about how the system is organized in space, leaving unresolved whether the Plateau’s hydrological system as a whole is gaining or losing stability.

Soil moisture (SM) acts as a critical state variable of the TP’s hydrological system, regulating surface runoff^19,20^, groundwater recharge^21^, vegetation dynamics^22^ and carbon fluxes^23^, thereby underpinning ecosystem resilience and regional water security. Through its influence on evapotranspiration (ET) and the partitioning of surface heat fluxes, SM also governs land–atmosphere exchanges of water and energy, and influences both regional and remote climate variability and feedbacks^23,24^. Specifically, SM anomalies over the TP modulate regional thermal forcing through land–atmosphere interactions, contributing to heat extremes that range from high-altitude heatwaves on the Plateau itself to teleconnected heat events over Europe and East Asia via Rossby wave propagation^25–27^. They also exert hydrological influences beyond the Plateau: wetter spring soil conditions favor an earlier onset of the South Asian summer monsoon and provide predictive information for summer precipitation and flood risk in the Yangtze River basin^28,29^. These widespread impacts imply that changes in the variability and spatial organization of the TP soil moisture system could have far-reaching consequences for both the Plateau and downstream regions. Rather than acting as an isolated climatic driver, SM over the TP serves as an integrating state, accumulating the combined influence of precipitation, temperature, evapotranspiration, and cryospheric processes such as snowmelt and freeze–thaw cycles^27,30,31^. Its relatively long memory allows perturbations to persist rather than dissipate instantaneously, reflecting the net hydrological state of the land surface^32^. Unlike precipitation or temperature, which capture only partial aspects of the water and energy balance, SM records the system’s integrated response. Particularly in the highly heterogeneous and sensitive environment of the TP, this makes SM the natural variable for diagnosing the spatial organization and stability of the TP’s hydrological system.

Accurately characterizing the complex spatiotemporal dynamics of SM across the TP remains challenging. Harsh terrain limits ground-based observations, necessitating reliance on satellite products and reanalysis datasets whose performance varies markedly across the Plateau, complicating their interpretation^33–35^. While recent assessments report an overall wetting trend linked to rising precipitation (Pp) and warming-induced alterations in freeze–thaw cycles, pronounced regional differences persist, and the mechanisms driving these spatial contrasts are not well resolved^30,31,35,36^. Such spatial structure is likely modulated, at least in part, by large-scale climate variability. The El Niño–Southern Oscillation (ENSO), for example, exerts a strong influence on the TP hydroclimate through large-scale atmospheric teleconnections that alter regional circulation and moisture transport^37^. It has been linked to substantial variations in precipitation and snow conditions across the TP, including winter snowfall over the western TP^38^, summer rainfall over its southwestern sector^39^, and snowpack accumulation that may persist into subsequent seasons^40^. These hydroclimatic perturbations can propagate into terrestrial water storage, reflected by pronounced lake-level shrinkage during major El Niño events, followed by widespread lake expansion in subsequent years^41,42^. Despite these documented impacts on multiple components of the TP water cycle, whether and how ENSO shapes the variability and large-scale organization of the TP soil moisture system remains poorly understood.

Linking these spatial structures to system stability, however, demands a perspective that prevailing resilience diagnostics do not provide. To date, stability loss in such systems has been detected largely through statistical early-warning indicators grounded in critical slowing down (CSD) theory, principally rising variance and lag-1 autocorrelation, or a declining recovery rate, which signal a decreasing capacity to recover from perturbations. Applied to the TP, these indicators reveal a persistent loss of stability in snow-cover extent since around 2008^12^ and a parallel decline in vegetation resilience^15^. These approaches, however, share key limitations. CSD-based diagnostics rest on idealized assumptions, including local linearity and quasi-stationarity, that are frequently violated in practice^11^, and they remain sensitive to data quality issues that can bias the inferred resilience^43,44^. More fundamentally, they characterize the temporal dynamics of individual locations, but not the spatial organization of a key state variable across the system, the property that governs the systemic stability of the TP as a whole. This gap is particularly acute for SM, whose dynamics arise from nonlinear interactions among Pp, ET, snowmelt and vegetation feedbacks, and are additionally influenced by stochastic climate variability^45^. Under sustained warming, these coupled processes may trigger amplifying mechanisms, such as plant-soil moisture positive feedbacks^46^, potentially pushing the system toward alternative regimes when stability decreases. Capturing such large-scale behavior requires a metric that goes beyond mean trends to quantify organization, heterogeneity, and resilience. Entropy provides precisely such a diagnostic lens. By quantifying the degree of disorder, entropy reveals whether variability is concentrated in a few dominant modes (order) or dispersed across many (disorder), offering a physically interpretable measure of systemic structure and a potential indicator of heightened risk of abrupt transitions^47–49^. Here, we leverage recent advances in statistical physics to estimate entropy directly from observational and model data using the eigen microstate theory^50–55^. This method extracts dominant system states to characterize their probability distribution^56^, offering a tractable framework for tracking SM disorder under both observed variability and projected climate warming.

**Fig. 1:**
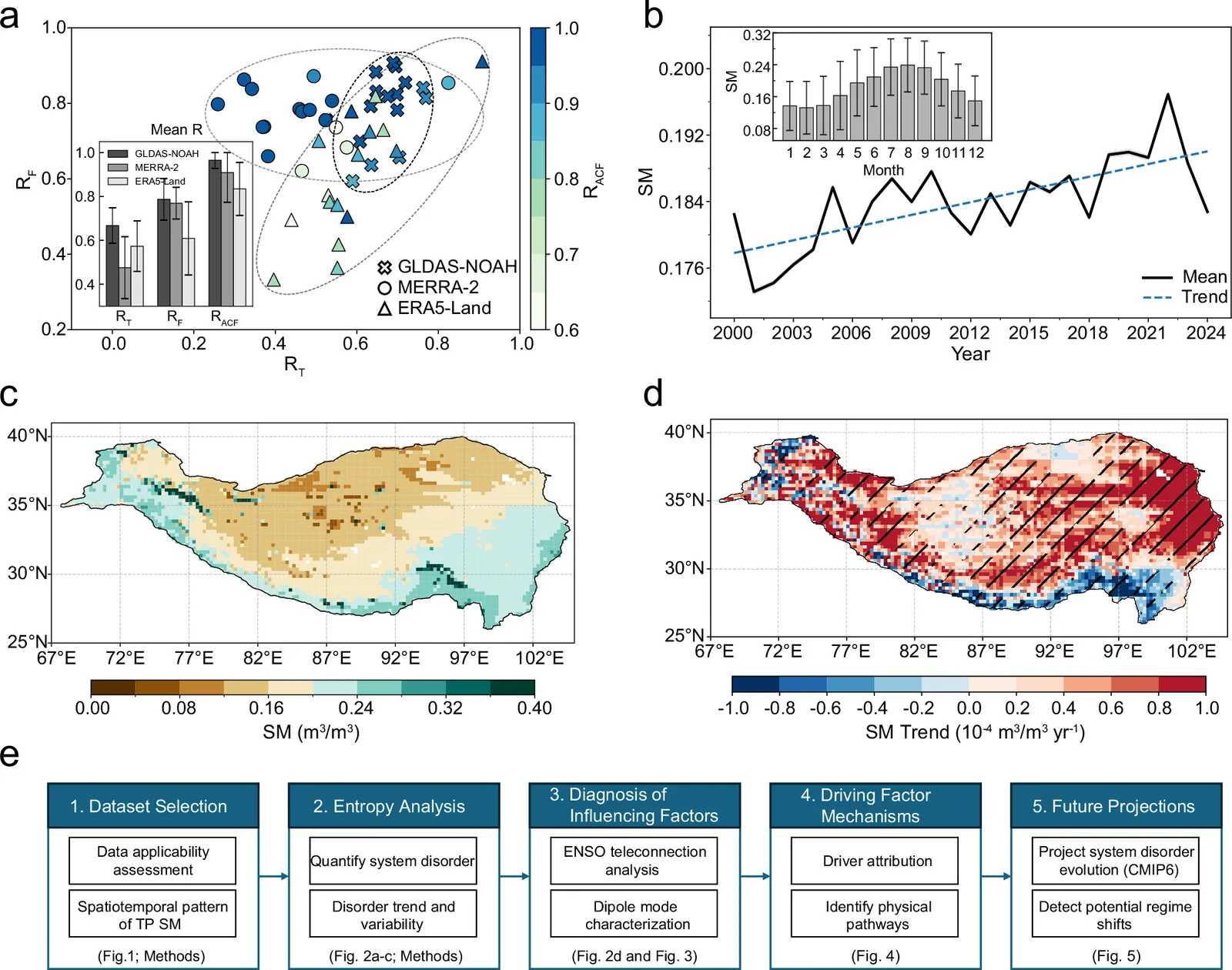
Dataset evaluation, spatiotemporal characteristics, and analytical framework of SM over the TP. **a** Temporal and spectral correlations (*R*_*T*_ and *R*_*F*_), with colors indicating the correlation between autocorrelation sequences (*R*_*ACF*_), computed between in-situ observations and corresponding SM product estimates. The inset bar chart summarizes the three evaluation metrics, with error bars indicating the standard deviation. **b** Temporal evolution of annual mean SM from 2000 to 2024, with shading indicating the standard error. The blue dashed line represents a significant upward trend (*p* < 0.001). The inset bar chart shows the climatological monthly mean SM, with error bars denoting the standard deviation. **c** Spatial distribution of mean SM. **d** Spatial patterns of SM trends from 2000 to 2024. Regions with *p* < 0.05 are marked with diagonal lines. **e** Conceptual schematic of the research workflow.

In this work, we apply this entropy-based diagnostic framework to quantify the spatiotemporal disorder and systemic stability of the SM over the TP. Specifically, we first identify the most suitable SM dataset for TP-wide analysis through a comprehensive evaluation of GLDAS-NOAH, ERA5-Land, and MERRA-2 against in-situ observations, considering both value agreement and dynamical consistency. Using the selected GLDAS-NOAH dataset, we employ soil moisture entropy to characterize the long-term evolution and interannual variability of system disorder across the TP during 2000–2024, and examine its relationship with ENSO. Finally, we use the Coupled Model Intercomparison Project Phase 6 (CMIP6) simulations to evaluate the future evolution of soil moisture system disorder under continued warming, thereby assessing whether the recent gain in stability is likely to persist. The overall framework of this study is illustrated in Fig. 1e.

## Results

### Significant wetting over the Tibetan Plateau during 2000–2024

Accurately characterizing SM variability over the TP remains a major challenge, primarily due to the scarcity of in-situ observations and the region’s complex hydroclimatic conditions. Although several SM datasets, including land surface model outputs (GLDAS-NOAH^57^) and reanalysis datasets (MERRA-2^58^, ERA5-Land^59^), are widely employed in global hydrological and climate studies, each exhibits considerable uncertainties over the TP. These discrepancies stem from differences in model structures, parameterizations, and data assimilation strategies^60^. For instance, MERRA-2 emphasizes atmospheric consistency but often underrepresents land surface heterogeneity^58^; ERA5-Land provides high-resolution estimates yet exhibits a wet bias in SM climatology in semi-humid regions^61^; and GLDAS-NOAH captures SM variability reasonably well but is influenced by uncertainties in the meteorological inputs used to drive the model^60^. Prior studies have highlighted that SM simulations over the TP are particularly sensitive to topography, land cover, and the Pp regime, with model performance exhibiting substantial spatial and temporal variability^30,34^.

Considering the inconsistencies across current SM datasets, we first identify the dataset most suitable for our analysis of the spatiotemporal dynamics of SM across the TP. We develop a robust evaluation framework that jointly considers temporal correlation, spectral characteristics, and autocorrelation structure, using in-situ observations at 5 cm depth from the Tibet-Obs network, which comprises Maqu, Naqu, Ali, and Shiquanhe subregions (Supplementary Fig. 1). The evaluation incorporates three complementary metrics designed to assess different aspects of temporal fidelity. (1) Temporal correlation (*R*_*T*_; see Methods), which quantifies the degree of synchronicity between the evaluated products and site-level measurements. A high *R*_*T*_ indicates strong agreement in the timing and fluctuation patterns of SM variability. (2) Spectral correlation (*R*_*F*_), calculated from the Fourier transforms of evaluated products and observational time series. *R*_*F*_ assesses the preservation of frequency-dependent variability, ensuring the dataset reproduces not only first-order trends but also higher-order dynamical structures embedded in the observed record. (3) Autocorrelation sequence similarity (*R*_*ACF*_), based on lagged autocorrelation coefficients at lags 1–10 (AC1–AC10). *R*_*ACF*_ captures the persistence and memory structure of SM dynamics, with higher similarity indicating a better capability of the evaluated product to replicate the temporal continuity and internal variability of real-world processes.

We find that GLDAS-NOAH consistently outperforms the other datasets across all evaluation criteria. Specifically, it exhibits the highest *R*_*T*_ with in-situ observations and the lowest variability, followed by ERA5-Land, while MERRA-2 performs the worst (Fig. 1a). This superior performance is broadly consistent across the four examined subregions, demonstrating its stable temporal fidelity at finer spatial scales (Supplementary Fig. 2). However, all three products show their largest discrepancies at the Naqu subregion. These deviations may reflect the high-altitude cold alpine environment with pronounced seasonal freeze–thaw cycles, together with the short and discontinuous in-situ records available there^62,63^. Figure 1a also shows that GLDAS-NOAH outperforms the others in both mean *R*_*F*_ and *R*_*ACF*_, indicating its strong ability to capture not only first-order variability but also higher-order dynamical and persistence features of the SM time series. Notably, MERRA-2 outperforms ERA5-Land in *R*_*F*_ and *R*_*ACF*_, in contrast to the pattern observed for mean *R*_*T*_, demonstrating that assessment of dynamical and persistence characteristics yields complementary information beyond conventional first-order statistics. Importantly, the scatterplots in Fig. 1a show that GLDAS-NOAH displays a more compact distribution across all three metrics, as reflected in the tighter clustering of data points compared to ERA5-Land and MERRA-2. This compact performance distribution underscores the product’s spatial robustness across heterogeneous environments. Although GLDAS-NOAH exhibits a moderate overestimation in certain subregions, such as Ali and Shiquanhe, as indicated by relatively high RMSE and bias values, this issue is less critical when the focus is on relative variability rather than absolute values. In fact, it maintains strong performance in *R*_*F*_ and *R*_*ACF*_ across all four subregions, with particularly high consistency in Ali and Shiquanhe (Supplementary Fig. 3). Moreover, at the global scale, GLDAS-NOAH has been shown to capture complex variability exhibiting lower randomness^64^. Based on this comprehensive assessment, we select GLDAS-NOAH as the primary dataset for analyzing the spatiotemporal evolution of SM across the TP.

We then analyzed the spatiotemporal variability of SM across the TP from 2000 to 2024 using the GLDAS-NOAH dataset. Spatially, SM exhibits a pronounced southeast-to-northwest decreasing gradient overall, broadly aligning with the regional Pp pattern (Fig. 1c). This gradient reflects distinct hydroclimatic regimes across the TP: certain high-altitude regions in the southern and northwestern Plateau (e.g., the Himalayas and Hindu Kush) maintain relatively high SM levels due to seasonal snowmelt, whereas most of the arid central and northern Plateau (e.g., the Qaidam Basin and Tanggula Mountains) exhibit markedly lower SM levels. Seasonally, SM follows a distinct annual cycle, increasing from spring to summer and decreasing through autumn and winter. Monthly mean values range from 0.132 m^3^/m^3^ in February to 0.239 m^3^/m^3^ in August, with an average of 0.184 m^3^/m^3^ over the study period (Fig. 1b). However, this seasonal pattern varies regionally: SM peaks in spring over the Pamir Plateau and Hindu Kush Mountains, whereas in the Himalayas, it remains relatively stable throughout the year (Supplementary Fig. 4). Interannually, SM over the TP exhibits a significant increasing trend, with a linear rate of +0.0005 m^3^/m^3^ per year during 2000–2024 (Fig. 1b). The long-term wetting, statistically significant in all seasons except summer (Supplementary Fig. 4), reflects an intensification of the regional hydrological cycle under a warming climate, primarily driven by enhanced Pp. In summer, however, strong ET associated with high temperatures likely offsets the additional moisture input^35,65^. Nevertheless, substantial regional differences persist, with significant wetting covering approximately 57.5% of the TP, whereas significant drying is limited to roughly 8.1%, mainly in the southern Himalayas and parts of the Hengduan Mountains (Fig. 1d).

### ENSO-driven modulation of soil moisture entropy on the Tibetan Plateau

A broadly increasing trend in SM across the TP is reshaping regional hydrological dynamics by enhancing soil water storage, altering the partitioning of ET, and modifying runoff generation. These changes affect both the timing and magnitude of water availability across the Plateau, with important implications for the stability of the regional water cycle. Here, we quantify system disorder using eigen microstate entropy, which captures underlying shifts in structural organization. This metric quantifies how SM variability is distributed across dominant spatial-temporal modes: lower entropy indicates a state that is governed by a few coherent modes, whereas higher entropy reflects a more disordered state lacking clear organization (see Methods). Applying this framework to the TP’s SM reveals a distinct long-term reorganization and clear teleconnections with ENSO events.

**Fig. 2:**
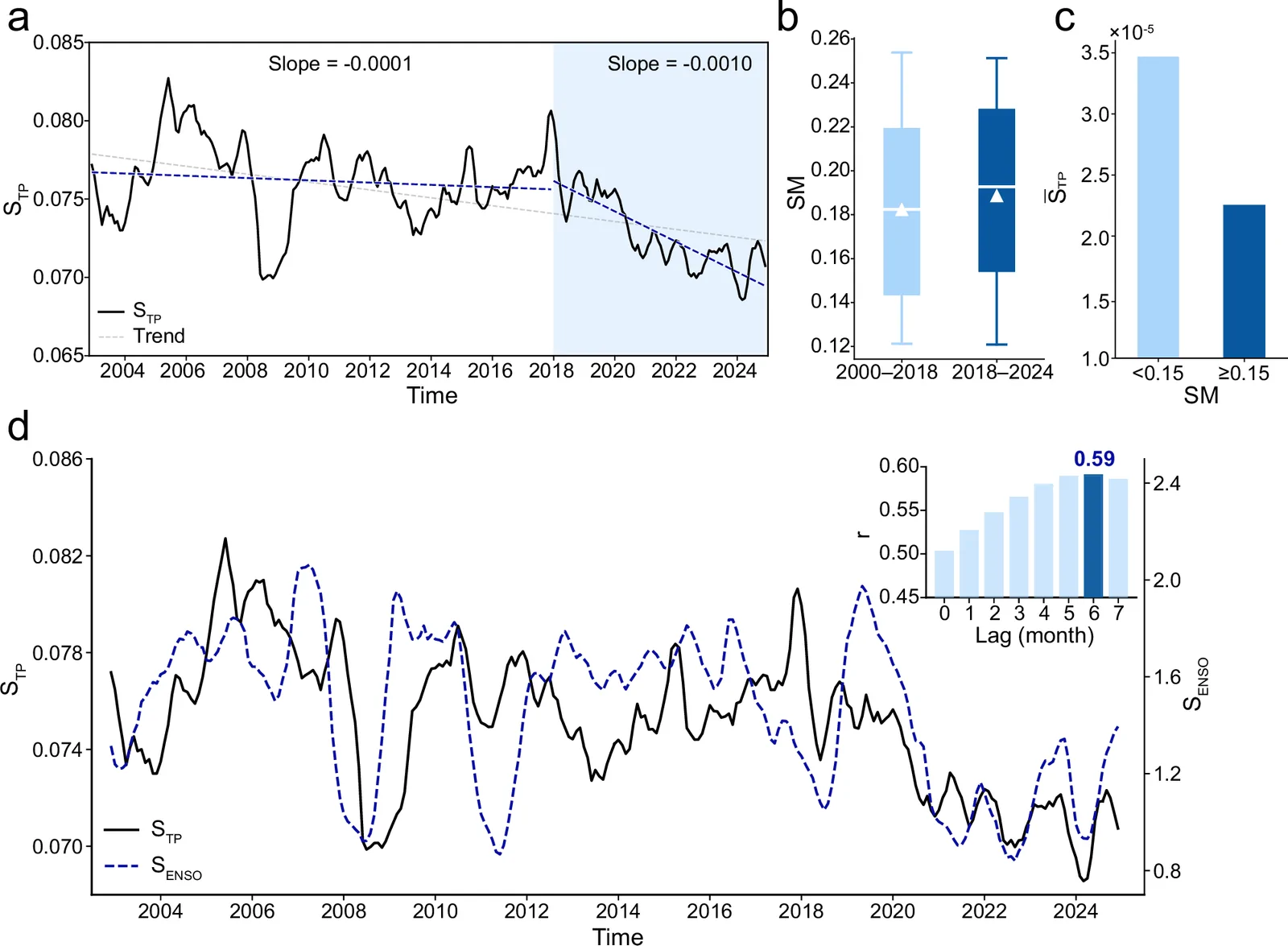
Evolution of *S*_TP_ and its modulation by ENSO. **a** Temporal evolution of *S*_TP_ during 2000–2024 (black), showing a significant decline. A change in the rate of decrease is suggested around 2018 by segmented regression: the trend is weak prior to 2018 (*p* < 0.1) but accelerates afterwards (*p* < 0.001). **b** Boxplots of spatially averaged SM for periods before (January 2000–December 2017) and after 2018 (January 2018–December 2024). The central line represents the median, the box edges indicate the 25th and 75th percentiles, whiskers extend to the most extreme data points within 1.5 times the interquartile range, and triangles denote the mean. Post-2018 periods show consistently higher SM, indicating a shift toward a wetter regime and reduced system disorder (lower entropy). **c** Spatial dependence of entropy on soil moisture. Grid points with higher time-mean SM (≥ 0.15 m^3^ m^−3^) consistently exhibit lower mean entropy (${\bar{S}}_{{{{\rm{TP}}}}}^{region}={S}_{{{{\rm{TP}}}}}/{N}_{region}$), confirming that wetter regions are dynamically more stable. **d** Joint evolution of *S*_TP_ (black) and *S*_ENSO_ (blue). The maximum lagged Pearson correlation coefficient (*r* = 0.59, *p* < 0.001) occurs when *S*_TP_ lags *S*_ENSO_ by 6 months, highlighting ENSO modulation of SM entropy on the TP.

The entropy of the TP’s SM (*S*_TP_) declined significantly during 2000–2024 (Fig. 2a), with a slope of −0.000021 month^−1^ (*p* < 0.001), indicating an increasingly ordered system under prevailing wetting conditions. Consistent variations in *S*_TP_ are also found across multiple satellite-based SM datasets, further supporting the robustness of the results (Supplementary Fig. 5). A change in the rate of decline is suggested in the late 2010s, with entropy decreasing more rapidly after around 2018 than during the earlier period. This shift coincides with higher SM levels after 2018 compared to 2000–2018, supporting the interpretation that wetter states are associated with lower disorder (Fig. 2b). Spatial patterns reinforce this relationship: locations with higher mean SM (≥ 0.15 m^3^/m^3^) consistently exhibit lower entropy (Fig. 2c), suggesting that ongoing wetting enhances systemic organization across the Plateau.

Beyond the long-term ordering, *S*_TP_ exhibits pronounced interannual variability that is tightly synchronized with ENSO phases. El Niño years (e.g., 2004, 2015, 2023) correspond to sharp *S*_TP_ increases, indicating higher disorder, whereas La Niña years (2007, 2010, 2011, 2017) and the extended 2020–2022 triple La Niña coincide with marked reductions in *S*_TP_. To quantify this teleconnection, we calculated the entropy of the ENSO system using the temperature field over the Niño 3.4 region (*S*_ENSO_, see Methods). *S*_TP_ lags *S*_ENSO_ by about six months, yielding a peak correlation of 0.59 (*p* < 0.001) (Fig. 2d), indicating that tropical Pacific variability leaves a delayed and coherent imprint on TP soil moisture disorder. This connection is notable given the large geographical separation between the TP and the tropical Pacific, and the fact that it links entropy derived from temperature anomalies to entropy derived from soil moisture states. The signal is further supported by a significant positive correlation between *S*_TP_ and the ENSO index (*r* = 0.55, *p* < 0.01; Supplementary Fig. 6a), and is independently reproduced in CMIP6 historical simulations (Supplementary Fig. 6b). When restricted to ENSO-neutral periods, however, the relationship weakens substantially (*r* = 0.17, *p* > 0.05; Supplementary Figs. 6c, d), indicating that ENSO phases are the primary driver of the observed co-variability. Together, these results demonstrate a robust teleconnection, with ENSO phases exerting a clear modulation of SM entropy across the Plateau.

### ENSO-induced regional heterogeneity governs soil moisture disorder

**Fig. 3:**
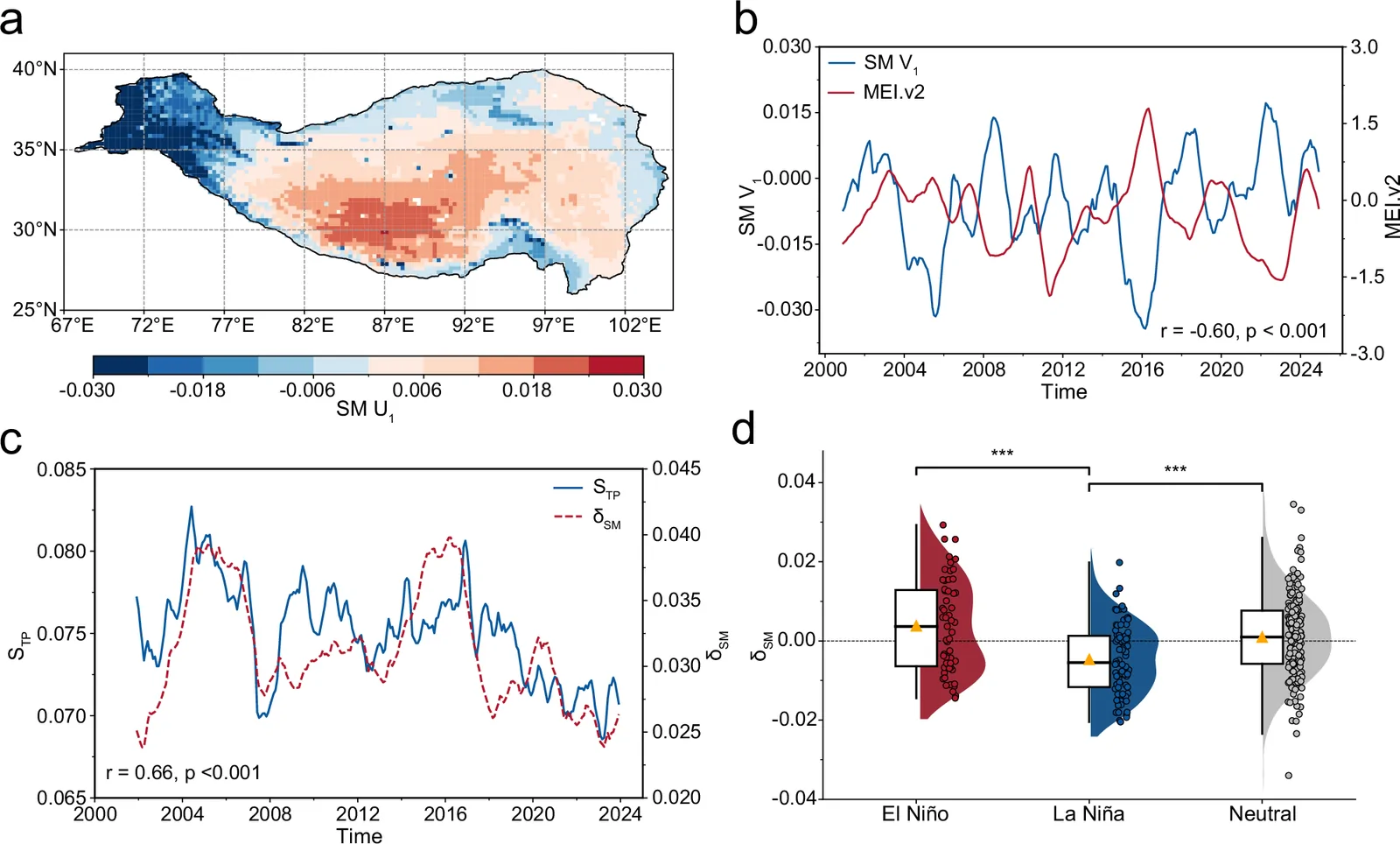
ENSO modulation of system entropy via soil moisture spatial heterogeneity. **a** Spatial patterns of the soil moisture first eigen microstate (SM *U*_1_), exhibiting a west−southwest dipole. **b** Temporal evolution of the SM first eigen microstate (SM *V*_1_, blue) and ENSO index (MEI.v2, red), showing a strong correlation (*r* = − 0.60, *p* < 0.001). **c** Coupling between entropy and spatial contrast. The co-evolution of *S*_TP_ (blue) and the inter-regional SM difference (*δ*_SM_, red; calculated as the SM in negative phase regions minus that in positive phase regions of *U*_1_, smoothed by a 3-year running mean to match the entropy time-window) shows that higher spatial heterogeneity corresponds to higher system entropy (*r* = 0.66, *p* < 0.001). **d** Modulation of spatial heterogeneity by ENSO phases. Violin plots of the inter-regional SM difference *δ*_SM_ (smoothed by a 2-month running mean to match MEI.v2), during El Niño (red), La Niña (blue), and neutral (gray) periods. Boxplots show the median, the 25th and 75th percentiles (box edges), the most extreme data points within 1.5 times the interquartile range (whiskers), and mean values (yellow triangles). Significant differences (***, *p* < 0.001) were identified between El Niño and La Niña, and between La Niña and neutral, based on both one-way ANOVA and two-sided Mann−Whitney *U*-test.

The mechanism underlying ENSO modulation can be understood from the leading eigen microstate of SM (EM1, Fig. 3a), which explains ~35% of total entropy variability (Supplementary Fig. 7). EM1’s temporal component *V*_1_ (Fig. 3b), smoothed with a 12-month moving average, shows a significant negative correlation with the Niño 3.4 index (*r* = − 0.60, *p* < 0.001) and dominant periodicities of 2–3.8 and 4.4–6 years that align with canonical ENSO cycles (Supplementary Fig. 8b). Pixel-wise correlations between the global temperature field and *V*_1_ further recover the characteristic ENSO pattern over the tropical Pacific (Supplementary Fig. 9), reinforcing the close relationship between ENSO and EM1. EM1’s spatial component *U*_1_ exhibits a pronounced dipole structure, with opposite phases in the western and southwestern TP (Fig. 3a). This dipole highlights the mechanism of ENSO influence: by enhancing or suppressing spatial heterogeneity, ENSO directly modulates SM entropy over the TP.

A series of connection analyses support this mechanism. As shown in Fig. 3c, *S*_TP_ correlates strongly with the SM difference between the positive- and negative-phase regions of *U*_1_ (*r* = 0.66, *p* < 0.001). This spatial asymmetry is modulated by ENSO intensity (*r* = 0.65, *p* < 0.001; Supplementary Fig. 10a), with El Niño (La Niña) corresponding to enhanced (reduced) contrasts and weak contrasts during neutral conditions (Fig. 3d). This linkage is further supported by the significant monthly tracking between the ENSO index and inter-regional differences (*r* = 0.37, *p* < 0.001; Supplementary Fig. 10b).

**Fig. 4:**
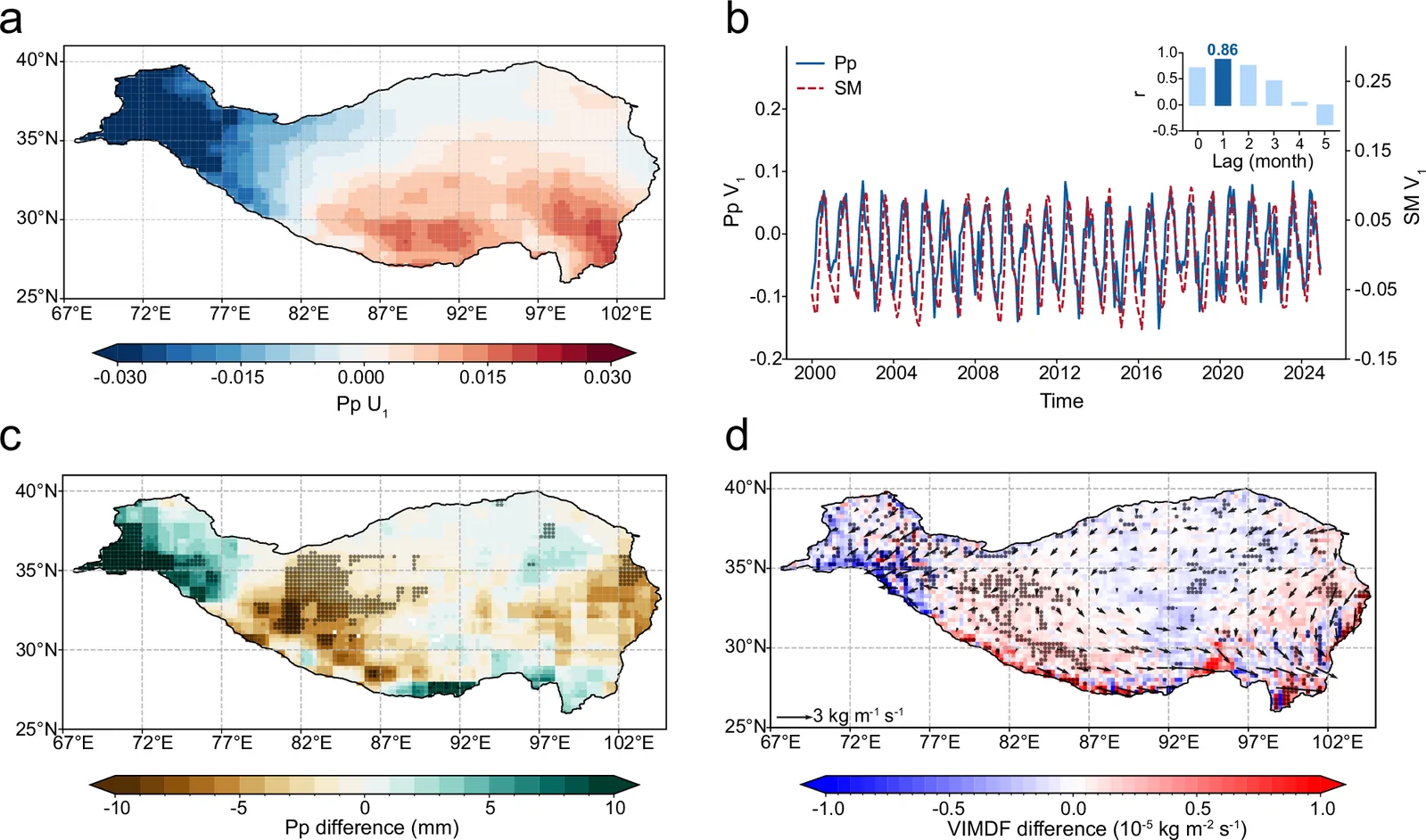
Precipitation anomalies as the primary driver of ENSO-induced soil moisture heterogeneity. **a** Spatial patterns of the precipitation first eigen microstate (Pp *U*_1_). **b** Temporal evolution of Pp first eigen microstate (Pp *V*_1_, blue) and soil moisture EM1 (SM *V*_1_, red), with a strong lagged correlation (*r* = 0.86, *p* < 0.001) peaking when SM lags Pp by one month, indicating a rapid SM response to precipitation variability in the TP. **c** ENSO fingerprint on precipitation. Mean monthly Pp anomaly difference between ENSO phases (El Niño minus La Niña). **d** Atmospheric circulation mechanism. Difference in vertically integrated water vapor flux anomaly (vectors) and its divergence anomaly (VIMDF, shaded) between ENSO phases (El Niño minus La Niña). Asterisks denote regions with statistically significant differences based on a two-tailed Welch’s *t*-test (*p* < 0.1).

The physical mechanisms underlying ENSO-driven inter-regional contrasts were further examined. Distinct moisture regimes characterize the dipole: the western TP exhibits a significant negative phase, with SM peaking in spring due to snowfall, spring melt, and low ET, whereas the southwestern TP shows a significant positive phase, with SM peaking in summer under the influence of monsoonal rainfall (Supplementary Fig. 8a). Strong spatial coherence between SM and Pp across the *U*_1_ field (*r* = 0.78, *p* < 0.001), along with temporal synchrony peaking at a one-month lag (*r* = 0.86, *p* < 0.001), demonstrates that Pp is the primary driver of SM variations (Figs. 4a, b). The inter-regional SM contrast closely tracks the Pp difference (Supplementary Fig. 11a, *r* = 0.70, *p* < 0.001). Moreover, annual Pp differences correlate significantly with the ENSO index (Supplementary Fig. 11b, *r* = 0.54, *p* < 0.01), suggesting that ENSO strongly modulates TP precipitation patterns.

Spatially, El Niño events tend to increase Pp over the western TP and reduce it over the southwestern TP, with La Niña generally producing the opposite pattern (Fig. 4c). ENSO-induced changes in moisture transport likely underpin this mechanism. The composite anomalies show that during El Niño events, southward moisture-flux anomalies and moisture convergence occur over the western TP, while westward and southward transport anomalies and moisture divergence are found over the southwestern Plateau (Fig. 4d). This pattern is consistent with previous findings that during El Niño events, the weakened and eastward-shifted Walker circulation induces descending motion over the eastern Indian Ocean and the Maritime Continent, which in turn excites a pair of anomalous anticyclones straddling the equator in the tropical Indian Ocean^66^. This configuration strengthens cyclonic anomalies over the Bay of Bengal and the South China Sea, weakening eastward and southward moisture transport over the southwestern TP. Simultaneously, it forms an anticyclonic ridge over the tropical north Indian Ocean that enhances northward moisture transport toward the western TP^38,39^.

By quantifying the system’s disorder, entropy offers a novel perspective on ENSO–SM teleconnections over the TP, revealing aspects of spatial organization that have received limited attention in previous studies of ENSO impacts on the plateau. The observed six-month lag in this teleconnection likely reflects the cumulative response of the hydrological system. Specifically, surface SM responds to Pp with a delay of approximately one month. In high-altitude regions where Pp falls as snow, snowmelt further postpones its increase^30,35^. Seasonal freeze-thaw cycles in the TP’s permafrost regions impose an additional delay on the adjustment of surface SM to climate anomalies^31^. Another potential explanation involves the gradual integration of spatially asynchronous seasonal hydrological anomalies. For instance, during El Niño phases, reduced summer rainfall in the southwestern TP and enhanced winter snowfall in the western region are incorporated over time, further delaying the adjustment of *S*_TP_.

### Projected drying and increasing disorder of the soil moisture system over the Tibetan Plateau

Rapid warming over the TP is expected to induce nonlinear responses in the SM system, potentially triggering abrupt regional shifts under future climate scenarios^12,18,24^. Projected increases in ENSO frequency and amplitude^67^ may further destabilize SM patterns through the teleconnections described above. Moreover, soil moisture–atmosphere coupling over the TP may propagate via Rossby wave pathways, potentially modulating remote climate extremes, including heatwaves over Europe and East Asia^24,27^, highlighting the importance of assessing projected TP soil moisture changes under warming scenarios. In complex systems, rising entropy has been proposed as a potential indicator consistent with early-warning behavior linked to reduced stability and preceding abrupt transitions across diverse contexts, from theoretical models to ecological systems^47,48^. To illustrate this, we consider a Local Facilitation Cellular Automaton (LFCA) model^68^, a spatially explicit model in which local interactions among neighboring cells give rise to a system-wide, bifurcation-like shift as a control parameter changes. Unlike the one-dimensional, time-series-based conceptual models commonly used to illustrate climate tipping point studies^11,43,44^, the LFCA model resolves the spatial organization of the system, so that the approaching transition is expressed through changes in spatial structure rather than in a single aggregated time series. This property makes it more representative of real-world complexity and well-suited to eco-hydrological processes such as vegetation-wetness interactions^48^. As shown in Supplementary Fig. 12, the system’s overall state declines gradually and then collapses as the control parameter decreases, while the entropy of system state (*S*_LFCA_) rises in advance of the threshold, signaling the approach of an abrupt transition.

**Fig. 5:**
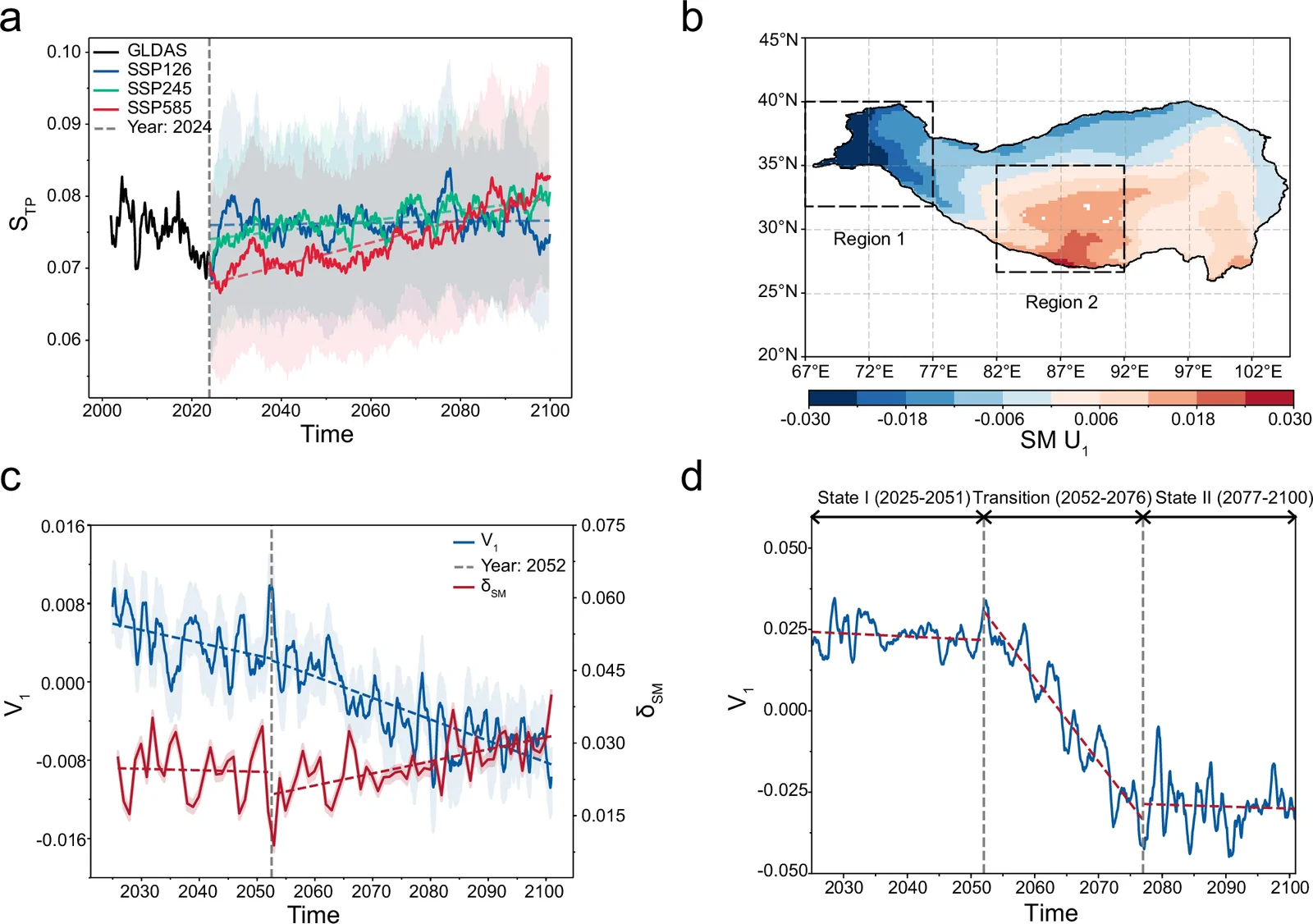
Projected changes in soil moisture system over the TP. **a** Observed (2000–2024, black) and projected (2025–2100) *S*_TP_ from CMIP6 models, with shading indicating the standard error. Unlike the historical decline, future scenarios (SSP126, SSP245, SSP585) consistently exhibit significant upward trends (*p* < 0.001), suggesting a shift towards a more disordered and unstable regime. **b** Multi-model ensemble mean of the leading soil-moisture eigen-microstate (*U*_1_) under SSP585, obtained by averaging the individual *U*_1_ patterns across the 12 selected CMIP6 models. Rectangles define the key dipole centers: Region 1 (western TP, negative phase) and Region 2 (southwestern TP, positive phase). **c** Ensemble-mean temporal evolution of the dominant SM mode (*V*_1_, blue) and the inter-regional SM difference (*δ*_SM_, red), with shading indicating the standard error. A critical breakpoint is identified in 2052 (grey dashed line) by segmented linear regression. *V*_1_ exhibits a significant downward trend after the breakpoint (*p* < 0.001), with a slope exceeding twice its previous value. No significant trend is observed before the breakpoint in *δ*_SM_, while a significant increase occurs thereafter (*p* < 0.001). **d** Representative trajectory of *V*_1_ from the IPSL-CM6A-LR model (SSP585), detailing the three distinct stages: a relatively stable state during 2025–2051, a rapid regime shift period (2052–2076, *p* < 0.001), and the settlement into a new stable state during 2077–2100.

We therefore turn to CMIP6 projections to assess how the TP’s SM entropy evolves under future warming. We analyzed monthly SM at 10 cm depth from the 12 CMIP6 models under SSP126, SSP245, and SSP585, selecting models that best reproduced the historical *S*_TP_ trajectories from the GLDAS-NOAH dataset during 2000–2024, based on trend, correlation, and variability criteria (see Methods). The ensemble mean of *S*_TP_ from these models shows strong agreement with observations (*r* = 0.68, *p* < 10^−4^; Supplementary Fig. 13), indicating that the selected ensemble captures the observed variability and is suitable for assessing future changes. Across scenarios, *S*_TP_ reveals a pronounced upward trend with warming (Fig. 5a), indicating a progressive rise in disorder and a heightened risk of reduced stability in the TP’s SM system. The projected rise in entropy corresponds to anticipated declines in annual SM anomalies, particularly under SSP585 (Supplementary Fig. 14). This rising entropy is rooted in a redistribution of the probabilistic weights {*p*_*I*_} among the system’s dominant spatiotemporal modes. Under future warming, the dominance of the uniform wetting mode is eroded, while the probabilistic weight of the dipole mode, representing regional contrast, increases. Because this redistribution balances the weights between competing modes, the resulting probability distribution becomes more uniform. Consequently, the dipole mode’s individual contribution to entropy (*c*_1_) grows (Supplementary Fig. 15), serving as a diagnostic marker of the system’s structural fragmentation and signaling a shift of the Asian Water Tower from a coherent hydrological state into a more disordered configuration. This amplification of regional contrast highlights the enhanced SM heterogeneity between Region 1 (western TP, negative phase) and Region 2 (southwestern TP, positive phase), underscoring the importance of regional contrasts in shaping future instability (Fig. 5b; Supplementary Fig. 16).

Consistent with the increase in *S*_TP_, additional evidence of potential regional shifts in the TP’s SM emerges from the temporal evolution of the dipole mode (*V*_1_) under the SSP585 scenario. Segmented regression of *V*_1_ reveals a significant breakpoint in the mid-century (around 2050, *p* < 0.001), followed by an accelerated decline at more than twice the pre-transition rate (Fig. 5c). Notably, *S*_TP_ also shows a significant shift during this period (Supplementary Fig. 17). Coupled with the spatial pattern *U*_1_ (Fig. 5b), the post-transition divergence between Region 1 and Region 2 drives a marked increase in regional heterogeneity, whereas differences prior to this breakpoint were negligible, as indicated by the red line in Fig. 5c. At the model level, IPSL-CM6A-LR exhibits a distinct regime shift (Fig. 5d): an initial modest decline during 2025–2051 (State I), a pronounced transition phase with rapid decrease during 2052–2076, and stabilization into a new state after 2077 (State II). Compared with State I, State II is characterized by widespread SM reductions across most of the Plateau, with the northeast remaining comparatively wetter (Supplementary Fig. 18). Notably, similar abrupt transitions have been identified in this model for snow-related variables under warming levels of 0.53–2.61 K^18^, underscoring the broader susceptibility of TP hydroclimate components to threshold-like responses.

## Discussion

Pronounced warming across the TP is driving substantial changes in regional hydrology, raising concerns over the stability of the Asian Water Tower^2^. Beyond local impacts, the TP is embedded in a network of teleconnections among potential climate tipping elements, including ENSO, the South Asian monsoon, and even the Amazon rainforest^10,12^. Destabilization in one component can trigger cascading responses in others, thereby amplifying systemic risks^69^. Our results provide new evidence for such coupling: SM entropy on the TP is modulated by ENSO, as evidenced by the six-month lagged correlation between *S*_TP_ and *S*_ENSO_ (Fig. 2d). We further trace the mechanism underlying this coupling: ENSO-induced precipitation anomalies imprint a west–southwest dipole on TP SM (Figs. 3a, 4a), and the strength of this inter-regional contrast governs the structural disorder of the SM system, as captured by the significant correlation between *δ*_SM_ and *S*_TP_ (Fig. 3c). The entropy framework is central to uncovering this chain. Conventional ENSO–TP assessments quantify hydroclimatic anomalies within individual subregions or seasons, such as developing-ENSO effects on southwestern TP summer rainfall^39^ and moisture sources for winter precipitation over the western Plateau^38^, focusing on the mean state or amplitude of local variability rather than how variability is organized across the Plateau. Such metrics cannot, by construction, represent how variability is organized across the Plateau as a whole. By measuring the degree of disorder in the spatiotemporal organization of the SM field, our entropy-based diagnostic instead resolves two features that these approaches miss: a system-level co-fluctuation of disorder between the ENSO and TP soil moisture systems, and the controlling role of cross-regional spatial heterogeneity in setting that disorder. This reframes the ENSO–TP relationship from a collection of local anomaly responses into a single, system-level coupling, and reinforces the view that the TP interacts dynamically with other global climate components, so that instabilities on the Plateau may trigger large-scale propagation through teleconnections^3,6,12^.

Future projections reveal a rising trend in *S*_TP_ accompanied by a decline in SM, in contrast to recent decades (Fig. 5a, Supplementary Fig. 14). Declining SM implies a reduction in hydrological buffering capacity, so that the system responds more strongly to external forcing such as drought and enhanced atmospheric evaporative demand^70^. As these responses amplify, system variability is no longer concentrated in a few coherent modes but becomes increasingly distributed across many competing eigen microstates, raising the entropy *S*_TP_. Because a more even distribution over eigen microstates reflects the loss of a dominant, coherent spatiotemporal pattern, this rise in disorder signals a decline in the predictability of the TP water cycle, suggesting that water-stressed conditions weaken the coherent organization that underpins the Asian Water Tower. Understanding this projected drying therefore requires examining the processes that govern SM recharge and retention over the TP. Continued warming is expected to intensify evapotranspiration losses (Supplementary Fig. 19a), while future precipitation over the TP is projected to become more intense and temporally concentrated, causing a larger fraction of rainfall to remain in surface water stores and thereby limiting effective SM recharge^71,72^. In addition, cryospheric processes, which are not fully represented in most CMIP6 models^73,74^, may further affect SM by reducing moisture inputs: declining snow water equivalent (SWE) and snowmelt (SWM) can reduce meltwater that sustains warm-season SM (Supplementary Figs. 19b, c)^31,75,76^, and permafrost degradation deepens the active layer and enhances downward percolation, reducing surface soil moisture^74,77^. Although glacial meltwater can transiently raise SM, as a finite store subject to peak water, projected to occur around mid-century in most High Mountain Asia basins and later under higher-emission scenarios^78^, its contribution is projected to decline through the second half of the century. Moreover, most meltwater is routed to runoff rather than recharging upper-layer SM. Because lower SM reduces buffering capacity and disperses variability across competing modes, these underrepresented processes imply that the SM-driven entropy increase (Fig. 5a), projected by CMIP6, may be conservative. Continued cryosphere–hydrology feedbacks are likely to further amplify the disorder of the TP hydroclimatic system and increase the risk of system instability.

Beyond this overall rise in disorder, our results reveal how the underlying spatial structure of the SM system reorganizes as entropy increases. The projected entropy rise is accompanied by an amplification of the dipole mode marked by strengthening west–southwest contrasts, indicating increasing spatial heterogeneity within the TP hydroclimatic system (Fig. 5b, Supplementary Fig. 15). Such enhanced heterogeneity is expected to exacerbate uneven water availability and intensify compound extremes, with cascading impacts on major downstream river basins in South and East Asia that collectively support nearly two billion people^1,2^. For example, spring SM anomalies in the western TP have been linked to extreme summer precipitation in the Yangtze River Basin, potentially intensifying downstream flood risk^29^. In the southwestern TP, SM deficits not only exacerbate vegetation degradation^22^ but also induce a positive land surface temperature feedback that contributes to regional warming^79^, and may further modify large-scale circulation by enhancing moisture transport toward the northern TP and raising the likelihood of extreme precipitation^80^. These contrasting impacts of the two branches underscore that future assessments of TP hydroclimatic stability should move beyond total water availability and treat spatial heterogeneity as a key dimension.

Crucially, this growing disorder may not unfold gradually. The projected rise in entropy reproduces the early-warning signals of critical transitions observed in conceptual models, pointing to a heightened risk of abrupt, nonlinear shifts rather than smooth change in TP hydroclimate (Supplementary Fig. 12)^18^. Consistent with this, segmented regression identifies a breakpoint in both entropy and the dipole-mode evolution (*V*_1_) around the mid-21st century under high-emission scenarios, after which spatial contrast intensifies sharply (Fig. 5c, Supplementary Fig. 17). This tendency is most pronounced in the IPSL-CM6A-LR model, which undergoes a regime-shift-like transition: a rapid mid-century decline into a persistently drier state, except for relatively wetter conditions in the northeast (Fig. 5d, Supplementary Fig. 18). Such threshold behavior is consistent with the interacting positive feedbacks that amplify perturbations and progressively push the system toward a critical threshold^11^. A fast atmospheric feedback, in which reduced SM shifts the surface energy balance toward sensible heating and reinforces land–atmosphere warming^24^, can combine with a slower ecological feedback, in which progressive drying favors stress-tolerant vegetation with reduced water retention^46^. The convergence of these fast and slow adjustments can progressively destabilize the SM system, providing a physical basis for the abrupt, threshold-like response captured by the entropy diagnostic and reinforcing that the projected instability risk reflects a potential regime shift rather than gradual drying alone.

Several boundaries of interpretation should nonetheless be kept in mind. First, entropy is not interpreted here as a direct measure of stability, such as the recovery rate in the CSD framework^11^, but as a diagnostic of the system’s degree of structural organization. Higher disorder reflects a progressive dispersion of variability across multiple competing modes, which in complex, high-dimensional systems is commonly associated with the loss of coherent organization, reduced resilience, and heightened sensitivity to perturbations^48,56,81,82^. We therefore frame our results as indicating a heightened risk of nonlinear transition rather than evidence that tipping is inevitable. Second, because the Asian Water Tower is defined by its cryospheric water storage, the fidelity of the future projections depends on how well these processes are represented. CMIP6 models, while grounded in our current understanding of Earth system processes, may not fully capture system behavior under future climate states, particularly given known limitations in representing TP cryospheric dynamics and land–atmosphere coupling^73,74^. Accordingly, while Pp emerges as the primary driver of projected entropy changes, the contributions of snow accumulation, glacier melt, permafrost thaw, and surface runoff to entropy dynamics remain insufficiently assessed and constitute an important source of uncertainty. Finally, the clearest regime-shift signal is largely confined to a particular model (IPSL-CM6A-LR), whereas other models show more gradual responses, leaving open whether such signals represent genuine thresholds or model-specific behavior. Resolving these questions will require improved representation of cryospheric, hydrological, and land–atmosphere processes together with coordinated multi-model assessments.

A further source of uncertainty lies in the observational data themselves. The in-situ SM network over the TP remains sparse, with limited spatial coverage, a relatively short record (2009–2019), and measurements largely confined to the upper 5−10 cm of the soil profile. These gaps are most acute in permafrost regions, which are important to the TP water balance yet remain severely undersampled. Because permafrost governs a substantial fraction of the Plateau’s frozen-water storage and freeze–thaw dynamics, we explicitly tested its influence by recomputing entropy with permafrost areas excluded. The resulting entropy evolution and ENSO teleconnection remained broadly consistent with the full-domain analysis (Supplementary Fig. 20), indicating that our principal conclusions are robust to uncertainties in permafrost-region SM estimates. The somewhat longer lagged response found in permafrost regions is itself physically consistent with the role of freeze–thaw processes in prolonging SM memory, lending additional physical coherence to the diagnosed ENSO–TP coupling. Beyond the in-situ network, residual uncertainties in the underlying assimilated SM product cannot be fully excluded over the TP’s cold, high-elevation, and topographically complex terrain, where SM estimation remains challenging for most reanalysis and model-based datasets^33,34^. The relatively coarse resolution of GLDAS-NOAH (0.25^∘^ × 0.25^∘^) may further dampen localized SM gradients and sub-grid structure across steep elevation gradients and heterogeneous land surfaces^83,84^, which could influence the absolute magnitude of the estimated entropy. Because our analysis targets the temporal evolution and large-scale spatial organization of entropy rather than its absolute value, such smoothing does not materially affect the dynamics on which our conclusions rest. Consistent with this, the SM entropy evolution remains stable across independent satellite products at substantially finer resolutions (0.25^∘^ and 500 m, Supplementary Fig. 5), and the dominant dipole and its ENSO linkage emerge at spatial scales far larger than the native grid, reflecting a collective, system-level mode rather than local-scale variability. Integrating higher-resolution observations and improved cold-region land-surface simulations would further enable the entropy framework to resolve finer-scale hydroclimatic heterogeneity across the TP.

Collectively, these findings underscore the urgency of sustained monitoring of the TP’s SM and improved representation of land–climate feedbacks in Earth system models, particularly with respect to cryospheric processes and teleconnections. More broadly, our work highlights the value of entropy-based diagnostics for characterizing the risk of hydrological instability. Applying such frameworks to other climate-sensitive regions may provide a powerful tool for anticipating tipping-like behavior in the terrestrial water cycle and its cascading impacts on society.

## Methods

### Data

#### Soil moisture datasets

This study integrates three complementary sources of SM data: (1) in-situ observations, (2) reanalysis products, and (3) land surface model outputs. In-situ SM observations were obtained from the Tibetan Plateau Observatory (Tibet-Obs)^63^, a ground-based monitoring network that spans major climatic regimes: cold arid (Ali, 4 sites; Shiquanhe, 20 sites), semi-arid (Naqu, 11 sites), and humid (Maqu, 26 sites) climates (Supplementary Fig. 1). Surface SM was measured at 5 cm depth and 15-min resolution during 2009–2019. To ensure consistency with gridded products, the observations were aggregated to monthly means and mapped to a 0.25^°^ × 0.25^°^ grid.

Reanalysis and land surface model SM datasets were used, including the Modern-Era Retrospective Analysis for Research and Applications Version 2 (MERRA-2), the ERA5-Land reanalysis dataset produced by the European Centre for Medium-Range Weather Forecasts, and the Global Land Data Assimilation System Noah Land Surface Model version 2.1 (GLDAS-NOAH). MERRA-2 provides monthly SM at 0.5^∘^ × 0.625^∘^ resolution across two soil layers: 0–5 cm and 0–100 cm. ERA5-Land offers monthly SM at 0.1^∘^ resolution across four layers: 0–7 cm, 7–28 cm, 28–100 cm, and 100–289 cm. GLDAS-NOAH provides monthly SM at 0.25^∘^ resolution with four layers: 0–10 cm, 10–40 cm, 40–100 cm, and 100–200 cm. This study focuses on the topsoil layer for each product: 0–5 cm (MERRA-2), 0–7 cm (ERA5-Land), 0–10 cm (GLDAS-NOAH), over the common analysis period 2000–2024. All datasets were resampled to a uniform 0.25^∘^ resolution, and GLDAS-NOAH SM values were converted from mass (kg m^−2^) to volumetric units (m^3^ m^−3^) for inter-dataset consistency. In addition, two satellite-based surface SM datasets were incorporated to validate the consistency of the identified SM entropy evolution in the TP: a 0.25^∘^ product (2002–2015) based on ECV microwave products^85^, and a 500m product (2015–2021) derived from SMAP microwave data^86^.

#### Meteorological data

This study employs monthly meteorological variables including precipitation (Pp) and evapotranspiration (ET) from the GLDAS-NOAH dataset provided at 0.25^∘^ × 0.25^∘^ resolution. Near-surface (1000-hPa) air temperature from ERA5 was used to assess the spatial covariability between ENSO signals and SM *V*_1_. The monthly total column vertically integrated water vapor flux and its divergence from ERA5 were used to investigate ENSO-related moisture transport influencing Pp over the TP. ENSO variability was quantified using the Multivariate ENSO Index Version 2 (MEI.v2), which combines sea level pressure, sea surface temperature, low-level winds, and outgoing longwave radiation to provide a physically consistent representation of coupled ocean–atmosphere dynamics^87^. Since MEI.v2 does not define standardized thresholds for the onset or classification of ENSO events, the Oceanic Niño Index (ONI), defined as the 3-month running mean of SST anomalies in the Niño 3.4 region, was additionally used to identify and categorize ENSO phases. El Niño (La Niña) events are defined as periods when the ONI exceeds +0.5^∘^C (falls below −0.5^∘^C) for at least five consecutive months.

#### CMIP6 model simulations

We selected CMIP6 models for future projection analysis using a two-tier quantitative criterion based on performance over the historical overlap period (2000–2014). Specifically, the monthly surface soil moisture variable (mrsos) was extracted from each CMIP6 model to compute *S*_TP_. Two metrics were computed for each model relative to the GLDAS-NOAH reference: (1) the Pearson temporal correlation *R*_*T*_ between the model’s *S*_TP_ and the GLDAS *S*_TP_, and (2) the spectral correlation *R*_*F*_, defined as the Pearson correlation between the spectral amplitudes of the two standardized *S*_TP_ series. Unlike *R*_*T*_, *R*_*F*_ is insensitive to temporal phase offsets caused by differences in the internal variability chronology between the model and the real world.

Because CMIP6 historical simulations are free-running (uninitialized)^88,89^, phase-scrambling tests (50,000 surrogates preserving the GLDAS power spectral density) confirm that *σ*(*R*_*T*_) ≈ 0.35 for this series length, meaning *R*_*T*_ alone cannot reliably distinguish a model with correct characteristic timescales from a random-phase surrogate. We therefore applied a two-tier criterion. Tier 1 retains models with a statistically significant positive temporal correlation (*R*_*T*_ > 0, *p* < 0.1, one-tailed, *N* = 145). Tier 2 rescues models whose temporal correlation is computationally zero (∣*R*_*T*_∣ < 0.01) but whose spectral structure closely matches GLDAS (*R*_*F*_ > 0.80).

To ensure inter-model comparability, the mrsos outputs from all models were resampled to a common 0.25^∘^ × 0.25^∘^ grid using bilinear interpolation and converted from mass kg m^−2^ to volumetric units (m^3^ m^−3^). A final ensemble of 12 models was obtained by applying the above criterion to the initial 25 widely used CMIP6 models (see Supplementary Table 1). The ensemble mean of the selected models achieves *R*_*T*_ = 0.68 against GLDAS-NOAH (*p* < 10^−4^). Results are insensitive to the exact threshold values, as CMIP6 models selected under different threshold configurations consistently reproduce the historical *S*_TP_ trajectories and yield projected future changes that are highly consistent with those shown in Fig. 5 (see Supplementary Table 4 and Supplementary Fig. 22). To ensure internal consistency across hydroclimatic variables, other relevant outputs–including near-surface air temperature (tas), surface snow water equivalent (snw), surface snowmelt flux (snm), and total evaporation flux (evspsbl)–were extracted concurrently from the same final ensemble of 12 models. Detailed descriptions of the model selection procedure are provided in Supplementary Note S1.

### Evaluation of soil moisture data applicability

To ensure that entropy estimation and subsequent dynamical analyses are grounded in physically meaningful SM behavior, we evaluated the applicability of three commonly used SM products (GLDAS-NOAH, MERRA-2, and ERA5-Land) over the TP. Rather than focusing on conventional accuracy, the goal is to assess whether these datasets retain the temporal variability and memory characteristics that underpin eigen microstate decomposition and teleconnection analyses.

Two complementary aspects were examined. First, absolute agreement was assessed using three metrics that reflect consistency in mean states and overall temporal variability between gridded and in-situ observations: the temporal Pearson correlation coefficient (*R*_*T*_), root mean square error (RMSE), and bias (Bias). Together, they indicate whether the products reproduce the magnitude and basic temporal behavior of observed SM, thereby preventing systematic offsets from propagating into entropy calculations. Second, dynamical similarity was evaluated using metrics that characterize temporal structure. The spectral Pearson correlation coefficient (*R*_*F*_) measures the agreement in frequency-dependent variability, ensuring the preservation of dominant oscillatory components contributing to eigen microstates. The autocorrelation structure correlation (*R*_*ACF*_) quantifies similarity in short- to medium-term memory by comparing autocorrelation functions over the first 10 lags, representative of month-scale persistence in the TP’s SM. These properties are critical because the entropy framework is sensitive to the distribution and persistence of spatiotemporal fluctuations.

Together, these five metrics provide a targeted evaluation of whether SM products maintain the key statistical properties required for reliable entropy estimation and dynamical interpretation. Full definitions and calculation formulas are summarized in Supplementary Table 2.

### Eigen microstate entropy

To quantify the degree of disorder of the TP soil moisture system, we adapt the eigen microstate theory (EMT)^50,56,90^, a statistical framework originally developed to identify emergent large-scale organization and critical transitions in non-equilibrium systems across disciplines such as physics^51,53^, climate dynamics^52,91^, neuroscience^92^, and ecology^54,55^. Here, we adapt this framework to define an entropy metric that directly characterizes the disorder in SM dynamics.

We represent the system as *N* spatial units, each with a state variable *X*_*i*_(*t*) at discrete time *t*. The evolving system state is expressed as a sequence of microstates ***X***(*t*), which collectively form a normalized data matrix ${{{\boldsymbol{A}}}}\in {{\mathbb{R}}}^{N\times M}$:

1 $${{{{\boldsymbol{A}}}}}_{it}=\frac{{X}_{i}(t)}{\sqrt{{C}_{0}}},\qquad \,{C}_{0}={\sum }_{t=1}^{M}{\sum }_{i=1}^{N}{X}_{i}^{2}(t),$$

such that ${{{\rm{Tr}}}}({{{\boldsymbol{A}}}}{{{{\boldsymbol{A}}}}}^{\top })=1$. In our analysis, ${X}_{i}(t)=\sqrt{{Y}_{i}(t)}$ where *Y*_*i*_(*t*) denotes SM or Pp. We then decompose the system using singular value decomposition: ${{{\boldsymbol{A}}}}={\sum }_{I=1}^{N}{\sigma }_{I}{{{{\boldsymbol{U}}}}}_{I}\otimes {{{{\boldsymbol{V}}}}}_{I},$ where ***U***_*I*_ and ***V***_*I*_ represent the spatial and temporal structures of the *I*-th eigen microstate, respectively, and *σ*_*I*_ are the corresponding singular values satisfying ${\sum }_{I}{\sigma }_{I}^{2}=1$. ${p}_{I}={\sigma }_{I}^{2}$ quantifies the fraction of system dynamics explained by each mode and can be interpreted as the probability of the system occupying the *I*-th eigen microstate. This representation effectively reduces the high-dimensional SM field to a set of dominant, independent spatial–temporal patterns with interpretable physical meaning. A detailed description of the EMT is provided in Supplementary Note S2.

Building on the probability distribution of eigen microstates {*p*_*I*_}, we define a Shannon-type entropy:

2 $${S}_{{{{\rm{EM}}}}}=-\mathop{\sum }_{I=1}^{N}{p}_{I}\ln {p}_{I},$$

which measures how evenly the system’s dynamics are distributed among the eigen microstates. Low *S*_EM_ indicates that variability is concentrated in one or a few coherent modes, reflecting an ordered system with strong organization. High *S*_EM_ reflects a more uniform distribution with many competing modes, corresponding to disordered or noisy states with no dominant spatial–temporal pattern^56^. In the theoretical limit of a perfectly ordered system (e.g., condensation into a single dominant eigen microstate), *p*_1_ → 1 and *S*_EM_ → 0. Conversely, in a maximally disordered system (e.g., purely random system) with all eigen microstates contributing equally, *p*_*I*_ → 1/*N* and ${S}_{{{{\rm{EM}}}}}\to \ln N$. Therefore, *S*_EM_ serves as a sensitive and interpretable measure of disorder.

To pinpoint the specific structure driving systemic disorder, we decompose the total entropy into contributions from individual eigen microstates. The fractional contribution *c*_*I*_ of the *I-*th eigen microstate is defined as:

3 $${c}_{I}={s}_{I}/{S}_{{{{\rm{EM}}}}},\qquad {s}_{I}=-{p}_{I}\ln ({p}_{I}),$$

This decomposition serves as a diagnostic tool, allowing us to isolate the dominant modes responsible for the system’s disorder. By ranking the eigen microstates in descending order of *c*_*I*_, we identify the primary spatial patterns (or dominant modes) that govern the system’s structural complexity.

To quantify the temporal evolution of entropy, moving-window analyses were performed using variable-specific parameter settings. For soil moisture entropy (*S*_TP_), a 3-year moving window with a 1-month step was adopted. This window provides sufficient samples for robust entropy estimation while preserving the characteristic interannual variability associated with ENSO-related soil moisture dynamics. For ENSO entropy (*S*_ENSO_), a 1-year moving window with the same monthly step was used to resolve individual ENSO events while avoiding signal cancellation caused by blending successive opposite-phase ENSO events. The rationale for these window selections and the associated sensitivity analyses are provided in Supplementary Note S3.

### Local facilitation cellular automaton model

Traditional tipping point analyses often rely on one-dimensional, time-series-based conceptual models^11,43^, which are inherently limited to a single state variable and cannot represent spatial interactions or multi-component feedbacks. However, many real-world eco-hydrological systems, including vegetation–soil moisture coupling, exhibit high-dimensional spatial organization and nonlinear local facilitation processes that can drive abrupt regime shifts. To illustrate how entropy behaves in such systems approaching an abrupt transition, we employ a Local Facilitation Cellular Automaton (LFCA) model^68^, which provides a generic yet powerful representation of spatially coupled, nonlinear dynamics with bifurcation-like behavior.

Following ref. ^48^, the LFCA model evolves on a 100 × 100 lattice in discrete time. Each cell can occupy one of three possible states: −1, 0, or +1. State transitions are probabilistic and depend both on the local neighborhood and on the global state fraction, capturing the interplay between short-range facilitation and system-wide conditions. The four possible transitions are +1 *⇌* 0 *⇌* − 1, with transition probabilities defined as

4 $${w}_{+| 0}=m,$$

5 $${w}_{0|+}=\left[\delta {\rho }_{+}+(1-\delta ){q}_{+| 0}\right](b-c{\rho }_{+}),$$

6 $${w}_{-| 0}=r+f{q}_{+| -},$$

7 $${w}_{0| -}=d,$$

where *q*_*A*∣*B*_ denotes the fraction of neighboring units in state *A* around a unit in state *B*, and *ρ*_+_ is the global fraction of +1 units. The parameters *m*, *r*, *f*, *d*, *δ*, and *c* are constants (see Supplementary Table 3). The parameter *b* serves as the control parameter, and as it decreases, the system undergoes an abrupt shift in its global state, allowing early-warning signals, such as rising entropy, to be observed prior to the transition. This rise in entropy emerges because the dominant pattern progressively weakens near the transition, allowing multiple competing modes to coexist, which increases the number of effective modes contributing to the dynamics.

## Supplementary information

Supplementary Information

Transparent Peer Review file

## Data Availability

The datasets generated in this study have been deposited in Zenodo under accession code https://doi.org/10.5281/zenodo.21713077. The datasets used in this study are publicly available from the following sources. The GLDAS-NOAH data used here are publicly available at https://disc.gsfc.nasa.gov/datasets/GLDAS_NOAH025_M_2.1/summary?keywords=GLDAS. The ERA5 reanalysis data are publicly available at https://cds.climate.copernicus.eu/datasets/reanalysis-era5-pressure-levels?tab=download. The MERRA-2 reanalysis data are publicly available at https://disc.gsfc.nasa.gov/datasets/M2TMNXLND_5.12.4/summary?keywords=M2CONXLND. The ERA5-Land reanalysis data are publicly available at https://cds.climate.copernicus.eu/datasets/reanalysis-era5-land-monthly-means?tab=download. The CMIP6 data are publicly available at https://aims2.llnl.gov/search. The ONI data are publicly available at https://origin.cpc.ncep.noaa.gov/products/analysis_monitoring/ensostuff/ONI_v5.php. The MEI.v2 data are publicly available at https://psl.noaa.gov/enso/mei/. The 0.25^∘^ satellite-based SM dataset is publicly available at https://doi.org/10.6084/m9.figshare.7996448. The 500m satellite-based SM dataset is publicly available at https://doi.org/10.11888/Terre.tpdc.302216. The permafrost classification dataset is publicly available at https://nsidc.org/data/ggd318/versions/2#anchor-data-access-tools. The elevation dataset of the Tibetan Plateau is publicly available at https://www.earthenv.org/topography. The boundary dataset of the Tibetan Plateau is publicly available from the National Tibetan Plateau/Third Pole Environment Data Center at https://cstr.cn/18406.11.Geogra.tpdc.2700.

## Code availability

The Python codes used for analysis and figure generation are available at https://doi.org/10.5281/zenodo.21713077.
