# Supplementary material for: Warming-driven rise in soil moisture entropy signals growing instability risk in the Asian Water Tower
Source: Nat Commun. 2026 Aug 21;17:10012. doi: 10.1038/s41467-026-76955-w (PMC13590596; doi:10.1038/s41467-026-76955-w)
Supplement: Supplementary file 1 [file 41467_2026_76955_MOESM1_ESM.pdf]

# Supplementary Information for "Warming-driven rise in soil moisture entropy signals growing instability risk in the Asian Water Tower"


Yiran Xie[1], Teng Liu[2,3,4*], Xuan Ma[5], Yingshuo Lyu[6], Xu Wang[1], Yatong Qian[2], Yongwen Zhang[7,8], Ming Wang[1,9*], Xiaosong Chen[2,10*]

[1]School of National Safety and Emergency Management, Beijing Normal University, Beijing, 100875, China.
[2]School of Systems Science and Institute of Nonequilibrium Systems, Beijing Normal University, Beijing, 100875, China.
[3]Munich Climate Center and Earth System Modelling Group, Department of Aerospace and Geodesy, TUM School of Engineering and Design, Technical University of Munich, Munich, 80333, Germany.
[4]Potsdam Institute for Climate Impact Research, Potsdam, 14473, Germany.
[5]State Key Laboratory of Disaster Weather Science and Technology, Chinese Academy of Meteorological Sciences, Beijing, 100080, China.
[6]Department of Earth System Science, Ministry of Education Key Laboratory for Earth System Modeling, Institute for Global Change Studies, Tsinghua University, Beijing, 100084, China.
[7]Yunnan Key Laboratory of Complex Systems and Brain-Inspired Intelligence, Kunming University of Science and Technology, Kunming, 650500, China.
[8]Faculty of Science, Kunming University of Science and Technology, Kunming, 650500, China.
[9]Joint International Research Laboratory of Catastrophe Simulation and Systemic Risk Governance, Beijing Normal University, Zhuhai, 519087, China.
[10]Institute for Advanced Study in Physics and School of Physics, Zhejiang University, Hangzhou, 310058, China.

*Corresponding author(s). E-mail(s): teng.liu@tum.de; wangming@bnu.edu.cn; chenxs@zju.edu.cn;

# Contents of this file


**Supplementary Figures** 3

**Supplementary Tables** 23

**S1 CMIP6 Model Selection** 26

S1.1 Phase-scrambling null distribution . . . . . 26

S1.2 Spectral correlation $R_F$ . . . . . 26

S1.3 Threshold justification . . . . . 26

S1.4 Sensitivity of results to threshold choice . . . . . 26

**S2 Eigen Microstates Theory** 28

**S3 Rationale for moving-window selection** 29

S3.1 Three-year window for $S_{\mathrm{TP}}$ . . . . . 29

S3.2 One-year window for $S_{\mathrm{ENSO}}$ . . . . . 29

S3.3 Two-month window for correlation analysis . . . . . 29

# Supplementary Figures

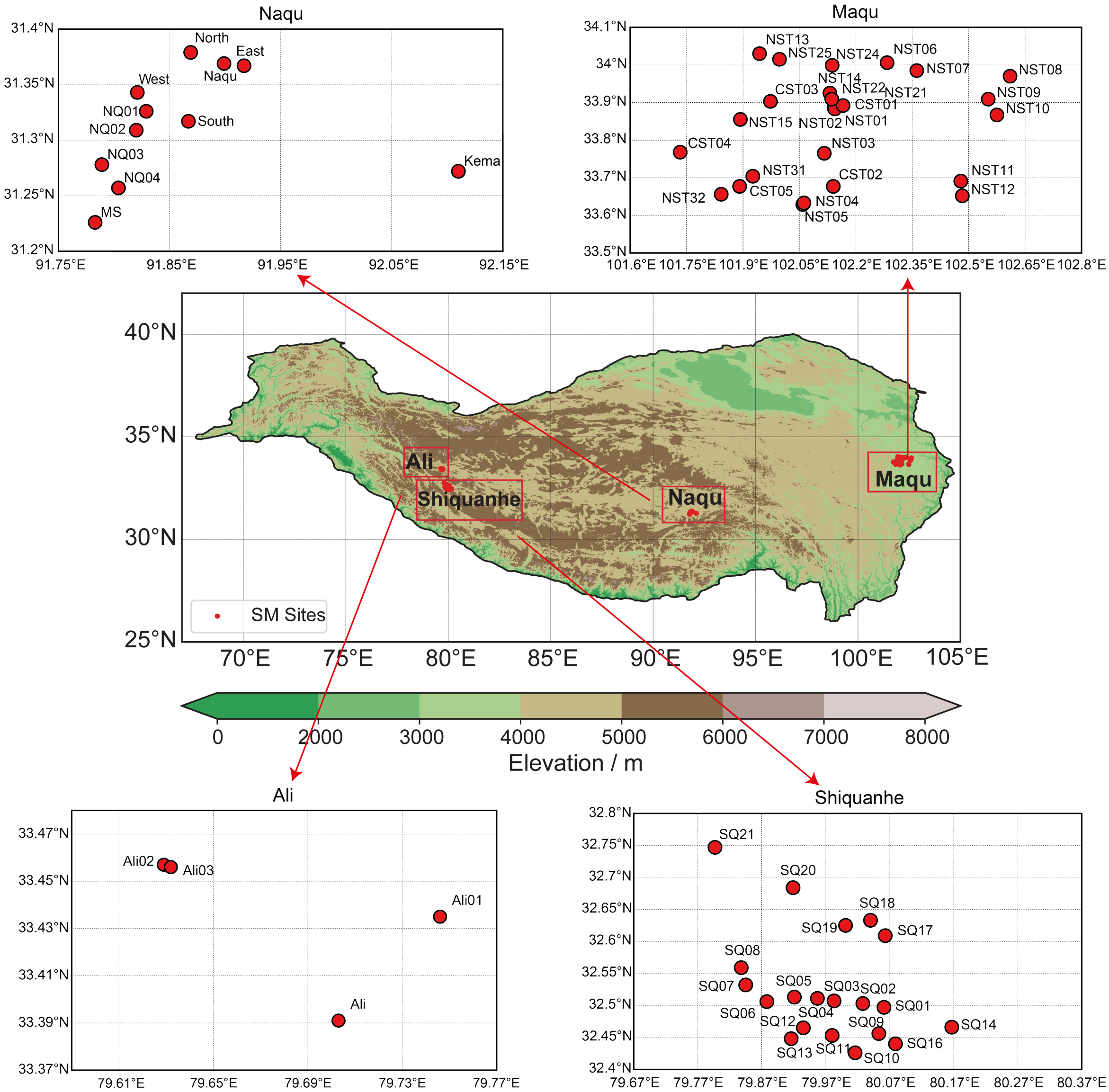


**Fig. S1 Distribution of SM observation sites from the Tibet-Obs network.** Red rectangles indicate the four regional-scale SM monitoring networks–Maqu, Naqu, Ali, and Shiquanhe–while red dots show individual observation sites.

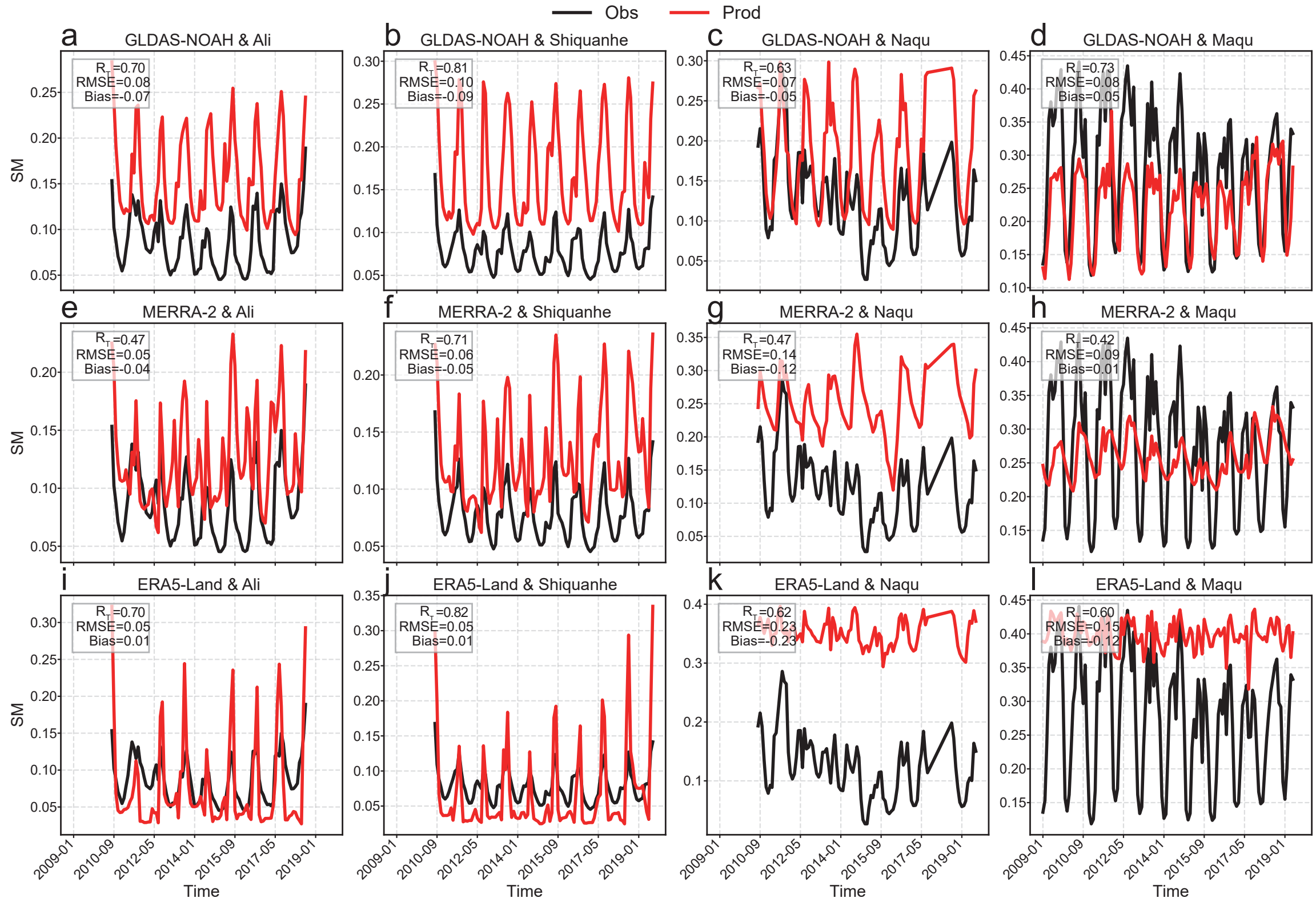


**Fig. S2 Monthly SM evolution from observations and products across four TP subregions.** Panels (**a**)–(**d**) show comparisons between GLDAS-NOAH and observations at Ali, Shiquanhe, Naqu, and Maqu, respectively. Panels (**e**)–(**h**) replicate the comparisons shown in (**a**)–(**d**) using MERRA-2 data, and Panels (**i**)–(**l**) do so using ERA5-Land data.

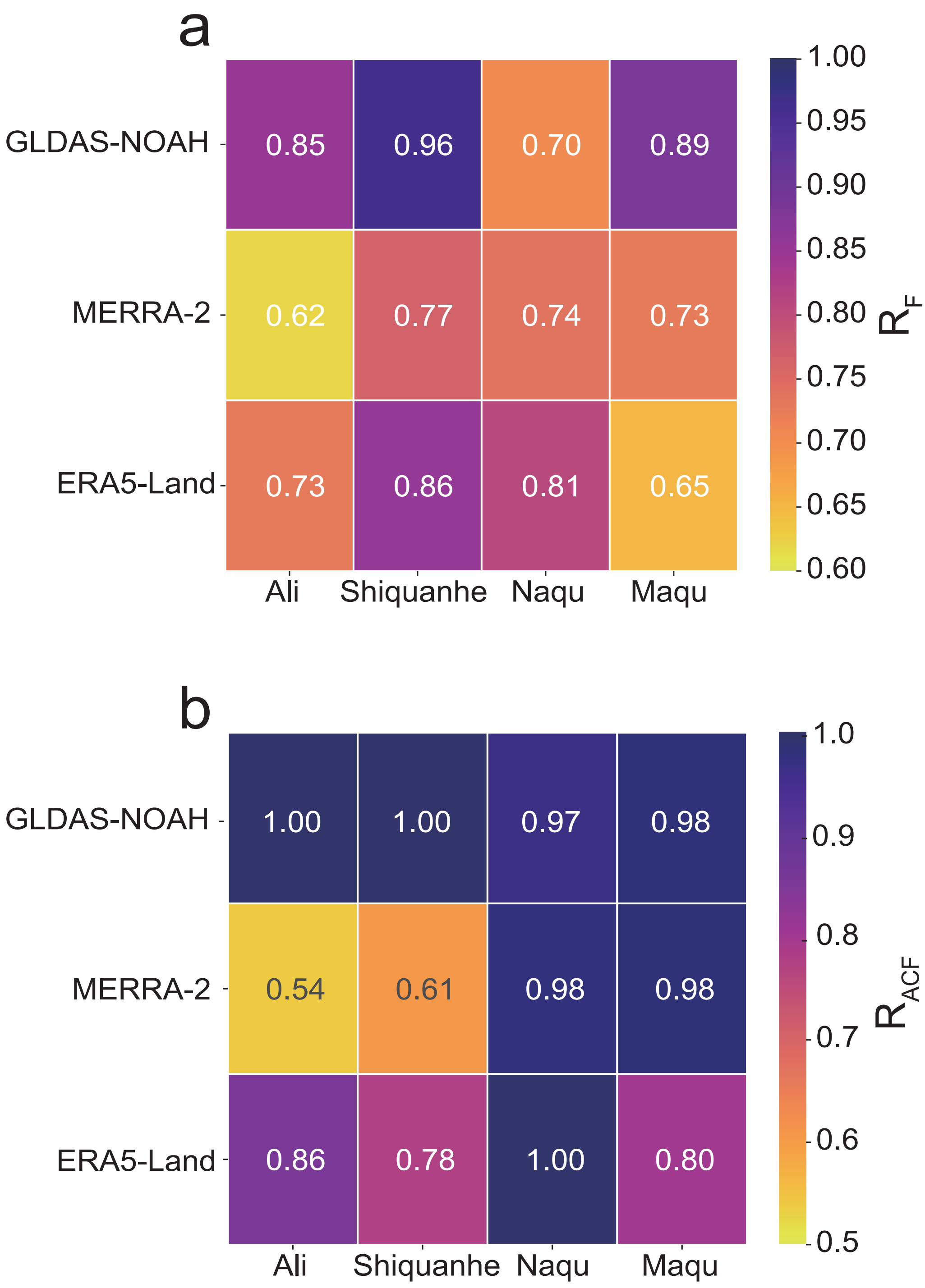


**Fig. S3 Spectral and autocorrelation similarity of SM between observations and products across four TP regions.** (**a**) Correlation heatmaps of Fourier spectra. (**b**) Correlation heatmaps of autocorrelation sequences (AC1-AC10).

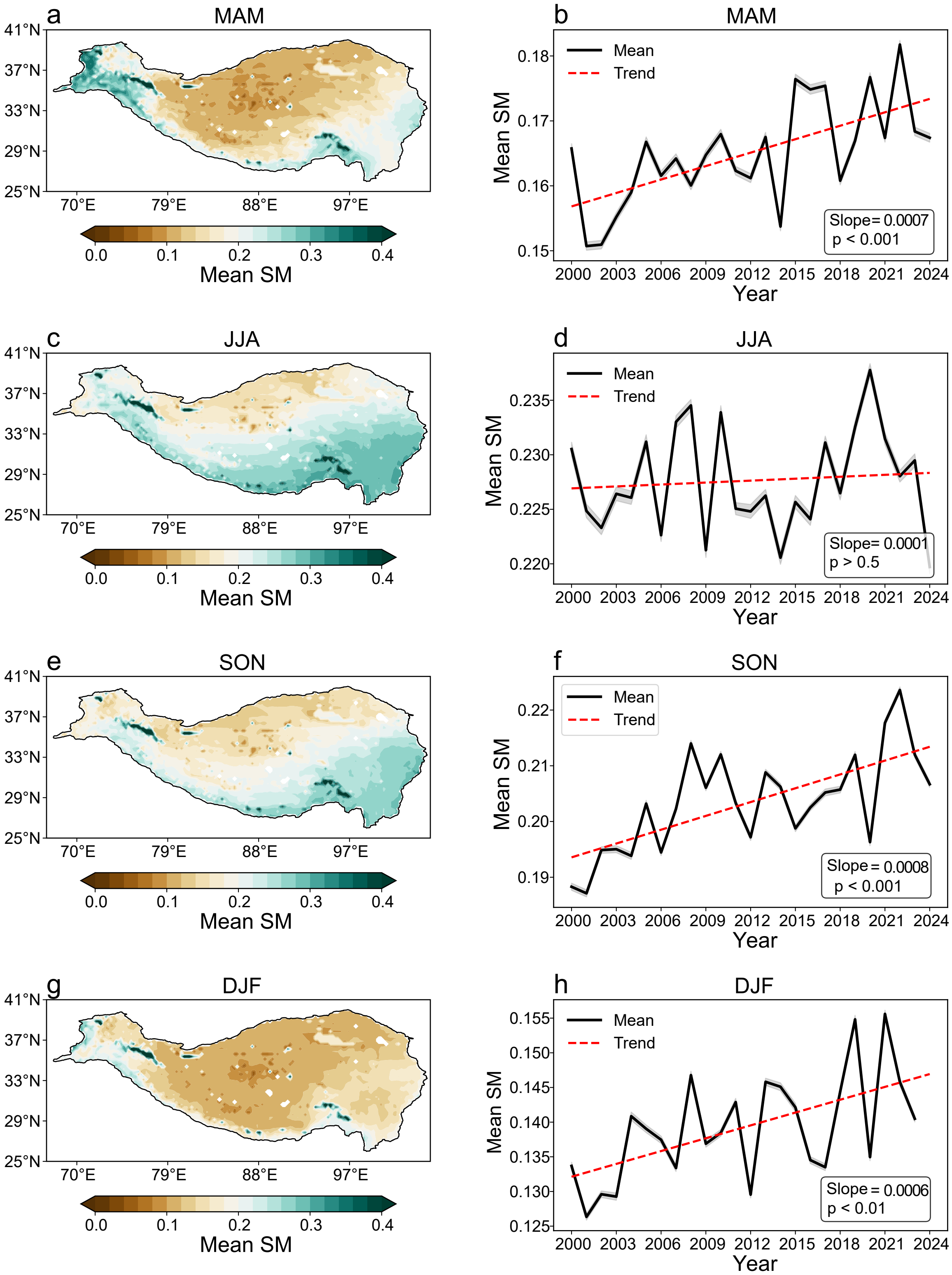


**Fig. S4 Spatiotemporal dynamics of SM over the TP across different seasons.** (**a**), (**c**), (**e**), (**g**) Spatial distribution of mean SM for spring (March-May, MAM), summer (June-August, JJA), autumn (September-November, SON) and winter (December-February, DJF), respectively. (**b**), (**d**), (**f**), (**h**) Temporal evolution of the annual mean SM for spring, summer, autumn and winter, respectively. Shaded areas in the temporal evolution plots represent the standard error.

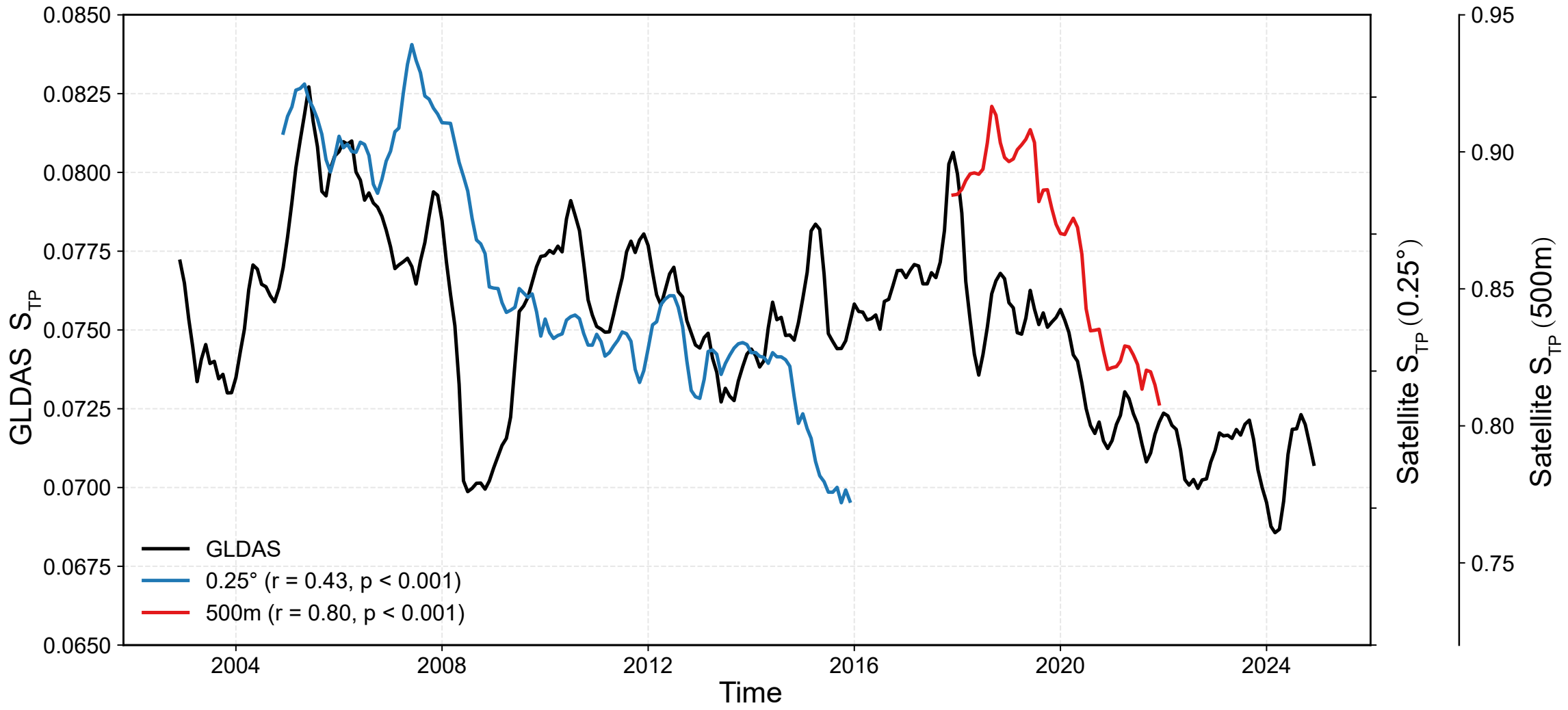


**Fig. S5 Consistent temporal evolution of $S_{TP}$ across multiple SM datasets.** Black line denotes GLDAS-NOAH SM. Blue and red lines represent TP satellite-based SM products at 0.25° (2002–2015) and 500m (2015–2021), respectively, both showing significant positive correlations with GLDAS-NOAH. For the 500 m dataset, the evaluation was restricted to 2015–2021 to align with its available in-situ observations.

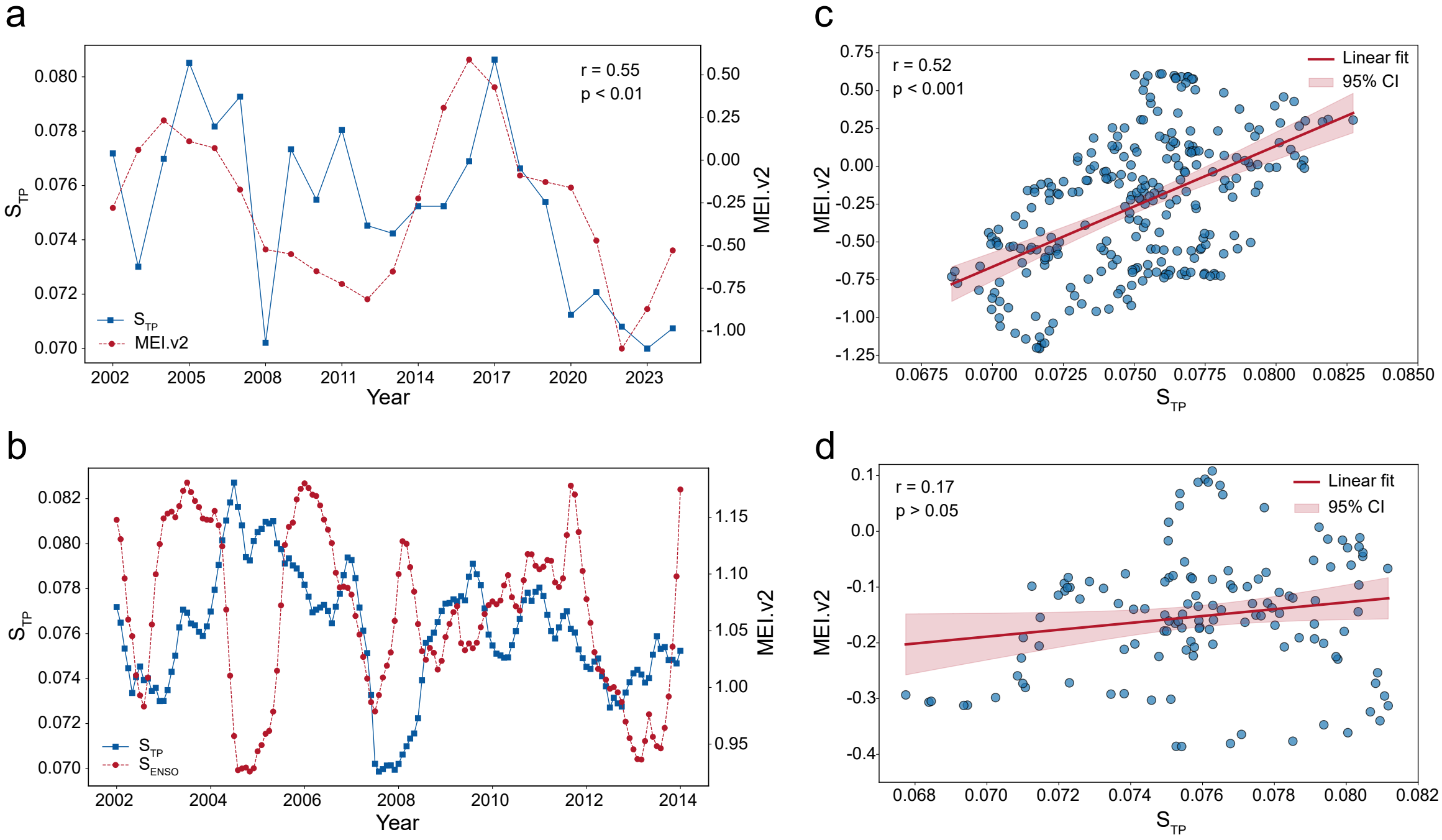


**Fig. S6 Relationship between $S_{\mathrm{TP}}$ and ENSO.** (**a**) Temporal evolution of $S_{\mathrm{TP}}$ (blue) and the ENSO index (MEI.v2, red). (**b**) Temporal evolution of $S_{\mathrm{TP}}$ (blue) and the $S_{\mathrm{ENSO}}$ (red) in CMIP6 historical simulations. (**c**) Scatterplot of $S_{\mathrm{TP}}$ versus the ENSO index (MEI.v2) during all periods. (**d**) Same as (**c**) but for neutral periods.

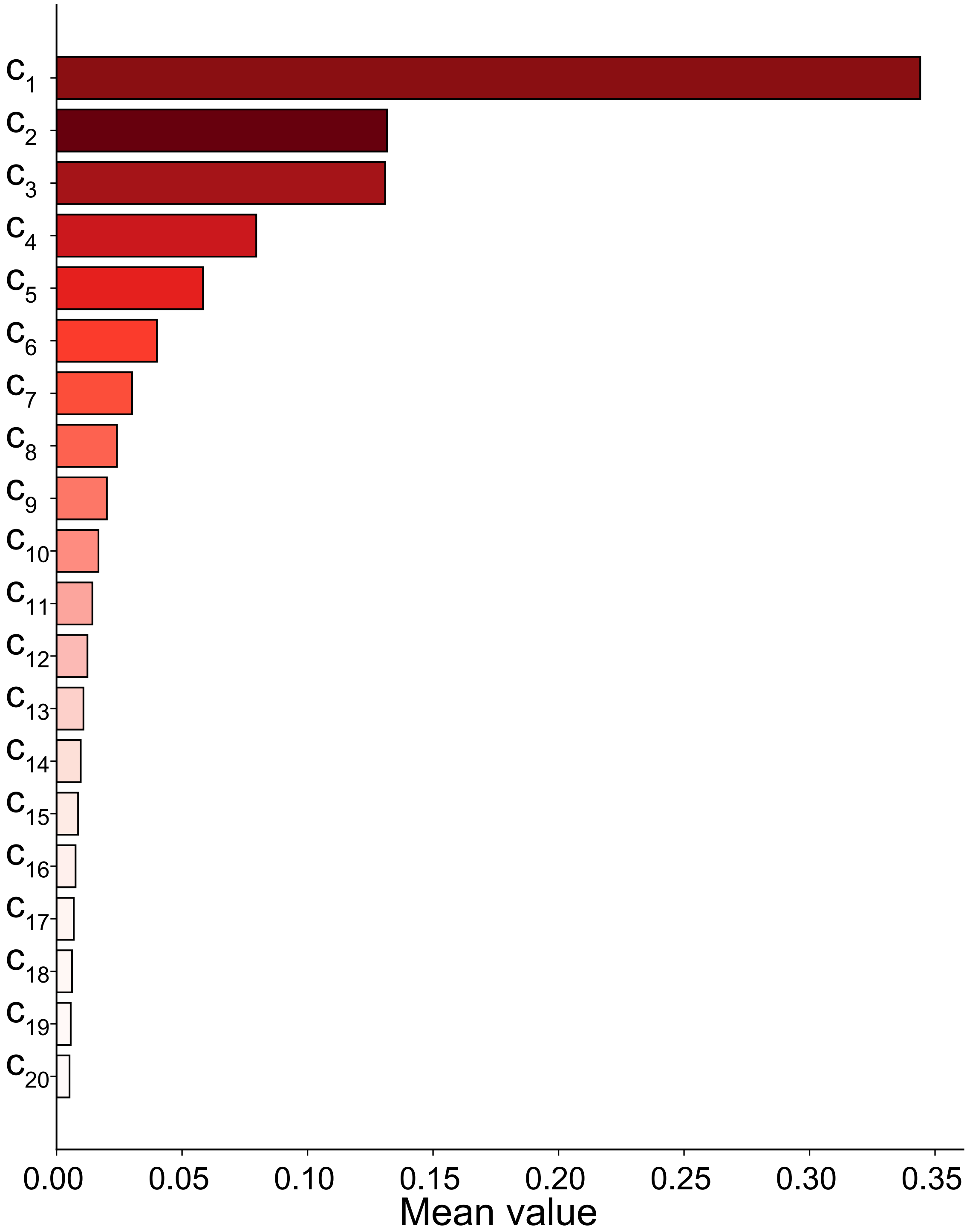


**Fig. S7 Mean contributions of the top twenty eigen microstates to $S_{\mathrm{TP}}$.**

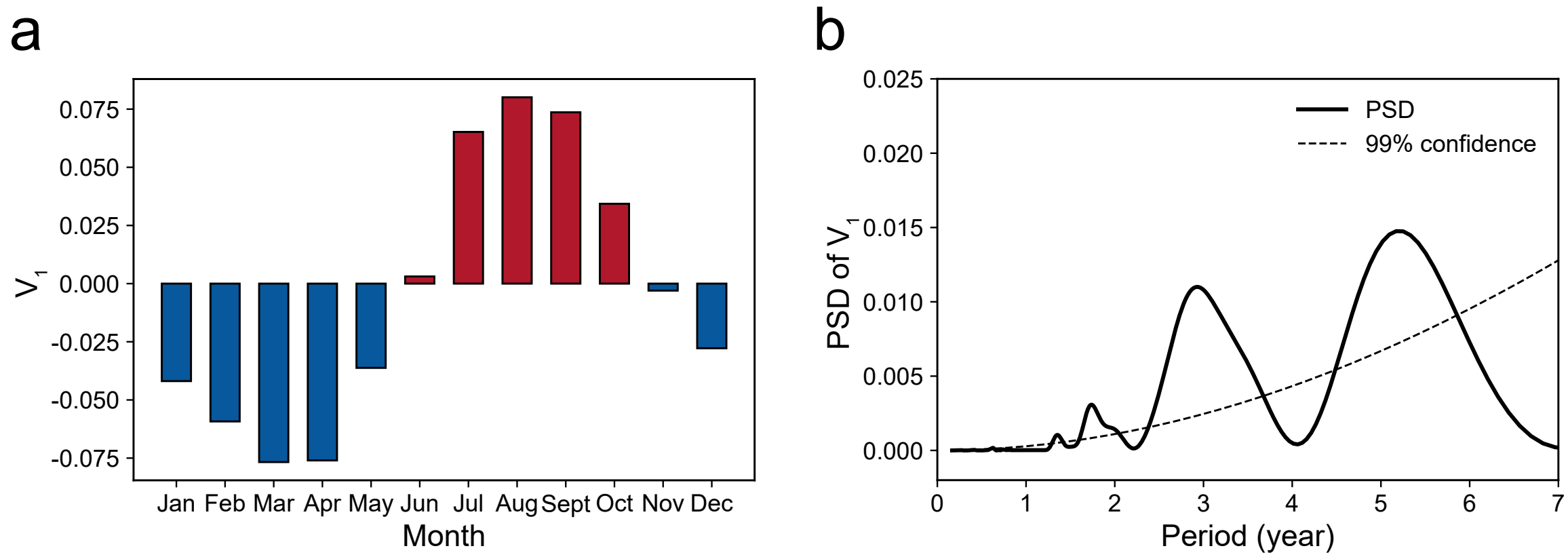


**Fig. S8 Monthly climatology and power spectrum density of the temporal evolution of soil moisture EM1.** (**a**) Monthly climatology of temporal evolution $V_1$. (**b**) Power spectral densities (PSD) of 12-month smoothed $V_1$ (solid line), representing interannual variability, and its 99% significance levels (dashed lines).

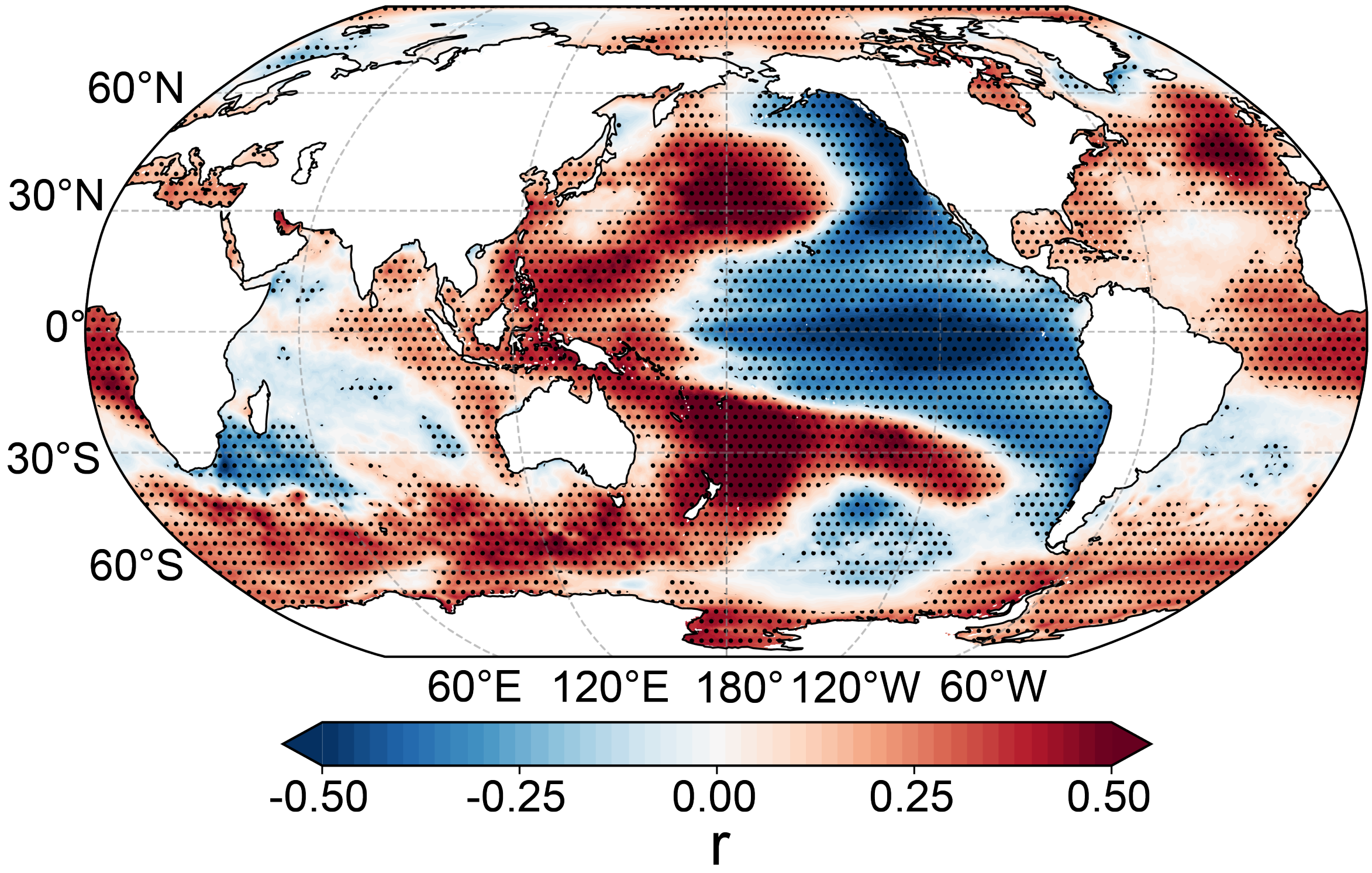


**Fig. S9 Spatial correlation maps between the temporal evolution of the soil moisture dipole mode ($V_1$) and global ocean surface air temperature, revealing the characteristic ENSO pattern over the tropical Pacific.**

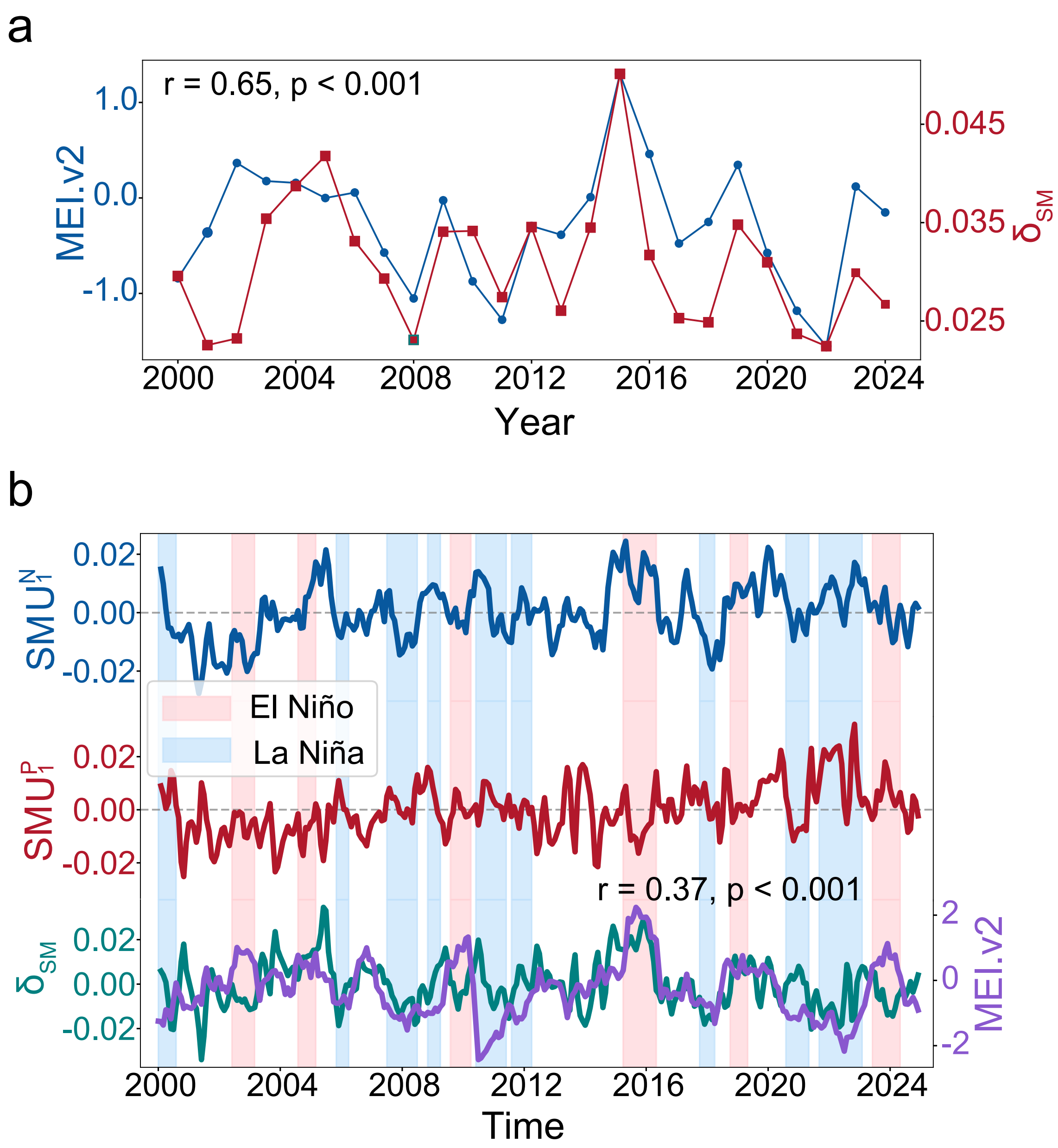


**Fig. S10 Relationship between ENSO and SM variability.** (**a**) Annual evolution of the ENSO index (blue) and SM difference (red) between positive and negative $U_1$ phase regions, showing a significant correlation of 0.65 ($p < 0.001$). (**b**) Monthly anomalies of SM in positive and negative $U_1$ phase regions. The green line shows their two-month running mean difference, and the purple line denotes the ENSO index, with a correlation of 0.37 ($p < 0.001$). Pink and blue shading indicate El Niño and La Niña events, respectively.

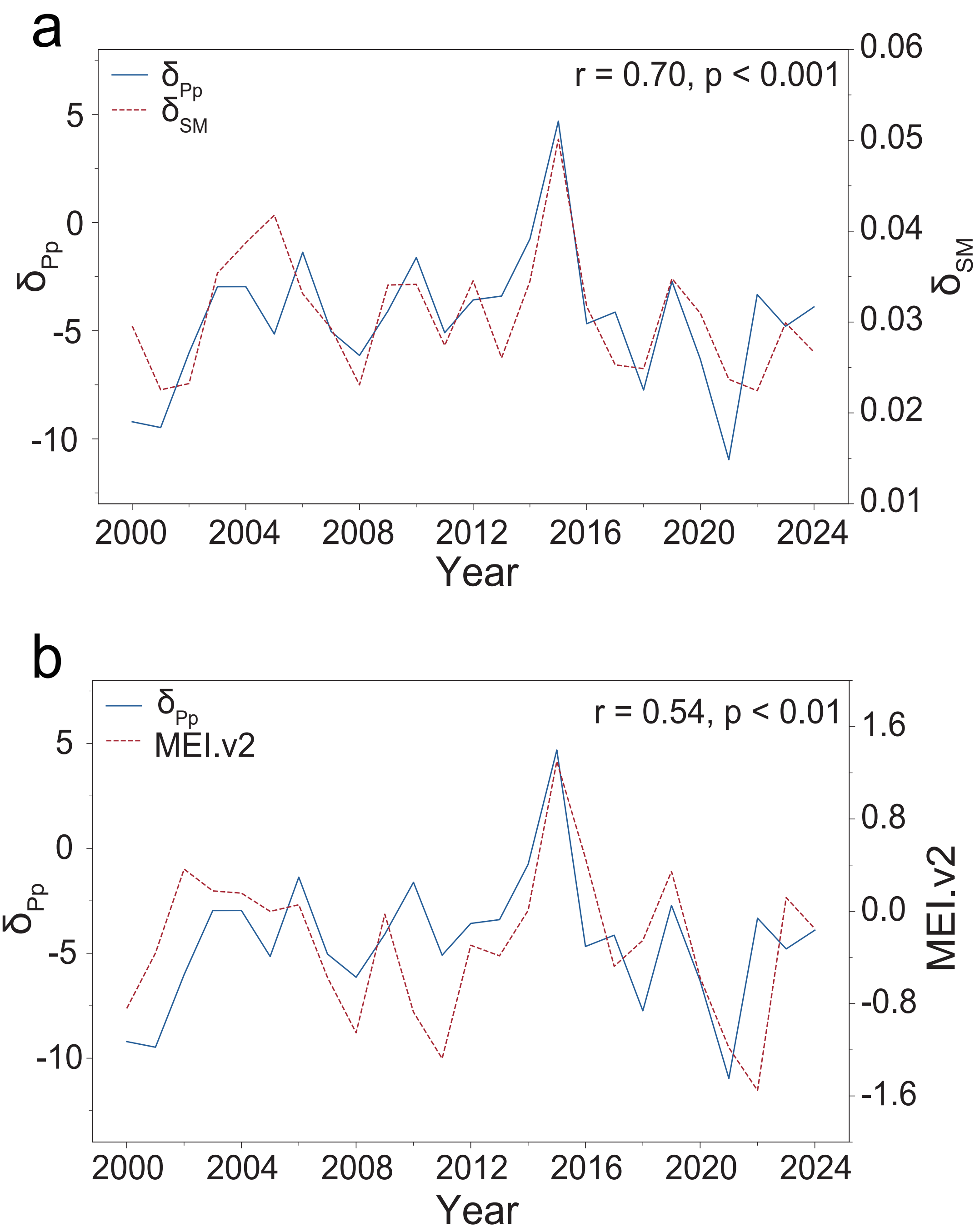


**Fig. S11 Annual Pp differences associated with SM differences and ENSO.** (**a**) Annual difference of Pp ($\delta_{\mathrm{Pp}}$, blue) and SM ($\delta_{\mathrm{SM}}$, red), defined as the yearly mean values in negative phase regions minus that in positive phase regions of SM $U_1$, showing a significant correlation($r = 0.70, p < 0.001$). (**b**) Annual evolution of Pp difference (blue) and the ENSO index (red), exhibiting a significant correlation($r = 0.54, p < 0.01$).

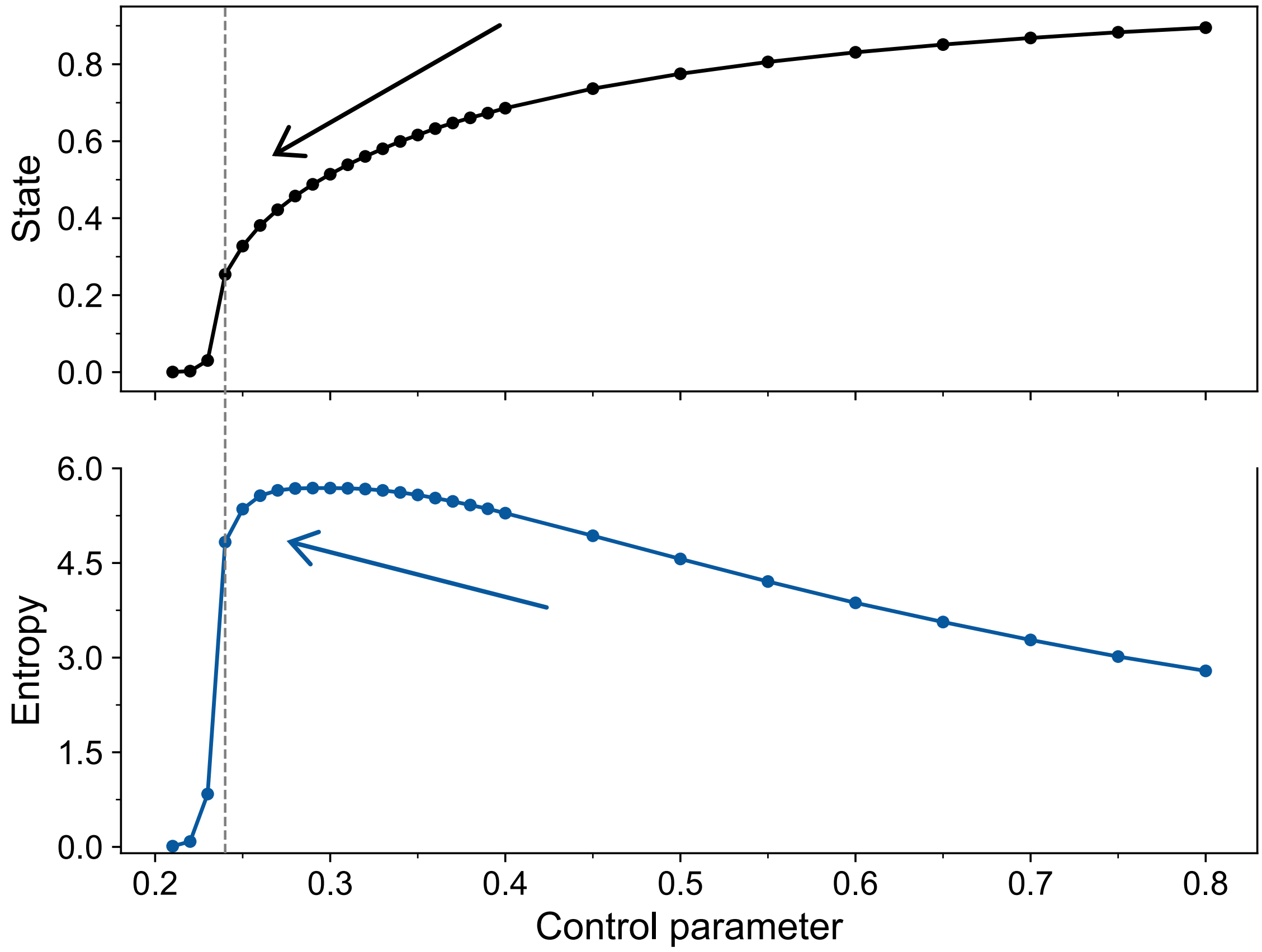


**Fig. S12 Early warning signal in the LFCA model.** The system state (black) declines as the control parameter weakens, approaching a critical threshold (grey dashed line). This transition is preceded by a rise in entropy (blue), serving as a precursor signal of the impending abrupt change.

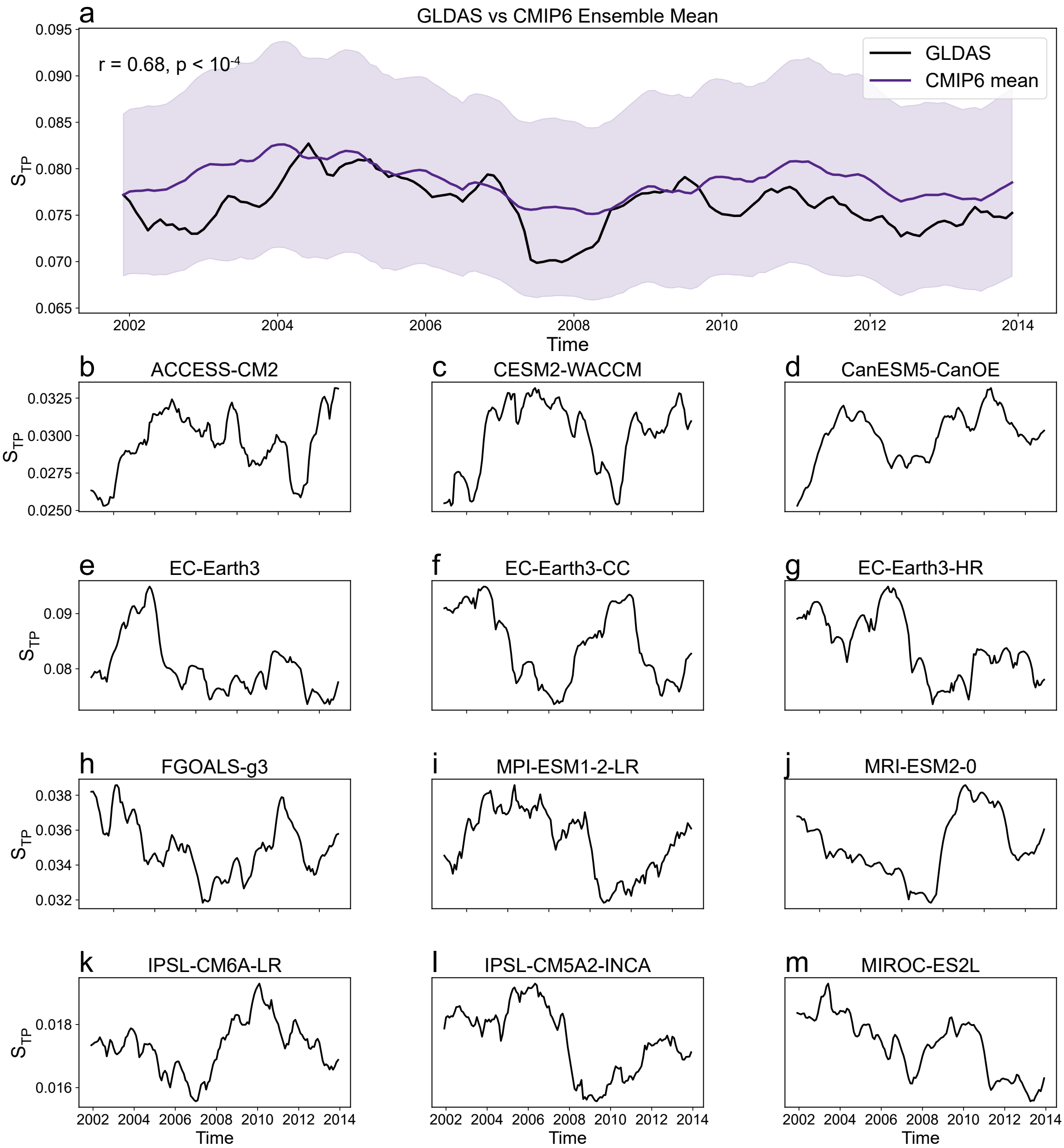


**Fig. S13 Evolution of $S_{\mathbf{TP}}$ from GLDAS-NOAH and CMIP6 historical experiments.** (**a**) GLDAS-NOAH and ensemble mean of 12 CMIP6 models, with shading indicating the standard error. (**b**)-(**m**) Results from 12 individual CMIP6 models.

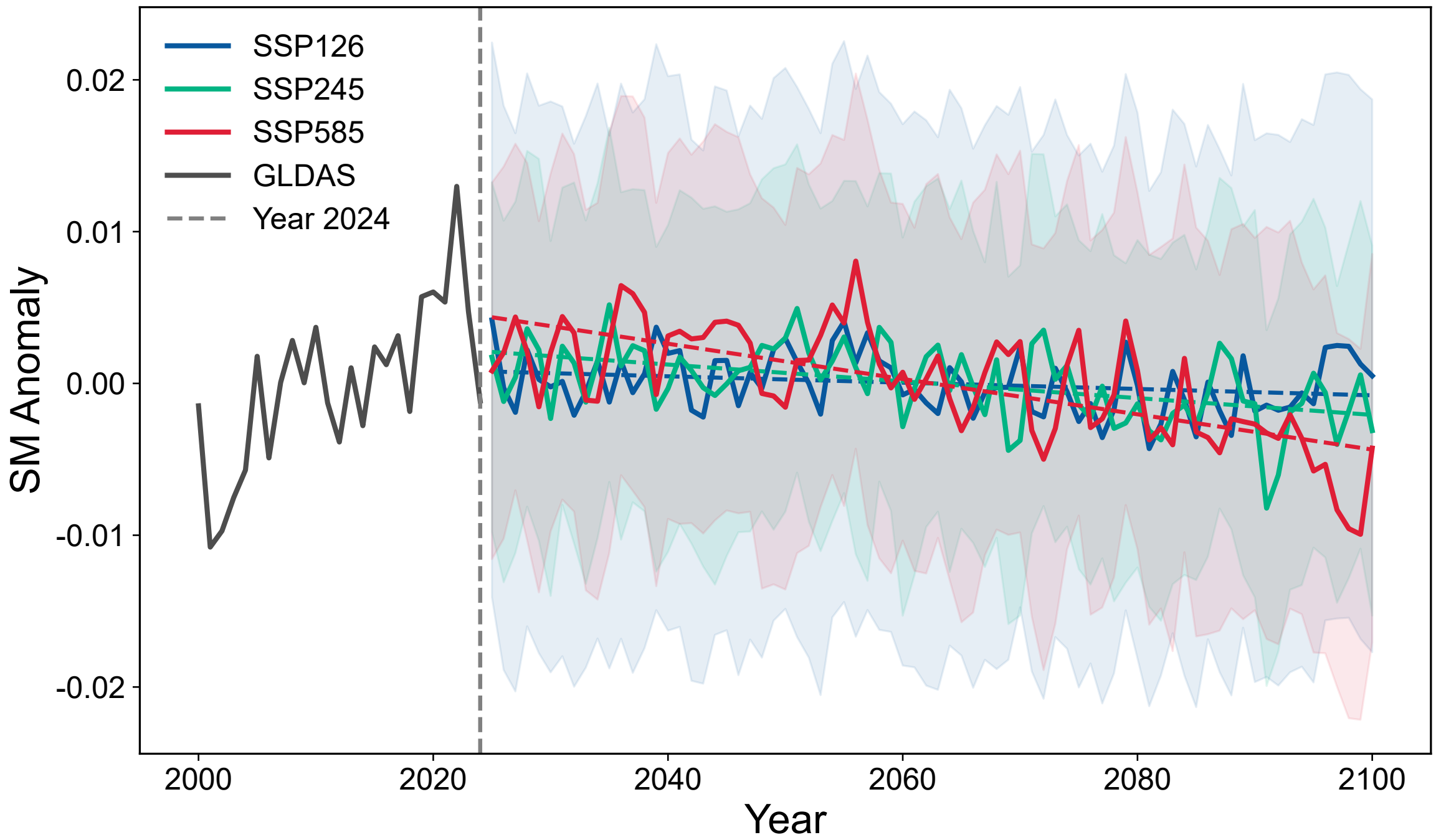


**Fig. S14 Annual SM anomalies from GLDAS-NOAH (2000–2024) and CMIP6 ensemble means (2025–2100) under SSP126 (blue), SSP245 (green), and SSP585 (red) scenarios.** Dashed lines denote linear trends, and shaded areas indicate the standard error. All scenarios exhibit significant decreasing trends ($p < 0.05$).

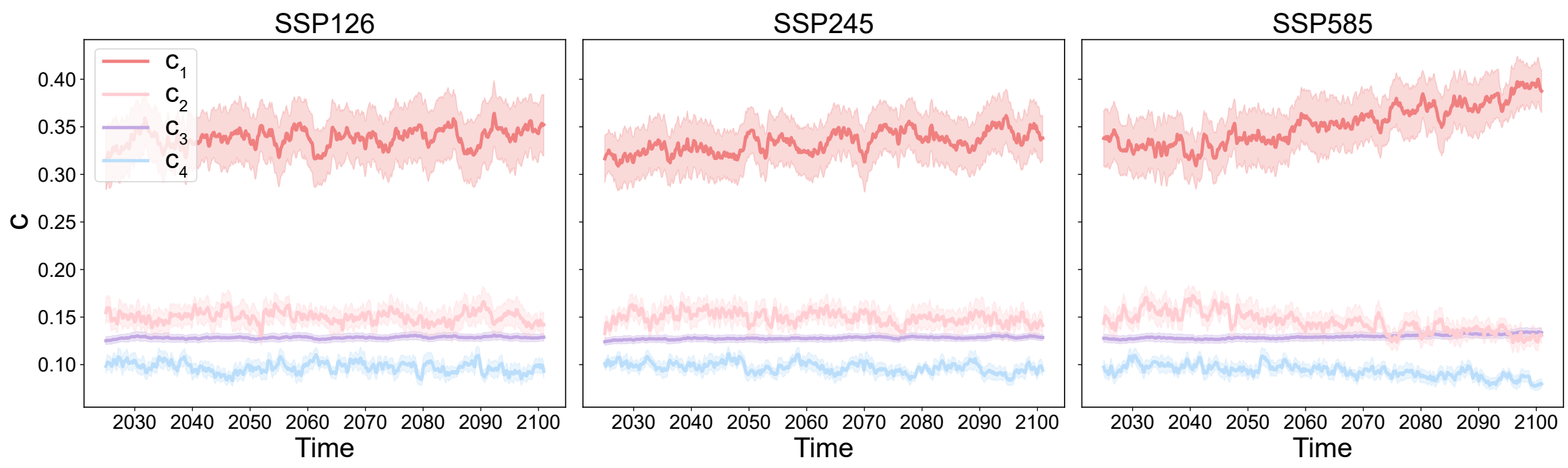


**Fig. S15 Temporal evolution of mean contributions of the four dominant eigen microstates to $S_{\mathrm{TP}}$ under SSP126, SSP245, and SSP585 scenarios, collectively explaining over 70% of the variability in $S_{\mathrm{TP}}$ under each scenario. Shaded areas indicate the standard error.**

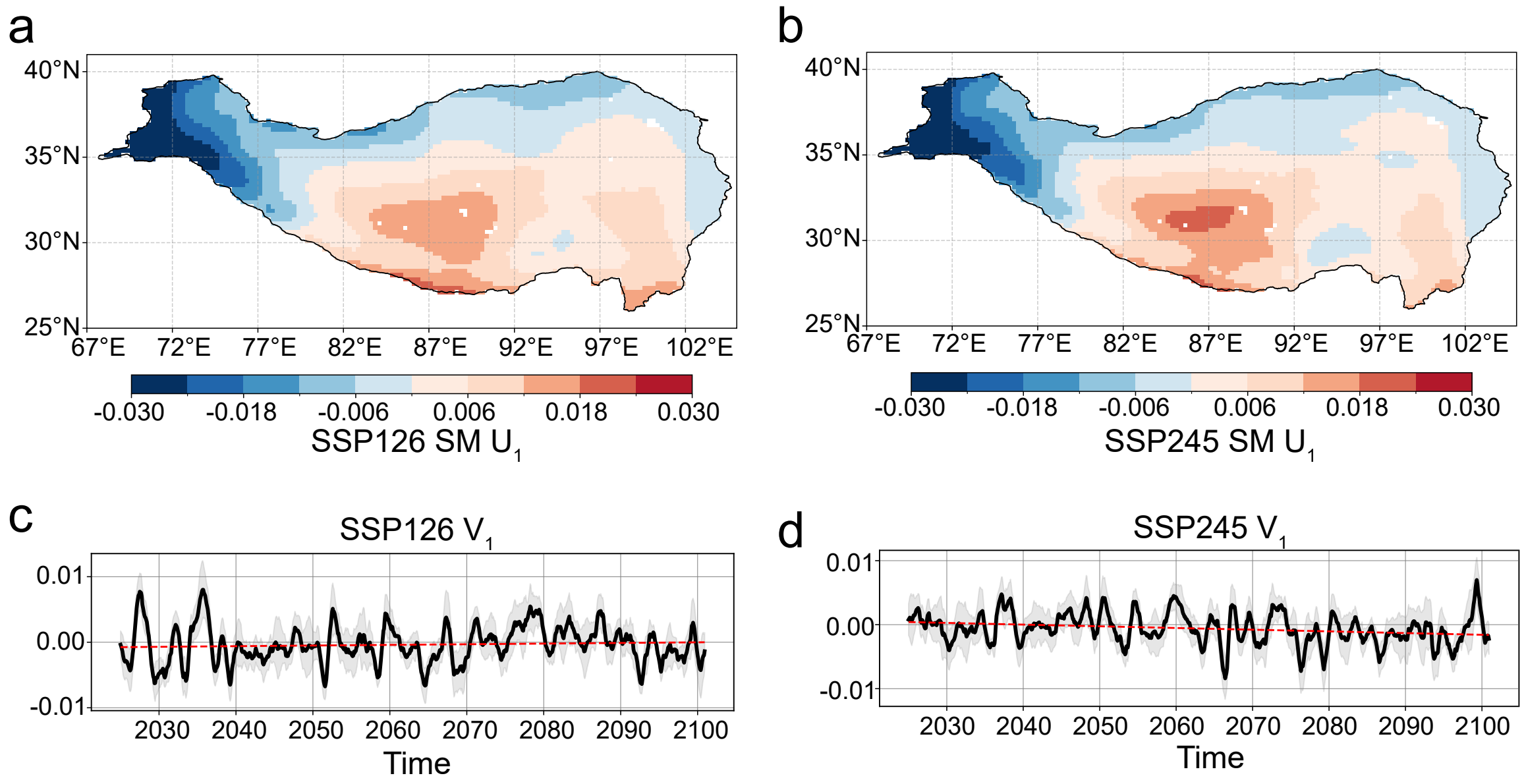


**Fig. S16 Ensemble-mean spatiotemporal patterns of the soil moisture EM1 under SSP126 and SSP245 scenarios. Red dashed lines denote linear trends, and shaded areas indicate the standard error.**

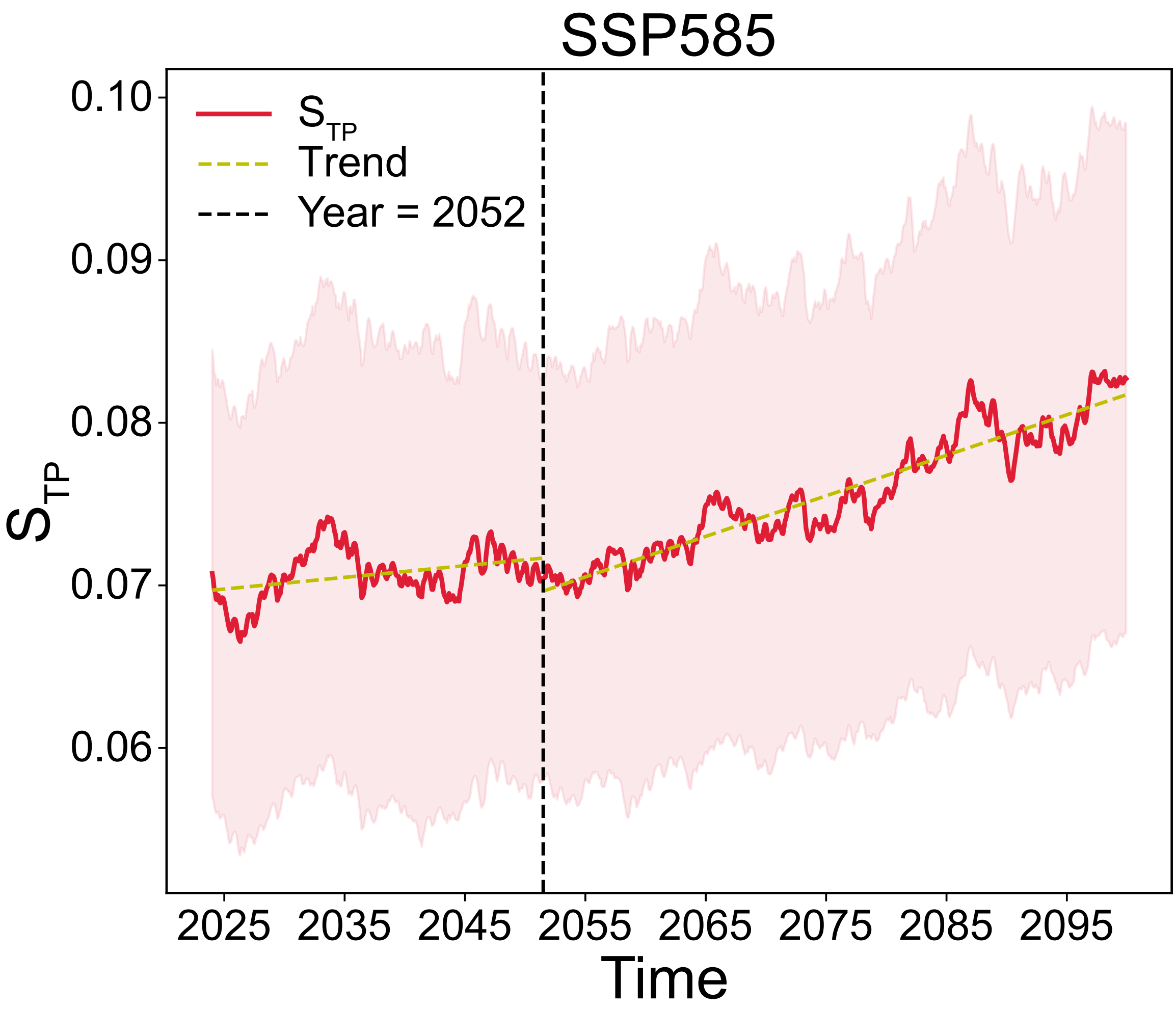


**Fig. S17** **Projected evolution of $S_{\mathrm{TP}}$ during 2025–2100 based on CMIP6 models under SSP585. A statistically significant breakpoint in 2052 (Welch's $t$-test at the 0.001 significance level) is marked by the black dashed line. Yellow dashed lines denote linear trends, and shaded areas indicate the standard error.**

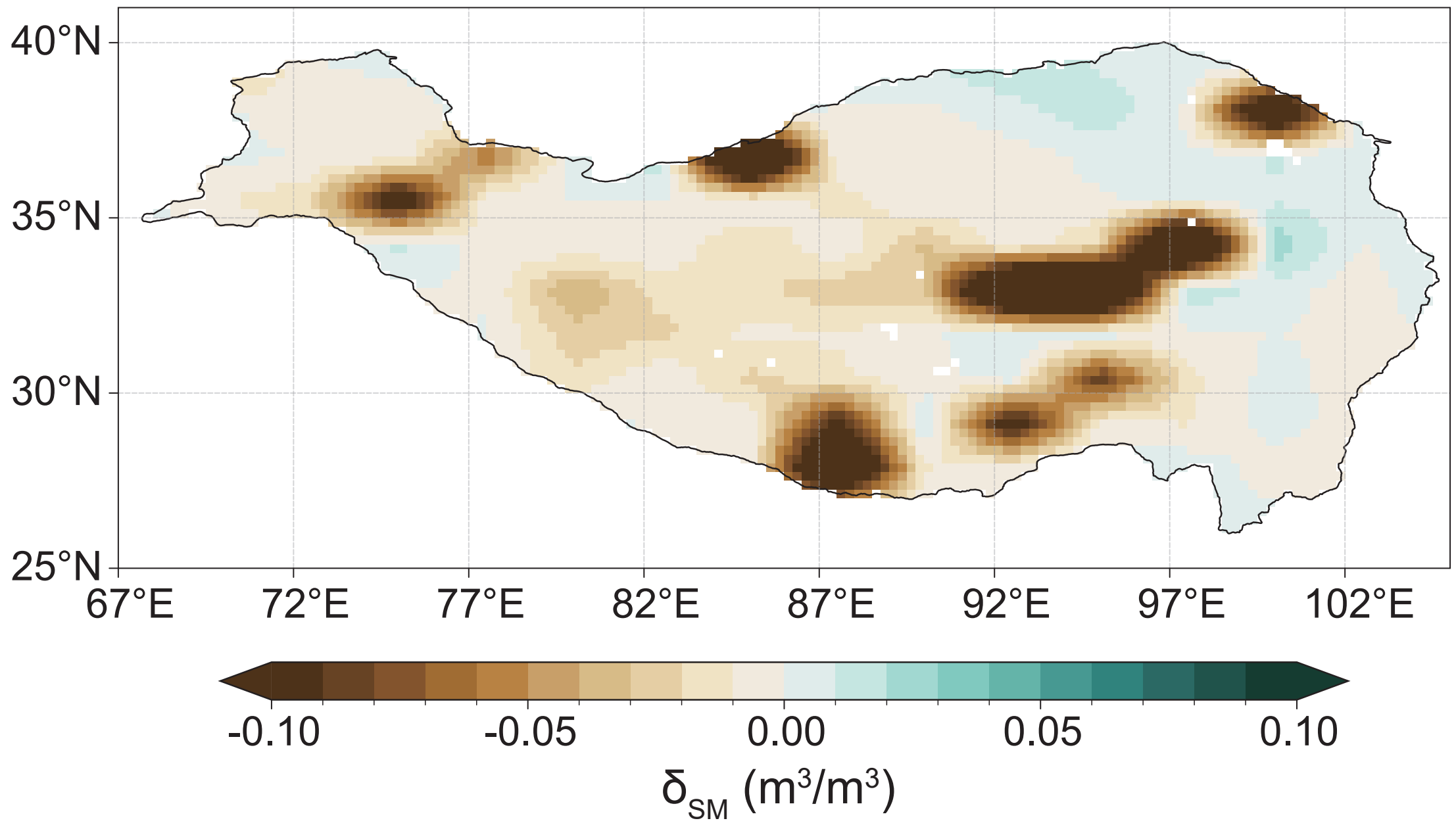


**Fig. S18 Spatial distribution of the mean SM anomaly difference between State II and State I simulated by the IPSL-CM6A-LR model under SSP585.**

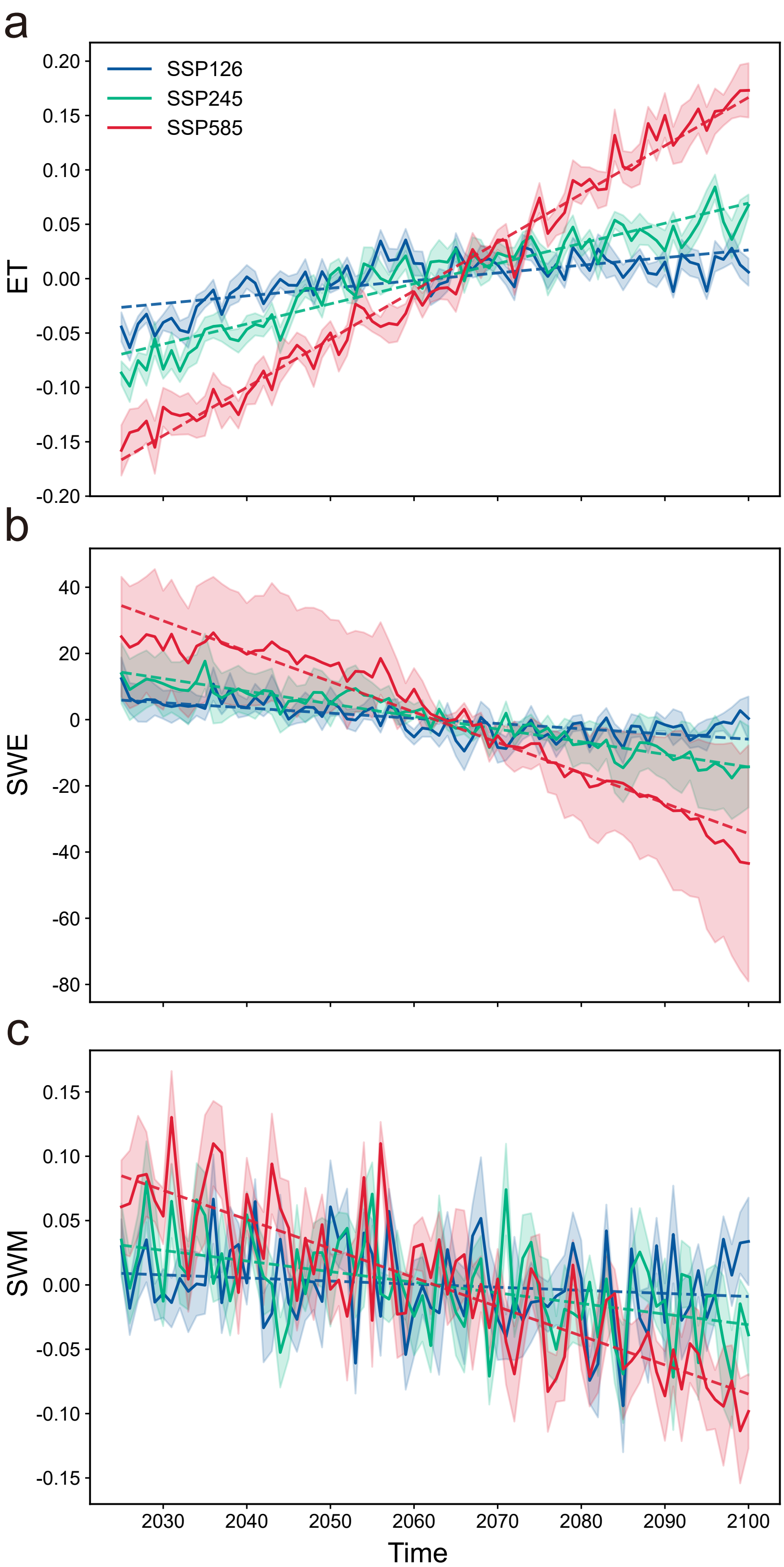


**Fig. S19 Annual mean anomalies of (a) total evaporation flux (ET), (b) surface snow water equivalent (SWE), and (c) surface snowmelt flux (SWM) based on the CMIP6 multi-model ensemble mean during 2025–2100 under SSP126 (blue), SSP245 (green), and SSP585 (red) scenarios.** Dashed lines denote linear trends, and shaded areas indicate the standard error. ET increases significantly while SWE decreases significantly across all scenarios ($p < 0.01$). SWM decreases significantly under SSP245 and SSP585.

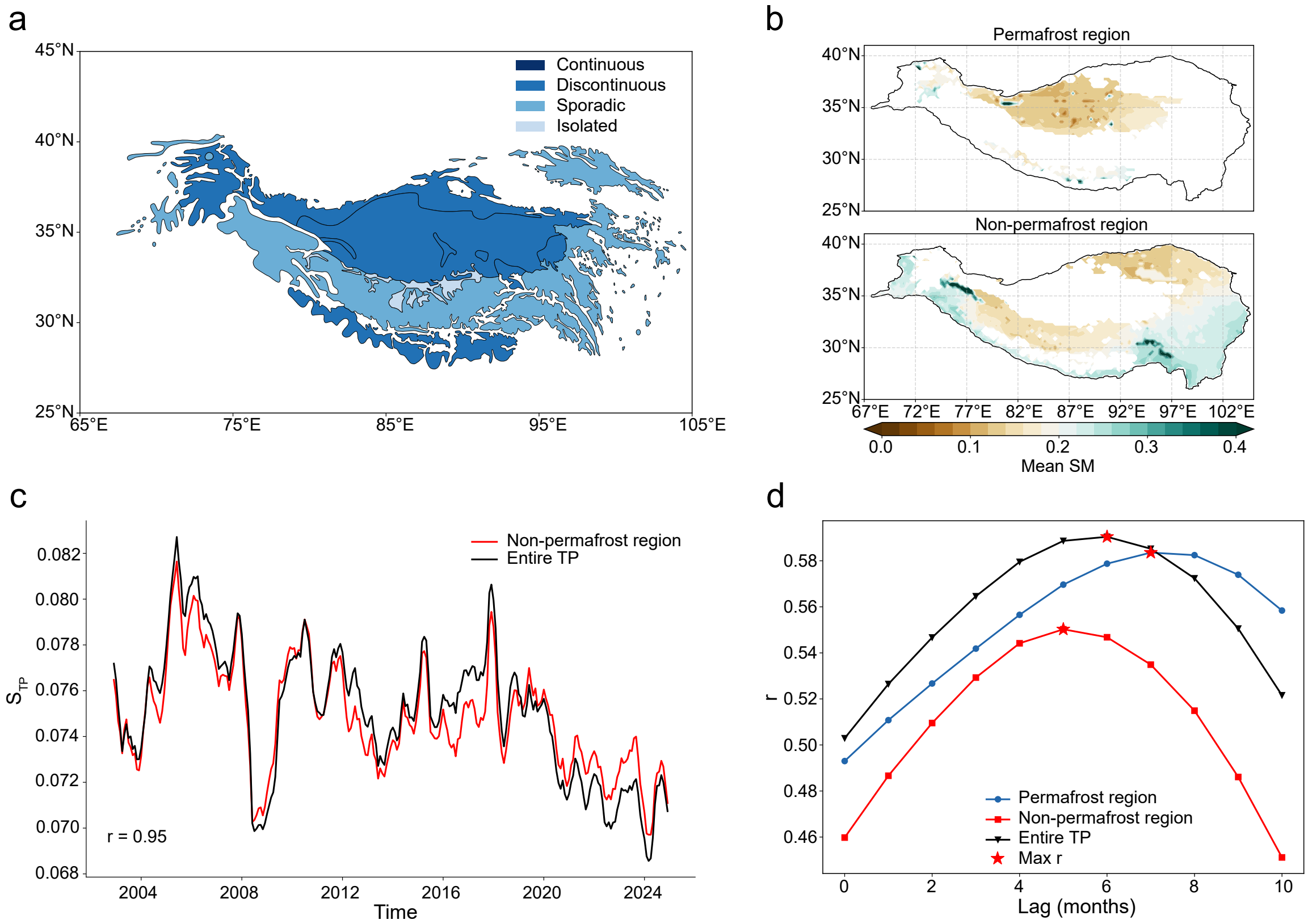


**Fig. S20 Sensitivity analysis of permafrost-related uncertainty in SM entropy over the TP.** Permafrost regions were identified using the Circum-Arctic Map of Permafrost and Ground-Ice Conditions (Version 2) provided by the National Snow and Ice Data Center [1]. (**a**) Spatial distribution of permafrost and non-permafrost regions over the TP. Continuous and discontinuous permafrost (permafrost extent $\geq$ 50%) are classified as permafrost regions, whereas sporadic and isolated permafrost (permafrost extent <50%) are classified as non-permafrost regions. (**b**) Mean SM distribution in permafrost and non-permafrost regions. (**c**) Temporal evolution of SM entropy over the all TP and non-permafrost regions. (**d**) Lagged correlations between ENSO entropy and SM entropy for the entire TP and non-permafrost regions

# Supplementary Tables

**Table S1 Summary of CMIP6 models and selection results.**

| Model | Experiment | Member | Selected | $R_T$ | $p$ | $R_F$ |
|---|---|---|---|---|---|---|
| ACCESS-CM2 | Historical, SSP126, SSP245, SSP585 | r1i1p1f1 | ✓ | 0.270 | 0.001 | 0.855 |
| CESM2-WACCM | Historical, SSP126, SSP245, SSP585 | r1i1p1f1 | ✓ | 0.166 | 0.046 | 0.949 |
| CanESM5-CanOE | Historical, SSP126, SSP245, SSP585 | r1i1p2f1 | ✓ | 0.431 | $< 0.001$ | 0.926 |
| EC-Earth3 | Historical, SSP126, SSP245, SSP585 | r1i1p1f1 | ✓ | 0.604 | $< 0.001$ | 0.910 |
| EC-Earth3-CC | Historical, SSP245, SSP585 | r1i1p1f1 | ✓ | 0.280 | 0.001 | 0.890 |
| EC-Earth3-HR | Historical, SSP245 | r1i1p1f1 | ✓ | 0.242 | 0.003 | 0.749 |
| FGOALS-g3 | Historical, SSP126, SSP245, SSP585 | r1i1p1f1 | ✓ | 0.187 | 0.024 | 0.807 |
| MIROC-ES2L | Historical, SSP126, SSP245, SSP585 | r1i1p1f2 | ✓ | 0.426 | $< 0.001$ | 0.888 |
| MPI-ESM1-2-LR | Historical, SSP126, SSP245, SSP585 | r1i1p1f1 | ✓ | 0.262 | 0.002 | 0.671 |
| MRI-ESM2-0 | Historical, SSP126, SSP245, SSP585 | r1i1p1f1 | ✓ | 0.168 | 0.044 | 0.859 |
| IPSL-CM6A-LR | Historical, SSP126, SSP245, SSP585 | r1i1p1f1 | ✓ | 0.0001 | 0.999 | 0.830 |
| IPSL-CM5A2-INCA | Historical, SSP126 | r1i1p1f1 | ✓ | 0.161 | 0.053 | 0.781 |
| UKESM1-0-LL | Historical, SSP126, SSP245, SSP585 | r1i1p1f2 | - | -0.040 | 0.631 | 0.905 |
| ACCESS-ESM1-5 | Historical, SSP126, SSP245, SSP585 | r1i1p1f1 | - | -0.023 | 0.786 | 0.447 |
| CAS-ESM2-0 | Historical, SSP245, SSP585 | r1i1p1f1 | - | -0.174 | 0.036 | 0.920 |
| CNRM-ESM2-1 | Historical, SSP126, SSP245, SSP585 | r1i1p1f2 | - | -0.044 | 0.597 | 0.633 |
| CanESM5 | Historical, SSP126, SSP245, SSP585 | r1i1p1f1 | - | -0.240 | 0.004 | 0.880 |
| CanESM5-1 | Historical, SSP126, SSP245, SSP585 | r1i1p1f1 | - | -0.255 | 0.002 | 0.802 |
| E3SM-1-0 | Historical, SSP585 | r1i1p1f1 | - | 0.035 | 0.675 | 0.782 |
| EC-Earth3-Veg-LR | Historical, SSP126, SSP245, SSP585 | r1i1p1f1 | - | -0.557 | $< 0.001$ | 0.834 |
| GISS-E2-1-H | Historical, SSP126, SSP245, SSP585 | r1i1p1f2 | - | -0.090 | 0.280 | 0.674 |
| GISS-E2-2-G | Historical, SSP126, SSP245, SSP585 | r1i1p3f1 | - | -0.173 | 0.038 | 0.847 |
| MIROC6 | Historical, SSP126, SSP245, SSP585 | r1i1p1f1 | - | -0.190 | 0.022 | 0.816 |
| CESM2 | Historical, SSP126, SSP245, SSP585 | r4i1p1f1 | - | -0.065 | 0.437 | 0.832 |
| GFDL-CM4 | Historical, SSP245, SSP585 | r1i1p1f1 | - | -0.035 | 0.675 | 0.893 |

**Table S2 Evaluation metrics for assessing the applicability of SM datasets over the TP.**

| Metric | Measurement | Definition |
|---|---|---|
| $R_T$ | Temporal correlation | Eq. (S1) |
| RMSE | Magnitude of deviations | Eq. (S2) |
| Bias | Systematic offset | Eq. (S3) |
| $R_F$ | Spectral correlation | Eq. (S4) |
| $R_{ACF}$ | Autocorrelation similarity | Eq. (S5) |

$$R_T = \frac{\sum_{n=1}^{N} \left(\mathrm{SM}_{\mathrm{prod},n} - \overline{\mathrm{SM}}_{\mathrm{prod}}\right) \left(\mathrm{SM}_{\mathrm{obs},n} - \overline{\mathrm{SM}}_{\mathrm{obs}}\right)}{\sqrt{\sum_{n=1}^{N} \left(\mathrm{SM}_{\mathrm{prod},n} - \overline{\mathrm{SM}}_{\mathrm{prod}}\right)^2} \sqrt{\sum_{n=1}^{N} \left(\mathrm{SM}_{\mathrm{obs},n} - \overline{\mathrm{SM}}_{\mathrm{obs}}\right)^2}}, \tag{S1}$$

where $\mathrm{SM}_{\mathrm{prod},n}$ and $\mathrm{SM}_{\mathrm{obs},n}$ are the gridded and in-situ SM at time $n$; $\overline{\mathrm{SM}}_{\mathrm{prod}}$ and $\overline{\mathrm{SM}}_{\mathrm{obs}}$ are their means; and $N$ is the number of samples.

$$\mathrm{RMSE} = \sqrt{\frac{1}{N} \sum_{n=1}^{N} \left(\mathrm{SM}_{\mathrm{prod},n} - \mathrm{SM}_{\mathrm{obs},n}\right)^2}. \tag{S2}$$

$$\mathrm{Bias} = \frac{1}{N} \sum_{n=1}^{N} \left(\mathrm{SM}_{\mathrm{prod},n} - \mathrm{SM}_{\mathrm{obs},n}\right). \tag{S3}$$

$$R_F = \frac{\sum_{i=1}^{n} \left(S_{\mathrm{prod},i} - \overline{S}_{\mathrm{prod}}\right) \left(S_{\mathrm{obs},i} - \overline{S}_{\mathrm{obs}}\right)}{\sqrt{\sum_{i=1}^{n} \left(S_{\mathrm{prod},i} - \overline{S}_{\mathrm{prod}}\right)^2} \sqrt{\sum_{i=1}^{n} \left(S_{\mathrm{obs},i} - \overline{S}_{\mathrm{obs}}\right)^2}}, \tag{S4}$$

where $S_{\mathrm{prod},i}$ and $S_{\mathrm{obs},i}$ denote spectral amplitudes at frequency bin $i$, and $n$ is the total number of bins.

$$R_{ACF} = \frac{\sum_{k=1}^{K} \left(ACF_{\mathrm{prod},k} - \overline{ACF}_{\mathrm{prod}}\right) \left(ACF_{\mathrm{obs},k} - \overline{ACF}_{\mathrm{obs}}\right)}{\sqrt{\sum_{k=1}^{K} \left(ACF_{\mathrm{prod},k} - \overline{ACF}_{\mathrm{prod}}\right)^2} \sqrt{\sum_{k=1}^{K} \left(ACF_{\mathrm{obs},k} - \overline{ACF}_{\mathrm{obs}}\right)^2}}, \tag{S5}$$

where $ACF_{\mathrm{prod},k}$ and $ACF_{\mathrm{obs},k}$ are lag-$k$ autocorrelation coefficients following the definition in Eq. (S6). In this study, $K = 10$ was set to capture the short- to medium-term autocorrelation characteristics of the time series.

$$ACF_k = \frac{\mathrm{Cov}(X_t, X_{t+k})}{\sigma_{X_t}\, \sigma_{X_{t+k}}}, \tag{S6}$$

where $\mathrm{Cov}(X_t, X_{t+k})$ is the covariance between a time series and its lag-$k$ version.

**Table S3 Values of the simulation parameters for the Local Facilitation Cellular Automaton (LFCA) model.**

| Parameter | Value | Meaning |
|---|---|---|
| $m$ | 0.1 | Mortality rate |
| $d$ | 0.1 | Degradation rate |
| $c$ | 0.1 | Competition strength |
| $r$ | 0.01 | Spontaneous regeneration rate |
| $f$ | 0.9 | Local facilitation coefficient |
| $b$ | 0–1 | Wetness |
| $\delta$ | 0.1 | Seed dispersal rate |
| $dt$ | 0.5 | Time step |

# Supplementary Note S1 CMIP6 Model Selection

## S1.1 Phase-scrambling null distribution

CMIP6 historical simulations are uninitialized free-running experiments [2, 3]. Their internal variability chronology (e.g., timing of ENSO events) is independent of the observed record. To quantify the resulting uncertainty in the Pearson temporal correlation $R_T$, we generated 50,000 phase-scrambled surrogates of the GLDAS $S_{\mathrm{TP}}$ series by randomising the Fourier phases while preserving the power spectral density. As shown in Supplementary Fig. 21, for $N = 145$ monthly steps with the observed autocorrelation structure, this yields $\sigma(R_T) \approx 0.35$ and 5th/95th percentiles of $-0.56/+0.56$. A model with correct spectral structure but random ENSO phase will produce $|R_T| < 0.56$ in 90% of realisations, confirming that $R_T$ alone is an unreliable selection metric at this record length.

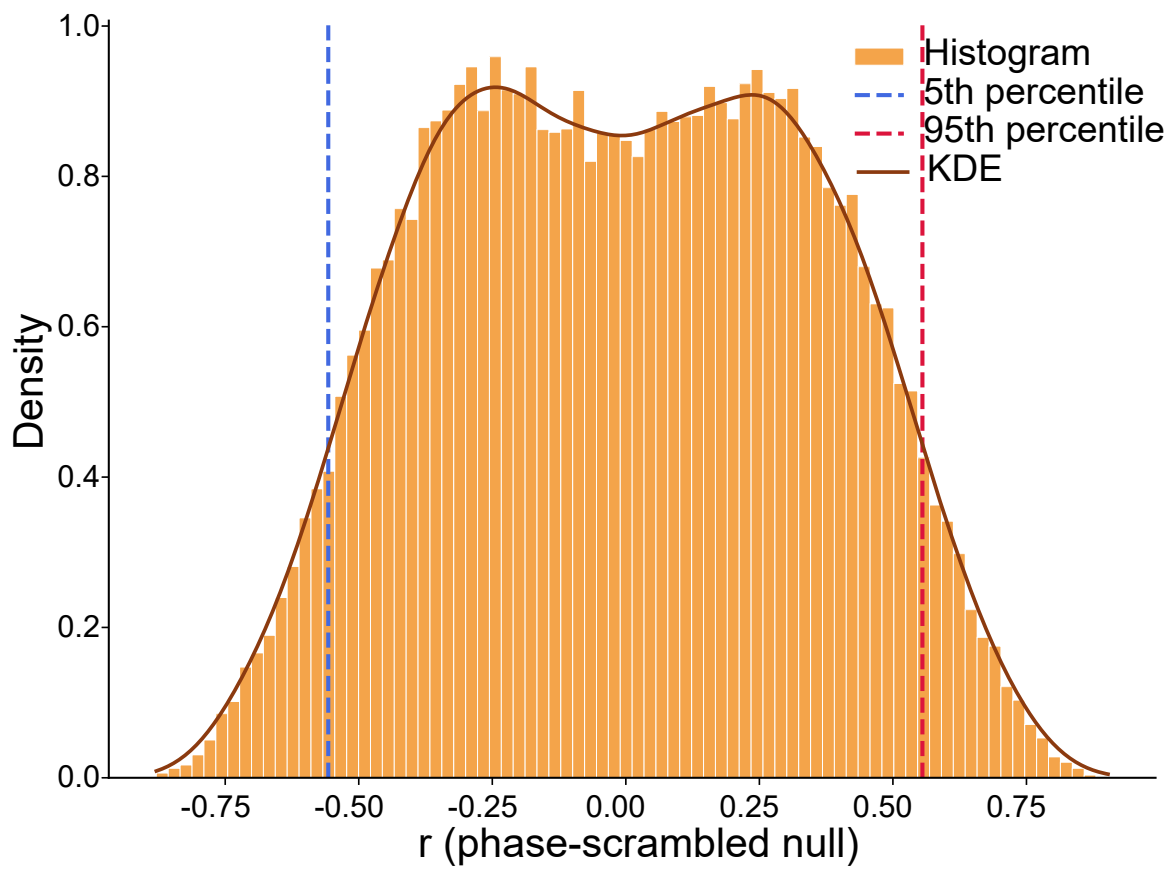


**Fig. S21 Null distribution of $R_T$ derived from phase-scrambled GLDAS surrogates.**

## S1.2 Spectral correlation $R_F$

The spectral correlation $R_F$ is defined as the Pearson correlation coefficient between the spectral amplitudes of the two standardised (zero-mean, unit-variance) $S_{\mathrm{TP}}$ series. $R_F$ measures whether the model reproduces the dominant variability periods of $S_{\mathrm{TP}}$ irrespective of temporal phase.

## S1.3 Threshold justification

The thresholds used in the two-tier model selection framework are justified as follows:

- **Tier 1 threshold ($p < 0.10$):** Standard one-tailed significance level used to identify models with statistically significant positive temporal agreement.
- **Tier 2 thresholds ($|R_T| < 0.01$ and $R_F > 0.80$):** Identify models with effectively zero temporal correlation but strong spectral consistency relative to GLDAS, ensuring retention of models that reproduce the observed frequency structure despite phase mismatch.

## S1.4 Sensitivity of results to threshold choice

**Table S4 Sensitivity of model selection results to threshold choice.**

| Configuration | Tier 1 | Tier 2 | $n$ | hist $R_T$ |
|---|---|---|---|---|
| Main (used in paper) | $R_T > 0,\ p < 0.10$ | $\lvert R_T\rvert < 0.01,\ R_F > 0.80$ | 12 | 0.684 |
| Sens-1 (relaxed Tier 2: $\lvert R_T\rvert < 0.10$) | $R_T > 0,\ p < 0.10$ | $\lvert R_T\rvert < 0.10,\ R_F > 0.80$ | 15 | 0.655 |
| Sens-2 (strict Tier 1: $p < 0.01$) | $R_T > 0,\ p < 0.01$ | $\lvert R_T\rvert < 0.01,\ R_F > 0.80$ | 8 | 0.679 |

To verify robustness, the analysis was repeated under two alternative threshold combinations, as summarized in Table S4.

Here, $n$ denotes the number of models retained under each threshold configuration, and hist $R_T$ denotes the Pearson correlation between the historical ensemble-mean $S_{\mathrm{TP}}$ of each alternative model ensemble and that of GLDAS. All historical correlations exceed 0.65, indicating that the model selection results are insensitive to the specific threshold choices. Moreover, projected changes in the TP soil moisture system based on CMIP6 models selected under different threshold configurations are highly consistent with the results shown in Fig. 5, including the projected increase in $S_{\mathrm{TP}}$, the dominant soil moisture dipole pattern, and the breakpoint around 2052 in the temporal evolution of $V_1$ and $\delta_{\mathrm{SM}}$ (Supplementary Fig. 22).

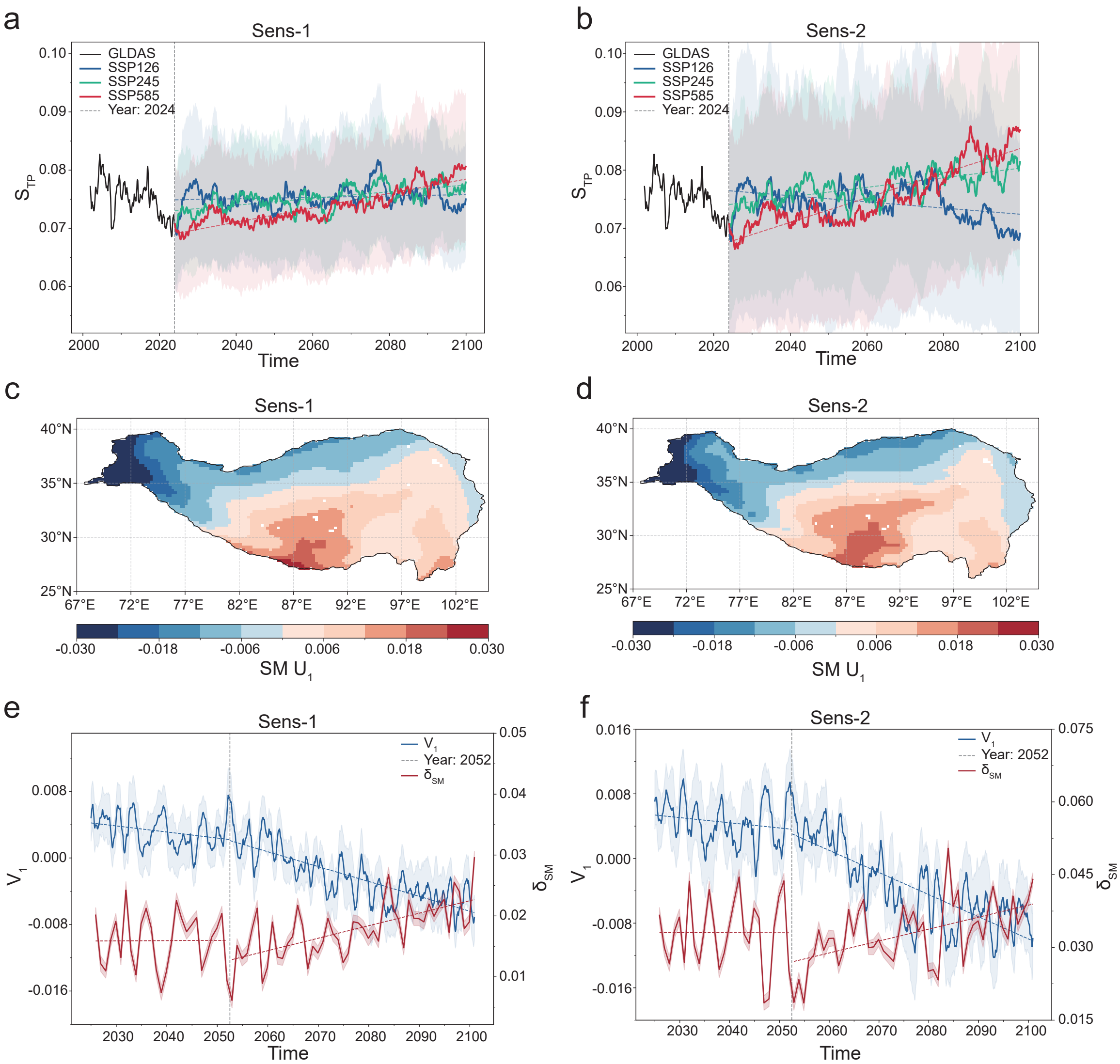


**Fig. S22 Robustness of projected changes in the TP soil moisture system to the model selection threshold.** (**a**) and (**b**) Same analysis as Fig. 5a in the main text, but from CMIP6 models selected under Sens-1 and Sens-2, respectively. The high emission scenarios exhibit consistently significant upward trends ($p < 0.001$), indicating a transition toward a more disordered and unstable regime. (**c**) and (**d**) Same analysis as Fig. 5b in the main text, showing the same dipole mode. (**e**) and (**f**) Same analysis as Fig. 5c in the main text. A common breakpoint is identified in 2052 (grey dashed line) by segmented linear regression in both sensitivity experiments. After the breakpoint, $V_1$ exhibits a significant downward trend ($p < 0.001$), with a slope more than twice that before the breakpoint. In contrast, $\delta_{\mathrm{SM}}$ shows no significant trend before the breakpoint but increases significantly thereafter ($p < 0.001$).

# Supplementary Note S2 Eigen Microstates Theory

In a complex system composed of $N$ spatial units (or agents), each characterized by a state variable $X_i(t)$ at discrete time $t = 1, 2, 3, \ldots, M$. The system's microstate at each time step is defined as the vector $\boldsymbol{X}(t) = [X_1(t), X_2(t), \ldots, X_N(t)]^\top$. An ensemble of $M$ such microstates constructs a data matrix $\boldsymbol{A} \in \mathbb{R}^{N\times M}$, where each element is normalized by a constant $C_0$ such that:

$$\boldsymbol{A}_{it} = \frac{X_i(t)}{\sqrt{C_0}}, \text{ with } C_0 = \sum_{t=1}^{M}\sum_{i=1}^{N} X_i^2(t), \tag{1}$$

ensuring that $\mathrm{Tr}(\boldsymbol{A}\boldsymbol{A}^\top) = 1$. For our analysis, we define $X_i(t) = \sqrt{Y_i(t)}$, where $Y_i(t)$ is the original value of the SM or Pp at location $i$ and time $t$, so that $C_0$ corresponds to the total mass (i.e., integrated signal) across all space and time.

Further, the temporal correlation matrix $\boldsymbol{C}$ and spatial correlation matrix $\boldsymbol{K}$ can be defined by the matrix $\boldsymbol{A}$ as

$$\boldsymbol{C} = C_0\, \boldsymbol{A}^\top \cdot \boldsymbol{A},$$
$$\boldsymbol{K} = C_0\, \boldsymbol{A} \cdot \boldsymbol{A}^\top,$$

The correlation matrix $\boldsymbol{C}$ has $M$ eigenvectors $\boldsymbol{V}_J$ for $J = 1, 2, \ldots, M$, which can form a $M \times M$ unitary matrix $\boldsymbol{V}$. The correlation matrix $\boldsymbol{K}$ has $N$ eigenvectors $\boldsymbol{U}_I$ for $I = 1, 2, \ldots, N$, which can form an $N \times N$ unitary matrix $\boldsymbol{U}$. According to singular value decomposition (SVD), the ensemble matrix $\boldsymbol{A}$ can be factorized as

$$\boldsymbol{A} = \boldsymbol{U} \cdot \boldsymbol{\Sigma} \cdot \boldsymbol{V}^\top, \tag{2}$$

where $\boldsymbol{\Sigma}$ is an $N \times M$ diagonal matrix with elements $\Sigma_{IJ} = \begin{cases} \sigma_I, & \text{for } I = J \le r, \\ 0, & \text{otherwise} \end{cases}$, and $r = \min(N, M)$. We can further rewrite the matrix $\boldsymbol{A}$ as

$$\boldsymbol{A} = \sum_{I=1}^{r} \sigma_I \boldsymbol{A}_I^e = \sum_{I=1}^{r} \sigma_I \boldsymbol{U}_I \otimes \boldsymbol{V}_I,$$

where $\otimes$ denotes the outer product. We refer to the ensemble defined by $\boldsymbol{A}_I^e$ as the eigen ensemble of the system. As $\sum_I \sigma_I^2 = 1$, $\sigma_I^2$ can be interpreted as the probability of the eigen ensemble $\boldsymbol{A}_I^e$ in the statistical ensemble $\boldsymbol{A}$. In conclusion, the statistical ensemble of the system can be determined through a linear superposition of eigen ensemble $\boldsymbol{A}_I^e$ weighted by their probabilities $\sigma_I^2$. From this statistical ensemble, we can obtain not only the spatial pattern of the eigen microstate $\boldsymbol{U}_I$, but also its temporal evolution $\boldsymbol{V}_I$.

# Supplementary Note S3 Rationale for moving-window selection

The window lengths for each entropy or correlation analysis were not selected independently but followed a common criterion: for each variable, we chose the shortest window that provides sufficient samples to ensure stable estimates while remaining as close as possible to the characteristic timescale of the process being resolved. The resulting differences across variables arise for two reasons: (i) the variables have different temporal resolutions (monthly soil moisture versus daily temperature), and (ii) the variables represent processes operating on different timescales, ranging from ENSO-related interannual variability to the bi-monthly MEI.v2 index. Sensitivity analyses further confirm that the main conclusions are insensitive to reasonable variations in window length.

## S3.1 Three-year window for $S_{\mathrm{TP}}$

The 3-year moving window used to calculate soil moisture entropy ($S_{\mathrm{TP}}$) was selected based on both statistical and physical considerations. From a statistical perspective, because $S_{\mathrm{TP}}$ is derived from monthly data, a 3-year window provides a sufficient sample size for robust estimation of the underlying spatiotemporal dependence structure; substantially shorter windows may introduce sampling instability into the entropy estimate.

From a physical perspective, a 3-year window is broadly consistent with the characteristic timescale of ENSO variability (typically 2–7 years), allowing the associated interannual soil moisture signal to be resolved without blending multiple ENSO events within a longer averaging window. This choice is further supported by the temporal evolution of the ENSO-related soil moisture dipole mode ($V_1$), which was identified from the full study-period dataset and exhibits dominant periodicities of approximately 2–3.8 years.

To further evaluate the sensitivity of this choice, we repeated the analysis using alternative window lengths of 2 and 4 years. The resulting $S_{\mathrm{TP}}$ time series remained highly consistent with the original 3-year estimate, with Pearson correlation coefficients exceeding 0.7 in both cases (Supplementary Fig. 23a). In addition, the maximum lag correlations between $S_{\mathrm{TP}}$ and $S_{\mathrm{ENSO}}$ remained above 0.5 for both alternative windows (Supplementary Fig. 23b), confirming that the main conclusions are robust to the choice of window length. However, the 2-year window yields a more fluctuating $S_{\mathrm{TP}}$ time series, whereas the 4-year window produces a noticeably smoother evolution. Therefore, the 3-year window provides a balanced compromise between preserving temporal variability and reducing estimation noise.

## S3.2 One-year window for $S_{\mathrm{ENSO}}$

The 1-year moving window used to calculate ENSO entropy ($S_{\mathrm{ENSO}}$) was adopted because the daily temporal resolution of the underlying temperature fields provides sufficient sampling within each annual window ($N = 365$) for stable entropy estimation. A substantially longer window would increase the likelihood of averaging across successive opposite-phase ENSO events (e.g., the 1997 El Niño followed by the 1998 La Niña), potentially causing signal cancellation and obscuring the temporal evolution of ENSO complexity. This choice is also consistent with previous studies that have used annual windows to characterize the temporal evolution of ENSO variability [4, 5].

## S3.3 Two-month window for correlation analysis

In Fig. 3 and Supplementary Fig. 10, a 2-month moving average was used to analyze monthly soil moisture anomalies in the positive and negative $U_1$ phase regions (SM $U_1^P$ and SM $U_1^N$), as well as their inter-regional difference ($\delta_{\mathrm{SM}}$). This was done to match the bi-monthly definition of the MEI.v2 index, enabling a more direct comparison between soil moisture variability and ENSO intensity.

Repeating the analysis using unsmoothed monthly anomalies produced highly consistent results (Supplementary Fig. 24), confirming that the inferred ENSO–soil moisture relationship is insensitive to the choice of temporal aggregation.

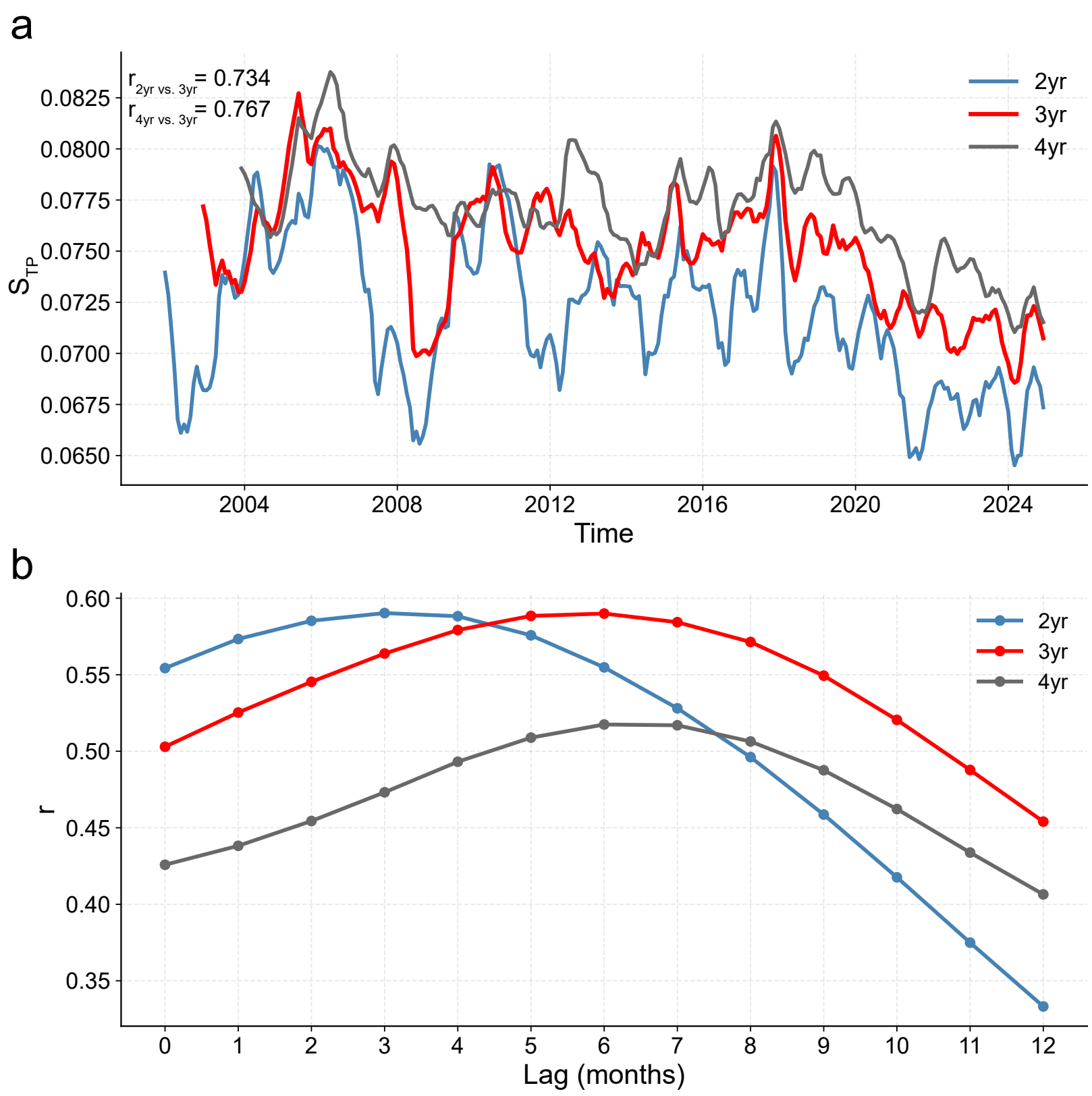


**Fig. S23 Sensitivity analysis of moving-window length for $S_{\mathrm{TP}}$ estimation.** (**a**) Comparison of $S_{\mathrm{TP}}$ derived using 2-, 3-, and 4-year moving windows, showing high consistency with the 3-year estimate. (**b**) Lag correlations between $S_{\mathrm{TP}}$ and $S_{\mathrm{ENSO}}$ across window lengths, confirming a robust lead–lag relationship.

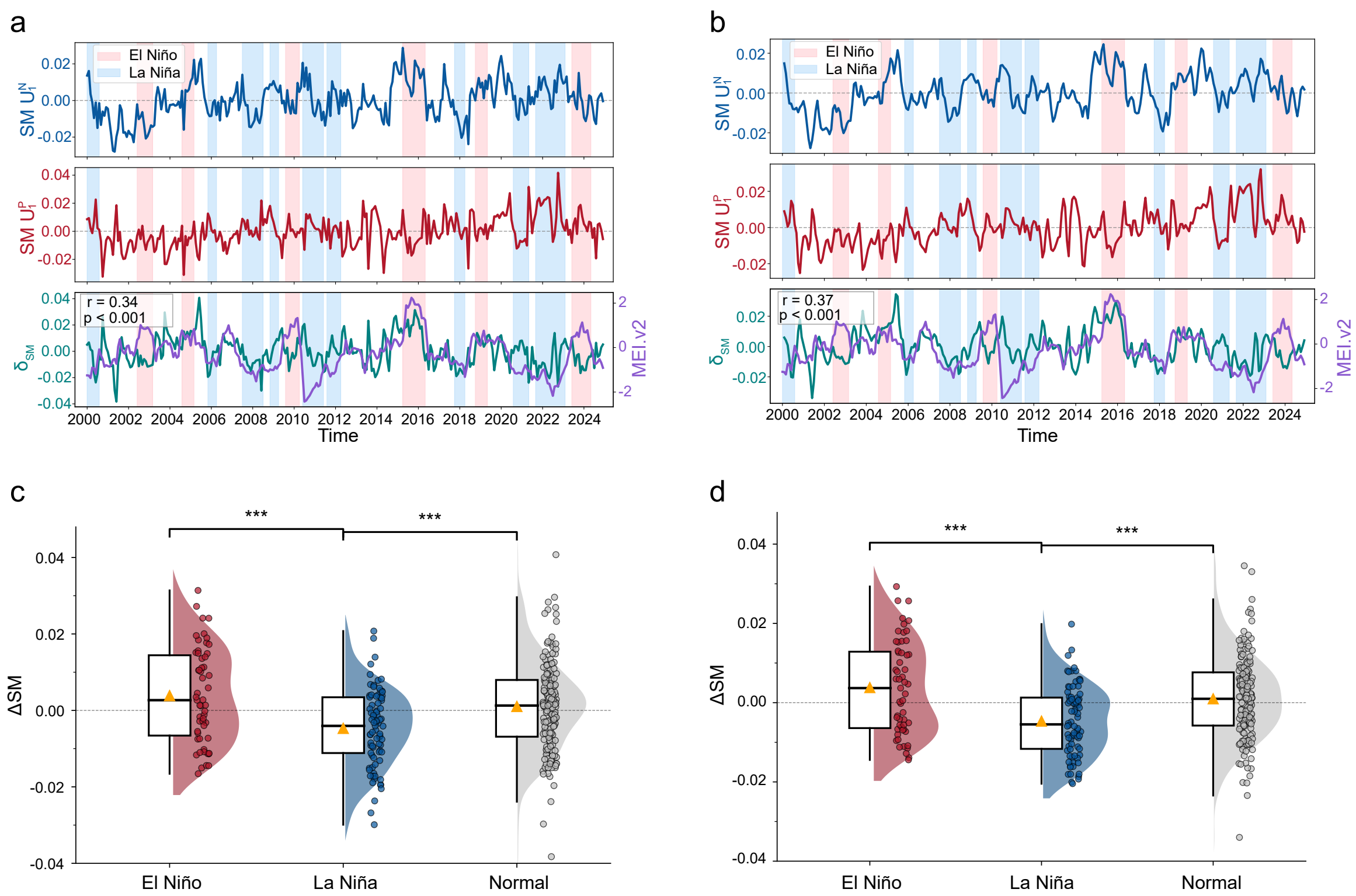


**Fig. S24 Insensitivity of the relationship between SM spatial heterogeneity and ENSO to smoothing window.** (**a**) and (**b**) show the SM anomalies in the negative and positive $U_1$ phase regions (SM $U_1^N$ and SM $U_1^P$, respectively), together with their inter-regional difference ($\delta_{\mathrm{SM}}$) and the MEI.v2 index. Panel (**b**) applies a 2-month running mean to SM-related variables, while panel (**a**) shows monthly values. The correlations between $\delta_{\mathrm{SM}}$ and MEI.v2 are 0.34 and 0.37, respectively ($p < 0.001$), indicating weak sensitivity to smoothing. (**c**) and (**d**) show violin plots of $\delta_{\mathrm{SM}}$ based on monthly values and a 2-month running mean, respectively, during El Niño (red), La Niña (blue), and neutral (gray) periods. Boxplots show the median, interquartile range (IQR), whiskers within 1.5×IQR, and mean values (yellow triangles). Significant differences (***, $p < 0.001$) were identified between El Niño and La Niña, and between La Niña and neutral, based on one-way ANOVA and two-sided Mann–Whitney $U$-test. Both cases consistently show enhanced (reduced) $\delta_{\mathrm{SM}}$ during El Niño (La Niña), with weak contrasts under neutral conditions.

# References


[1] Heginbottom, J. A., Brown, J., Ferrians, O. & Melnikov, E. S. Circum-arctic map of permafrost and ground-ice conditions, version 2 (2002). URL https://nsidc.org/data/GGD318/versions/2.

[2] Deser, C., Knutti, R., Solomon, S. & Phillips, A. S. Communication of the role of natural variability in future north american climate. *Nat. Clim. Change* **2**, 775–779 (2012).

[3] Eyring, V. *et al.* Overview of the coupled model intercomparison project phase 6 (CMIP6) experimental design and organization. *Geosci. Model Dev.* **9**, 1937–1958 (2016).

[4] Yamasaki, K., Gozolchiani, A. & Havlin, S. Climate networks around the globe are significantly affected by El Niño. *Phys. Rev. Lett.* **100**, 228501 (2008).

[5] Fan, J., Meng, J., Ashkenazy, Y., Havlin, S. & Schellnhuber, H. J. Network analysis reveals strongly localized impacts of El Niño. *Proc. Natl. Acad. Sci.* **114**, 7543–7548 (2017).
