# Supplementary material for: Warming-driven rise in soil moisture entropy signals growing instability risk in the Asian Water Tower
Source: Nat Commun. 2026 Aug 21;17:10012. doi: 10.1038/s41467-026-76955-w (PMC13590596; doi:10.1038/s41467-026-76955-w)
Supplement: Supplementary file 2 [file 41467_2026_76955_MOESM2_ESM.pdf]

nature portfolio

Peer Review File

Warming-driven rise in soil moisture entropy signals growing instability risk in the Asian Water Tower

Corresponding Author: Dr Teng Liu

This file contains all reviewer reports in order by version, followed by all author rebuttals in order by version.

Version 0:

Reviewer comments:

Reviewer #1

(Remarks to the Author)
This study presents an innovative application of eigen microstate entropy to diagnose the stability of the Asian Water Tower, revealing a concerning reversal from recent ordering to projected disorder under warming. The framework is compelling and the teleconnection analysis with ENSO is well supported. However, I have several concerns that should be addressed to strengthen the conclusions.

(1) While the authors carefully evaluated three SM products against in-situ data, the ground-based network in TP remains sparse in terms of both spatial coverage and temporal extent (2009–2019), and is largely limited to the top 5–10 cm. High-altitude regions with permafrost and complex terrain are severely under-represented. The conclusion that GLDAS-NOAH performs best does not fully resolve the issue, as all reanalysis and model-based products share structural uncertainties in representing soil moisture dynamics over cold, high-relief landscapes.

(2) The future analysis relies on CMIP6 soil moisture from the upper layer, but on the TP, soil moisture dynamics are strongly coupled with snow accumulation, glacier melt, and permafrost thaw—processes that are poorly represented in most CMIP6 models. The projected increase in entropy and the modeled regime shift in IPSL-CM6A-LR are attributed largely to precipitation-driven changes, yet the role of declining snowpack, earlier snowmelt, and increasing contributions from glacial meltwater to soil moisture is not systematically evaluated. Given that the "Asian Water Tower" is defined by its cryospheric water storage, the omission of these components in the entropy framework may lead to an incomplete assessment of future hydrological stability. So, it is better to incorporate cryospheric variables (e.g., snow water equivalent, glacier runoff) into the entropy analysis or explicitly discussing how their exclusion could alter the projected instability signal.

(3) Supplementary Table 1 lists the 12 CMIP6 models used, but the criteria for selecting these models (based on historical STPSTP performance) are described only briefly. Please add a quantitative metric (e.g., correlation threshold or skill score) to make the selection process more transparent and reproducible.

(4) The coarse resolution of GLDAS-NOAH (0.25°) may smooth out fine-scale heterogeneity, especially in steep terrain in TP. Please add more discussions.

Reviewer #2

(Remarks to the Author)

As one of the climate sensitive regions, the destabilization of Tibetan Plateau would have potentially profound effects on global climate. Soil moisture dynamics, one perspective of stability of Tibetan Plateau, remain poorly quantified especially regarding its quantification and its change with ENSO events. The authors implemented the concept of entropy of soil moisture to diagnose the disorder and internal heterogeneity of the Tibetan Plateau system. Results suggested that the declining entropy of soil moisture indicates an increasing orsdered state of the system over the period 2000-2024. The spatial-temporal patterns of the entropy of soil moisture are significantly correlated with ENSO events. The future warming trend especially under SSP585, however, would drive a transition from an ordered to a more disordered state, underscoring substantial risks for climate feedback and water security. In general, the paper is clearly written, and the results are very interesting. However, there are still two major limitations that need to be addressed before publication.

1.The introduction is not well written.
The importance of Tibetan Plateau is mentioned extensively while the selling points of this paper are not well described

especially in the first paragraph. For instance, how has the “stability” of Tibetan Plateau been studied before? What has been previously reported regarding the impacts of ENSO events on the Tibetan Plateau? Why would the entropy of soil moisture be chosen instead of other parameters, such as precipitation and temperature?
In addition, the last paragraph of Introduction shall be what will be investigated in this study instead of conclusions.

2.The discussion section does not address implications so well. For example, the authors mentioned the distinct dipole (western negative phases- wetter regions vs southwestern positive phases-drier regions) and cited previous study that negative phase in western regions would cast impacts on the Yangtze River Basin. Then what will the positive phase in southern region impact? What is the new knowledge that the results would contribute to, when we understand ENSO would impact precipitation anomalies and soil moisture? The reason why this question is raised is because the impacts of ENSO events on the Tibetan Plateau has been studied.

Minor comments:
1. Is it possible to use the same colorbar for Fig.3a and Fig.5d so that the amplification of soil moisture entropy would be more explicit?
2. Figure caption in Fig.5: “ensemble-mean…. Of soil moisture mode (U1) under SSP585”, which ensemble? Is the mean of C1-C4?
3. The lag of six months between SENSO and STP is later not used in the smoothing window analysis. Instead, three-year or two month smoothing window were used for comparison. The choices of the sizes of smoothing window seem to be very subjectively, any support to the choices?

Reviewer #3

(Remarks to the Author)
The manuscript “Warming-driven rise in soil moisture entropy signals destabilization of the Asian Water Tower” presents a novel application with the use of Eigen Microstate entropy to Tibetan Plateau (TP) soil moisture. It combines observational products, entropy-based diagnostics, ENSO teleconnections, and CMIP6 projections to show the connection of that recent wetting has reduced entropy and increased hydrological organization. Moreover that future warming is projected to increase entropy and regional contrasts, potentially signaling destabilization and even the risk of regime shifts in some models. The study is methodologically rigorous and provides essential insides into telecoupling of Earth system elements across long distances, and it has the potential to make a strong contribution.

Overall I find the main storyline clear and compelling, but somewhat overloaded with figures and partly redundant diagnostics spread across the main text and Supplement).
Next to this, the Results section contains already substantial interpretive and discussion-like sections that should be moved to Discussion. Interestingly, (and perhaps as a result of this), the authors chose in the Discussion section not to refer to any figure or result (e.g. missing references to own findings or numbers from Results) to allow to connect their claims with own specific quantitative findings but rather refer to other studies
So my recommendations are i) streamline the storyline by reducing the analytics to the relevant figures only, ii) move interpretations and discussions from Results to Diskussion, iii) connect and refer in the Discussion with findings from this study

Therefore I recommend major revisions (as mainly moving existing text) for this manuscript

Figures:
The current main essential main-text figures to keep (Fig. 1 – Fig 4, and Fig 5 (partly)
Within Fig. 5, panels (b), (d), (e), and (f) are directly tied to the main conclusions on future entropy changes and potential regime shifts. However, the LFCA conceptual model (Fig. 5a) and the detailed eigen-mode contribution panel (Fig. 5c) are nice but not essential for the main text. I recommend moving these to the Supplement and referring to them there when introducing entropy as an early-warning metric.

In the Supplement, several figures are primarily diagnostic or partially redundant with main-text plots. For example, S5–S7 (various forms of STP–ENSO correlation), S9 (PSD of V1), S12 (detailed monthly climatologies for the dipole regions), S19 (V1 vs temperature in IPSL-CM6A-LR), and S21 (ONI-based ENSO event identification) could potentially be merged, simplified, or even removed without loss of the main message.

Results

The results section is generally well-structured going through observations, entropy behavior, ENSO modulation, and projections.
However, here frequently the goes beyond reporting results or observations of data behavior into mechanistic speculation, broader system implications, and even discussion of socio-hydrological impacts.

While these interpretations are reasonable and justified, they would be much better placed in the Discussion. For examples

L 190: attributing the SM–entropy relationship to “greater hydrological buffering capacity of wetter soils” and detailed ecological feedbacks.

L 172 ff: proposing multi-step feedback chains (e.g., vegetation composition shifts, land–atmosphere feedbacks, cloud changes) in 2.2–2.4.

L 356 ff: descriptions of cascading risks to downstream water resources and food security already hinted at in the Results.

Also the extended passages on buffering capacity, vegetation–soil feedbacks, and tipping cascades can be moved into the Discussion, where they can be integrated with the broader literature and discussed more cautiously.

Discussion

As already mentioned above the Discussion broadly reflects the results but often speaks at a higher conseptual level without consistently referring back to specific figures or quantitative numbers.
For instance, statements about “new evidence for such teleconnections” and ENSO modulation of TP SM entropy should explicitly referenced to own findings such as ‘the six-month lagged correlation between STP and SENSO’ and the corresponding figure(s), and the spatial dipole of EM1 and its link to δSM and STP.

Likewise, when discussing future destabilization, rise in entropy, and mid-century regime shifts, the Discussion should e.g explicitly cite the ‘projected upward trends in STP’ (figure reference and approximate trend magnitudes). Also the identified breakpoint timing in entropy and V1, and the model-specific trajectory of IPSL-CM6A-LR, including which spatial regions dry vs remain wetter.
So, the Discussion would greatly improve by adding targeted back-references (figure numbers and a few key quantitative values) showing a much clearer logical connection between evidence to interpretation and help a reader better to track where each statement is supported.

Minor:

1) The authors note that originally entropy is only a measure of structural organization rather than a direct stability metric. However, much of the language (“destabilization”, “early-warning signal”, “critical transition”) reads as if entropy is being used as a near-direct proxy for system resilience.

2) The connection to Earth system tipping elements and tipping cascades is good. However, the most compelling evidence for an actual regime-like shift is found in one CMIP6 model (IPSL-CM6A-LR), while other models show smoother changes. This should be emphasized more clearly in the Discussion and Conclusions.

3) Moreover CMIP6 are just models of what we currently know how the Earth system is functioning but we are not sure if the same interactions will still be valid under future Earth system states. Thus, the authors should rater frame their results more carefully rather as heightened risk of nonlinear transitions and plausible early-warning signals rather than as evidence that such tipping is likely or inevitable. Explicitly acknowledging current limitations in CMIP6’s representation of TP hydrology, snow and permafrost processes, and land–atmosphere coupling, and clarifying that some abrupt shifts might be model-specific artifacts

4) Avoid oscillating within a subsection between present and future, or between ENSO and non-ENSO drivers; this will make each block easier to follow.

5) Some descriptive passages closely restate what is already visually obvious from multiple panels (e.g. repeated explanations of El Niño increasing spatial contrasts and La Niña reducing them). Trimming these repeated descriptions will free space for more concise, evidence-based linking of results and discussion.

6) Add information on the chosen length and step of moving windows used for entropy and correlation calculations and number of modes retained or focused on in figures (e.g. rationale for limiting to the first four eigen microstates).

7) Given the fact that there are already too many figures the authors may consider to potentially add a conceptual figure on diagnostic steps. Add one small conceptual workflow panel, as a panel in Fig. 1 (e.g. Fig. 1e), or in the Methods as Fig. M1 (if the journal allows), or in SI if space is tight.

A compact schematic of the workflow from datasets → EMT/entropy → ENSO link → dipole mechanism → CMIP6 projections). This could help non-specialist readers follow a fairly technical pipeline and Clarify where each method/figure fits. Keep it very simple: boxes and arrows, not detailed equations.

Version 1:

Reviewer comments:

Reviewer #1

(Remarks to the Author)

I greatly appreciate the authors' detailed response, which has effectively addressed my previous questions. In consideration of the uncertainties also mentioned by the other reviewers, I have carefully examined the site comparisons in the attached Figure FS2 and found that the error, or discrepancy, at the Naqu site is considerably large. I would kindly ask the authors to explain this phenomenon, specifically regarding whether it results from inherent errors at the monitoring station itself or from uncertainties induced by the particular conditions at this site.

Reviewer #2

(Remarks to the Author)
Thank you for your thorough and thoughtful revisions. All my concerns have been fully addressed. Great work!

Reviewer #3

(Remarks to the Author)
The authors have addressed the major issues by streamlining the manuscript's storyline and structure, including merging figures where necessary, moving the interpretations from the 'Results' section to the 'Discussion' section, and changing the wording to a more appropriate style with more careful phrasing. They have also provided more rigorous references for the discussion points in the figures and tables.

Open Access This Peer Review File is licensed under a Creative Commons Attribution 4.0 International License, which permits use, sharing, adaptation, distribution and reproduction in any medium or format, as long as you give appropriate credit to the original author(s) and the source, provide a link to the Creative Commons license, and indicate if changes were made.
In cases where reviewers are anonymous, credit should be given to 'Anonymous Referee' and the source.
The images or other third party material in this Peer Review File are included in the article's Creative Commons license, unless indicated otherwise in a credit line to the material. If material is not included in the article's Creative Commons license and your intended use is not permitted by statutory regulation or exceeds the permitted use, you will need to obtain permission directly from the copyright holder.
To view a copy of this license, visit https://creativecommons.org/licenses/by/4.0/

Point-by-point response to Reviewers' comments

In the following, the referee comments will be shown in blue, our responses in black, original manuscript content in *black italics*, and the quoted changes we made to the manuscript main text in *green italics*.

Reviewer #1:

This study presents an innovative application of eigen microstate entropy to diagnose the stability of the Asian Water Tower, revealing a concerning reversal from recent ordering to projected disorder under warming. The framework is compelling and the teleconnection analysis with ENSO is well supported. However, I have several concerns that should be addressed to strengthen the conclusions.

Response: We sincerely thank the reviewer for the positive evaluation of our work. We are highly encouraged that the reviewer found our application of eigen microstate entropy innovative, our framework compelling, and the teleconnection analysis with ENSO well supported.

We have taken all of the reviewer's concerns into serious consideration and thoroughly revised the manuscript. The major improvements in the revised version include:

(1) Uncertainty discussion: We comprehensively discussed the limitations of sparse SM in-situ observations along with the structural uncertainties inherent in data assimilation–based and reanalysis products across cold, high-relief landscapes. Furthermore, we conducted additional sensitivity experiments–specifically by excluding permafrost regions and introducing multiple remote sensing products–demonstrating that our primary findings are robust against these data uncertainties.

(2) Cryospheric impact: We analyzed the evolutionary dynamics of key cryospheric variables, including snowmelt and snow water equivalent, and evaluated how the exclusion of these cryospheric dynamics in the models could lead to an underestimation of the projected ecosystem instability signals.

(3) CMIP6 model selection metrics: We provided explicit quantitative metrics to make our CMIP6 model selection criteria fully transparent and reproducible.

(4) Resolution sensitivity discussion: We expanded our discussion on how spatial resolution affects the entropy results, justifying the appropriateness of our macro-scale, system-level analysis.

We believe that these updates have substantially improved the quality of the manuscript. Our detailed, point-by-point responses to each specific concern follow below.

(1) While the authors carefully evaluated three SM products against in-situ data, the ground-based network in TP remains sparse in terms of both spatial coverage and temporal extent (2009–2019), and is largely limited to the top 5–10 cm. High-altitude regions with permafrost and complex terrain are severely under-represented. The conclusion that GLDAS-NOAH performs best does not fully resolve the issue, as all reanalysis and model-based products share structural uncertainties in representing soil moisture dynamics over cold, high-relief landscapes.

Response: We thank the reviewer for this insightful comment. We agree that soil moisture datasets over the TP are subject to important limitations, including the sparse spatial and temporal coverage of in-situ observations and the inherent challenges faced by reanalysis and model-based products in representing soil moisture dynamics over cold, high-elevation, and topographically complex environments. Therefore, we have substantially revised the manuscript to provide a more comprehensive assessment of dataset-related limitations and uncertainties. Specifically, we (1) expanded the Discussion section to address the limitations of the in-situ observation network and the structural uncertainties inherent in existing soil moisture products, (2) conducted a sensitivity analyses by excluding permafrost regions to evaluate the permafrost-related uncertainties on our conclusions, and (3) performed an independent robustness assessment using multiple satellite-based soil moisture datasets. These additional analyses consistently indicate that the principal entropy variability and ENSO teleconnections identified in this study remain robust to these source of data-related uncertainty. Detailed responses and corresponding revisions are provided below.

(1) Limitations of in-situ observations in the TP

We fully acknowledge these limitations of the in-situ SM network over the TP sparse spatial coverage, a limited record (2009–2019), and shallow measurement depth (5–10 cm), which are most pronounced in high-elevation and permafrost regions. The manuscript has been revised accordingly to state these constraints explicitly. The corresponding text added to the main text is quoted at the end of this response.

(2) Sensitivity analysis regarding permafrost-region uncertainty

We agree with the reviewer's concern that the sparse observational coverage in permafrost regions may introduce additional uncertainty into SM estimation over these areas. To evaluate the influence of permafrost, we excluded permafrost regions and recalculated SM entropy. Permafrost regions were identified using the Circum-Arctic Map of Permafrost and Ground-Ice Conditions (Version 2) provided by the National Snow and Ice Data Center [1]. Areas classified as continuous and discontinuous permafrost (permafrost extent $\geq$ 50%) were defined as permafrost regions, whereas sporadic and isolated permafrost areas (permafrost extent <50%) were treated as non-permafrost regions (Supplementary Fig. 20a). The resulting classification shows that permafrost regions are primarily located in the northwestern TP and generally exhibit lower mean SM than non-permafrost regions (Supplementary Fig. 20b).

As shown in Supplementary Fig. 20c, the SM entropy derived from the non-permafrost region closely follows that of the entire TP, with a correlation coefficient of 0.95. This high consistency suggests that the dominant entropy variability identified over the TP is preserved even when permafrost regions are excluded, indicating that our main findings are insensitive to whether permafrost regions are included. Crucially, significant lagged relationships between TP soil moisture and ENSO disorder (S_{TP} v.s. S_{ENSO}) persist in both permafrost and non-permafrost regions (correlation coefficients exceeding 0.53; Supplementary Fig. 20d). Beyond merely leaving the signal intact, the permafrost analysis yields a physically meaningful refinement: the permafrost region exhibits a distinctly longer time lag than the non-permafrost region (blue and red lines in Supplementary Fig. 20d). This is what the underlying physics predicts: freeze–thaw processes prolong soil-moisture memory and thereby delay the hydrological response to climate forcing. The permafrost experiment therefore does more

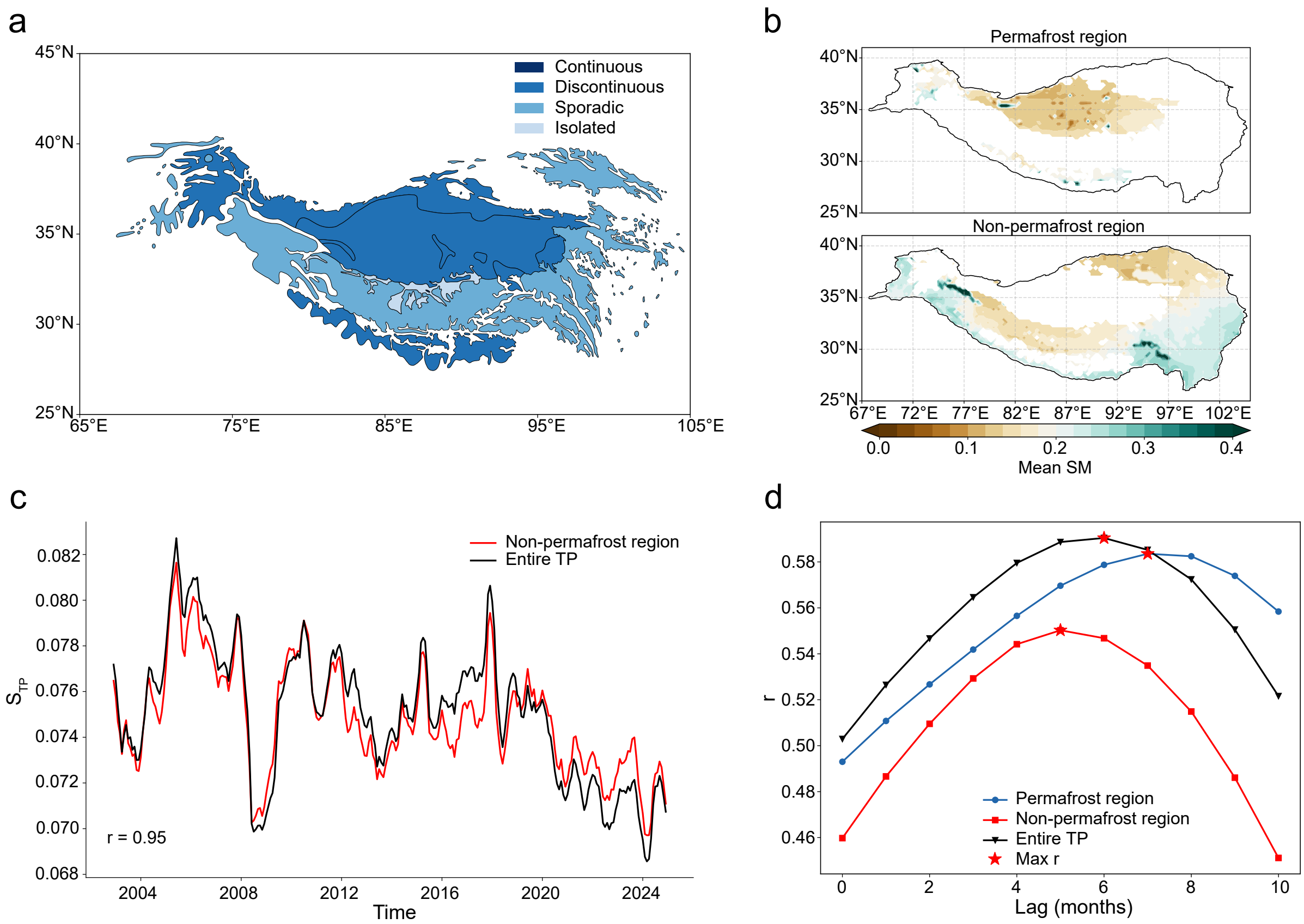

Supplementary Figure S20: Sensitivity analysis of permafrost-related uncertainty in SM entropy over the TP. Permafrost regions were identified using the Circum-Arctic Map of Permafrost and Ground-Ice Conditions (Version 2) provided by the National Snow and Ice Data Center [1]. (**a**) Spatial distribution of permafrost and non-permafrost regions over the TP. Continuous and discontinuous permafrost (permafrost extent $\geq$ 50%) are classified as permafrost regions, whereas sporadic and isolated permafrost (permafrost extent <50%) are classified as non-permafrost regions. (**b**) Mean SM distribution in permafrost and non-permafrost regions. (**c**) Temporal evolution of SM entropy over the all TP and non-permafrost regions. (**d**) Lagged correlations between ENSO entropy and SM entropy for the entire TP and non-permafrost regions.

than rule out a data artefact; it independently reproduces a known cryospheric mechanism, lending additional physical credibility to the diagnosed ENSO–TP coupling.

Together, these results suggest that uncertainties associated with the under-representation of permafrost regions are unlikely to alter the principal entropy variability and ENSO teleconnection identified in this study. However, as critically addressed in our expanded Discussion, these sensitivity check results do not diminish the importance of acknowledging the systemic uncertainties inherent in current SM products. Such uncertainties remain particularly relevant in the TP's cold, high-elevation, and topographically complex environments, where limited observations and complex land-surface processes continue to pose challenges for SM estimation across most reanalysis and model-based datasets. Future efforts would highly benefit from the integration of higher-resolution observations and improved representations of cold-region hydrological processes to further reduce these uncertainties and strengthen our understanding of SM system dynamics over the TP.

(3) Sensitivity analysis using satellite-based SM datasets

We agree with the reviewer's concern that the relatively better performance of GLDAS-NOAH does not resolve all of the uncertainties associated with SM estimation over the TP. As the reviewer noted, reanalysis and model-based products inevitably contain structural uncertainties. To address this, we have expanded the discussion of these dataset-related uncertainties in the revised manuscript.

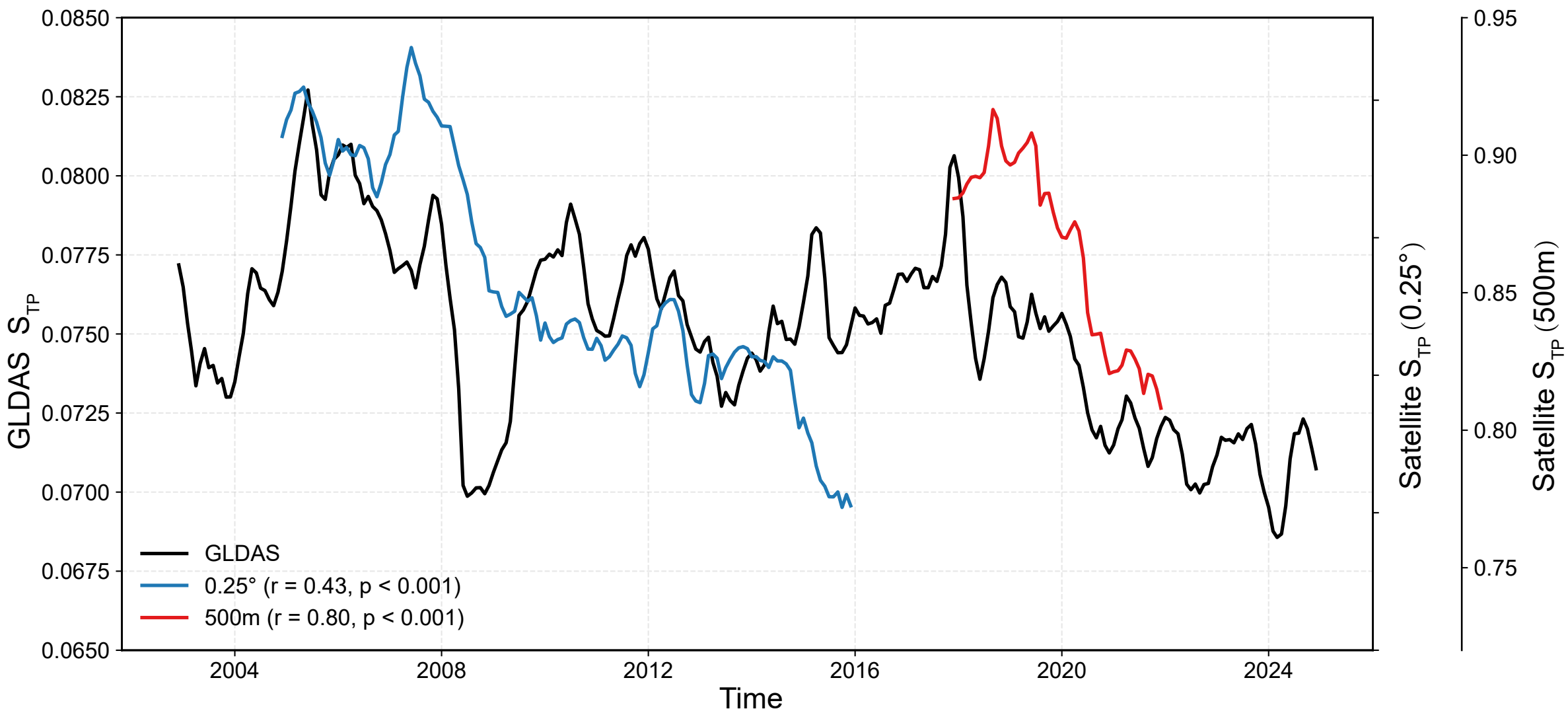

Supplementary Figure S5: Consistent temporal evolution of S_{TP} across multiple SM datasets. Blue and red lines represent TP satellite-based SM products at 0.25° (2002–2015) and 500m (2015–2021), respectively, both showing significant positive correlations with GLDAS-NOAH. For the 500 m dataset, the evaluation was restricted to 2015–2021 to align with its available in-situ observations.

In addition, we conducted an independent robustness assessment using two alternative satellite-based SM datasets optimized for the TP. These include a 0.25° spatio-temporal continuous dataset (2002–2015) based on the ECV satellite soil moisture product [2], and a 500m high-resolution dataset (2015–2021) from the National Tibetan Plateau Data Center based on SMAP microwave product [3]. As shown in Supplementary Fig. 5, the SM entropy derived from these satellite products exhibits consistency with GLDAS-NOAH results. This agreement indicates that the identified variability in TP SM system disorder is robust across these independent datasets rather than being specific to GLDAS-NOAH.

While these satellite products avoid the structural uncertainties shared by reanalysis and model-based datasets, they are themselves limited in temporal coverage: the currently available products span only shorter, fragmented intervals (2002–2015 and 2015–2021) that cannot be merged without introducing severe systematic inconsistencies. Such short and discontinuous records are inherently unsuitable for the long-term, climate-scale analysis of SM system stability and ENSO teleconnections that is central to this study. We therefore used these satellite datasets strictly for independent, segment-wise robustness assessments, while retaining GLDAS-NOAH as the primary, temporally continuous data source.

The corresponding additions to the *Methods* and *Results* are as follows:

"In addition, two satellite-based surface SM datasets were incorporated to validate the consistency of the identified SM entropy evolution in the TP: a 0.25° product (2002–2015) based on ECV microwave products [84], and a 500 m *product (2015–2021) derived from SMAP microwave data [85]. "* (Methods,

Line 501)

"*The entropy of the TP's SM (S_{TP}) declined significantly during 2000–2024 (Fig. 2a), with a slope of* $-0.000021\ \mathrm{yr}^{-1}$ *(*$p < 0.001$*), indicating an increasingly ordered system under prevailing wetting conditions. Consistent variations in* S_{TP} *are also found across multiple satellite-based SM datasets, further supporting the robustness of the results (Supplementary Fig. 5).*" (Results, Line 215)

Taken together, the in-situ, permafrost, and satellite considerations above are consolidated into a new dedicated uncertainty paragraph added to the *Discussion and Conclusion*:

"A further source of uncertainty lies in the observational data themselves. The in-situ SM network over the TP remains sparse, with limited spatial coverage, a relatively short record (2009–2019), and measurements largely confined to the upper $5-10$ cm *of the soil profile. These gaps are most acute in permafrost regions, which are important to the TP water balance yet remain severely undersampled. Because permafrost governs a substantial fraction of the Plateau's frozen-water storage and freeze–thaw dynamics, we explicitly tested its influence by recomputing entropy with permafrost areas excluded. The resulting entropy evolution and ENSO teleconnection remained broadly consistent with the full-domain analysis (Supplementary Fig. 20), indicating that our principal conclusions are robust to uncertainties in permafrost-region SM estimates. The somewhat longer lagged response found in permafrost regions is itself physically consistent with the role of freeze–thaw processes in prolonging SM memory, lending additional physical coherence to the diagnosed ENSO–TP coupling. Beyond the in-situ network, residual uncertainties in the underlying assimilated SM product cannot be fully excluded over the TP's cold, high-elevation, and topographically complex terrain, where SM estimation remains challenging for most reanalysis and model-based datasets [33, 34]... Consistent with this, the SM entropy evolution remains stable across independent satellite products at substantially finer resolutions (*$0.25°$ *and* 500 m*, Supplementary Fig. 5)..."* (Discussion and Conclusion, Line 450)

Overall, although uncertainties associated with existing SM products remain, the consistency across independent satellite observations, together with the additional permafrost-region sensitivity analysis, reinforces the robustness of the principal entropy variability and ENSO teleconnections identified in this study. The revised manuscript has been updated accordingly to explicitly discuss these limitations and include the additional sensitivity checks described above.

(2) The future analysis relies on CMIP6 soil moisture from the upper layer, but on the TP, soil moisture dynamics are strongly coupled with snow accumulation, glacier melt, and permafrost thaw–processes that are poorly represented in most CMIP6 models. The projected increase in entropy and the modeled regime shift in IPSL-CM6A-LR are attributed largely to precipitation-driven changes, yet the role of declining snowpack, earlier snowmelt, and increasing contributions from glacial meltwater to soil moisture is not systematically evaluated. Given that the "Asian Water Tower" is defined by its cryospheric water storage, the omission of these components in the entropy framework may lead to an incomplete assessment of future hydrological stability. So, it is better to incorporate cryospheric variables (e.g., snow water equivalent, glacier runoff) into the entropy analysis or explicitly discussing how their exclusion could alter the projected instability signal.

Response: We sincerely thank the reviewer for this exceptionally constructive comment. We agree

that on the TP (the "Asian Water Tower"), SM dynamics are tightly coupled with cryospheric processes, as the reviewer mentioned, including snow accumulation, glacier melt, and permafrost thaw, which are poorly represented in most CMIP6 models. Indeed, our original statistical attribution pointed heavily toward precipitation-driven changes, leaving the role of these cryospheric components and their potential contributions to SM system disorder unsystematically evaluated. To thoroughly address this crucial point, we have incorporated cryospheric variables into our supplementary analyses, which significantly enhances the discussion in the revised manuscript. We break down our response into the following aspects:

(1) Limitations of CMIP6 soil moisture simulations over the TP

We explicitly acknowledge that CMIP6 models inherently carry systematic deficiencies in characterizing complex cryospheric dynamics over the TP. Most simulations rely on simplified land surface schemes that poorly represent cryospheric processes such as sub-grid glacier runoff, permafrost active-layer degradation, and precise ice-land-atmosphere energy partitioning. Because these cryospheric pathways are underrepresented, the modeled upper-layer soil moisture exhibits excessive sensitivity to future atmospheric inputs such. This model-driven limitation explains why our original statistical attribution pointed so heavily toward precipitation-driven changes, thereby underestimating the direct hydrological modulations stemming from a thawing cryosphere. To address these CMIP6 model uncertainties, we have added a dedicated discussion to the revised manuscript as follows:

"Second, because the Asian Water Tower is defined by its cryospheric water storage, the fidelity of the future projections depends on how well these processes are represented. CMIP6 models, while grounded in our current understanding of Earth system processes, may not fully capture system behavior under future climate states, particularly given known limitations in representing TP cryospheric dynamics and land–atmosphere coupling [71, 72]. Accordingly, while Pp emerges as the primary driver of projected entropy changes, the contributions of snow accumulation, glacier melt, permafrost thaw, and surface runoff to entropy dynamics remain insufficiently assessed and constitute an important source of uncertainty. Finally, the clearest regime-shift signal is largely confined to a particular model (IPSL-CM6A-LR), whereas other models show more gradual responses, leaving open whether such signals represent genuine thresholds or model-specific behavior. Resolving these questions will require improved representation of cryospheric, hydrological, and land–atmosphere processes together with coordinated multi-model assessments." (Discussion and Conclusion, Line 437)

(2) Potential impacts of future cryospheric and hydro-climatic dynamics on TP soil moisture system disorder

To systematically evaluate how underrepresented cryospheric processes modulate SM system stability, we analyzed the long-term projected trends of Snow Water Equivalent (SWE) and Snowmelt Flux (SWM) anomalies within the CMIP6 framework. As illustrated in Supplementary Figs. 19b,c (shown below), both SWE and SWM exhibit a downward trend that intensifies with progressive warming during 2025–2100. This clear depletion of solid-water storage reflects a shrinking seasonal snowpack over the TP. Mechanistically, this sustained snowpack degradation cuts off a vital buffer source for early-season moisture replenishment, amplifying topsoil drying. Furthermore, permafrost degradation and subsequent active-layer thickening enhance deep soil infiltration, structurally depleting upper-layer soil moisture.

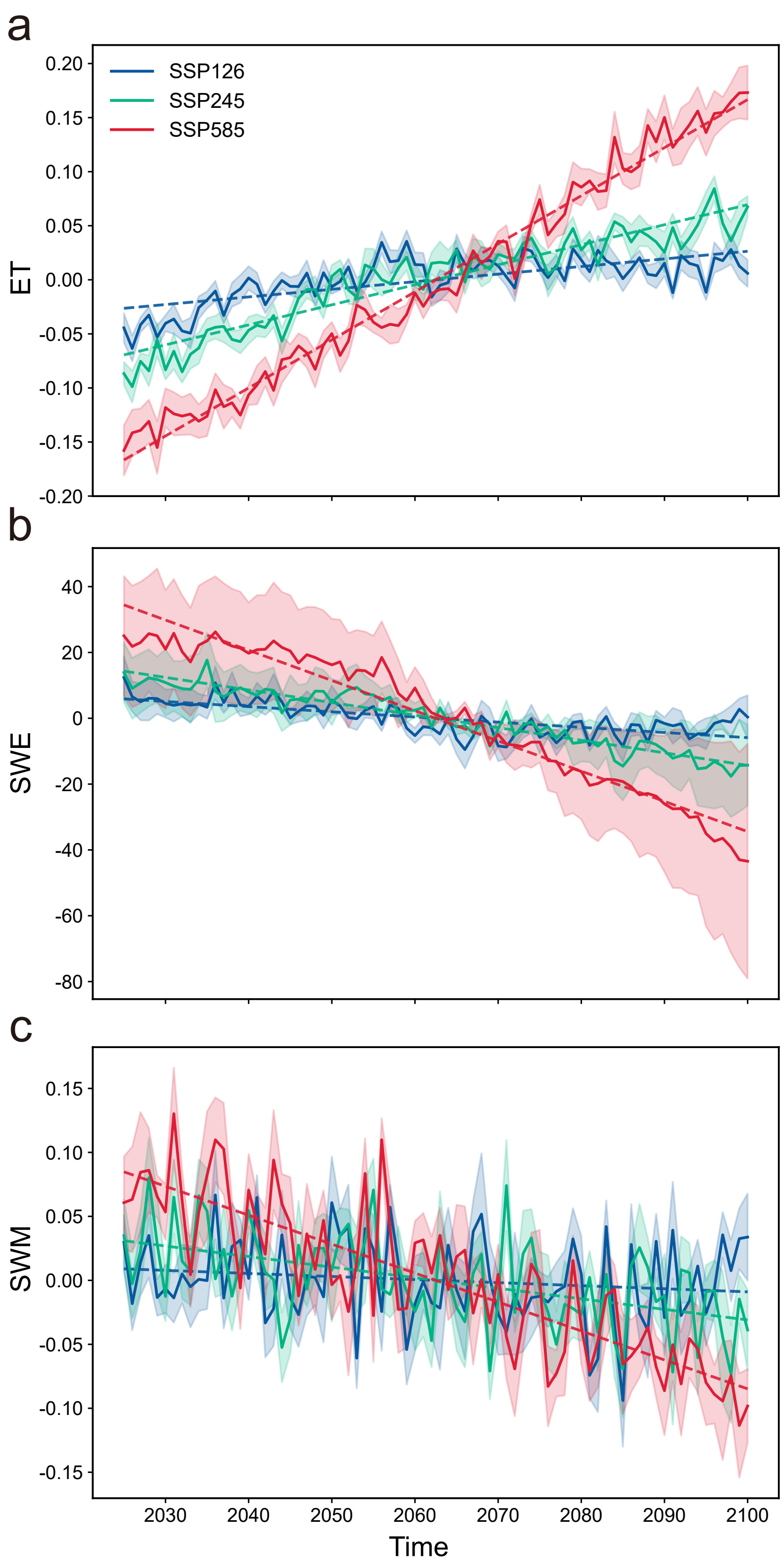

Supplementary Figure S19: Annual mean anomalies of (a) total evaporation flux (ET), (b) surface snow water equivalent (SWE), and (c) surface snowmelt flux (SWM) based on the CMIP6 multi-model ensemble mean during 20252100 under SSP126 (blue), SSP245 (green), and SSP585 (red) scenarios. Dashed lines denote linear trends. ET increases significantly while SWE decreases significantly across all scenarios ($p < 0.01$). SWM decreases significantly under SSP245 and SSP585.

Beyond these cryospheric pathways, concurrent hydro-climatic feedbacks further compound SM drying. Driven by rising temperatures, future evapotranspiration over the TP is projected to intensify significantly (Supplementary Fig. 19a), causing a direct net loss of soil water. Concurrently, increasingly concentrated future precipitation patterns structurally reduce the rainfall-to-soil-moisture conversion efficiency, shifting the water balance toward rapid surface runoff and highly evaporative open water bodies rather than soil water infiltration [4, 5].

Crucially, as established in our framework, decreasing SM implies a reduction in hydrological buffering capacity, leading to stronger responses to external forcing (e.g., enhanced atmospheric evaporative demand and cryospheric decay). These amplified responses generate larger fluctuations and a more dispersed distribution of eigen microstates, thereby accelerating the upward trend in entropy (S_{TP}) and rendering the SM system more disordered. Therefore, the underrepresentation of these compounding drying feedbacks in current models implies that our reported upward trend in SM system disorder and the associated regime shift risks are likely conservative estimates.

We further note that the cryosphere does not act on SM in a single direction, and we now address this explicitly. As the reviewer points out, enhanced glacial meltwater can transiently *increase* soil moisture, an effect that would, in isolation, reduce disorder and thus oppose our projected entropy rise. However, this wetting term neither persists nor dominates over the projection horizon, for two reasons. First, glacial meltwater is a finite, transient buffer governed by "peak water": sustained mass loss drives meltwater supply to rise and then decline once the glacier area can no longer sustain rising melt volumes. For most High Mountain Asia basins, glacier runoff is projected to peak around the middle of the century and to decline thereafter [6], albeit with substantial intra-regional variability (e.g., a later peak around 2070 in parts of the Indus headwaters, and an already-passed peak in interior-Himalayan headwaters of the Brahmaputra). Peak water is moreover reached later under higher-emission scenarios, so that under SSP585 the meltwater contribution is, if anything, sustained somewhat later before declining. In either case its contribution reverses to a long-term decline in the second half of the century, precisely the window in which the entropy increase and the ~ 2052 breakpoint emerge (Fig. 5c), so that an explicitly resolved meltwater term would reinforce rather than offset the projected drying. Second, glacial meltwater is spatially confined to glacier-fed margins and is predominantly routed to streamflow rather than broadly recharging upper layer soil moisture across the Plateau; where it does act, it sharpens the contrast between glacier-fed and precipitation-fed regions and thus adds to the cross-regional spatial heterogeneity that drives. In both its timing and its spatial expression, an explicitly represented meltwater contribution would therefore tend to raise, not lower, the projected entropy. The only countervailing effect, a S_{TP} transient early-century wetting, is limited in spatial extent and does not coincide with the projected destabilization window. This consideration strengthens, rather than weakens, our conclusion that the CMIP6-based entropy increase is most likely a conservative estimate.

We have added the above comprehensive analysis to the *Discussion and Conclusion* section of the revised manuscript as follows:

"Future projections reveal a rising trend in S_{TP} accompanied by a decline in SM, in contrast to recent decades (Fig. 5a, Supplementary Fig. 14). Declining SM implies a reduction in hydrological buffering capacity, so that the system responds more strongly to external forcing such as drought and enhanced

atmospheric evaporative demand [68]. As these responses amplify, system variability is no longer concentrated in a few coherent modes but becomes increasingly distributed across many competing eigen microstates, raising the entropy S_{TP}. Because a more even distribution over eigen microstates reflects the loss of a dominant, coherent spatiotemporal pattern, this rise in disorder signals a decline in the predictability of the TP water cycle, suggesting that water-stressed conditions weaken the coherent organization that underpins the Asian Water Tower. Understanding this projected drying therefore requires examining the processes that govern SM recharge and retention over the TP. Continued warming is expected to intensify evapotranspiration losses (Supplementary Fig. 19a), while future precipitation over the TP is projected to become more intense and temporally concentrated, causing a larger fraction of rainfall to remain in surface water stores and thereby limiting effective SM recharge [69, 70]. In addition, cryospheric processes, which are not fully represented in most CMIP6 models [71, 72], may further affect SM by reducing moisture inputs: declining snow water equivalent (SWE) and snowmelt (SWM) can reduce meltwater that sustains warm-season SM (Supplementary Figs. 19b,c) [31, 73, 74], and permafrost degradation deepens the active layer and enhances downward percolation, reducing surface-soil moisture [72, 75]. Although glacial meltwater can transiently raise SM, as a finite store subject to "peak water", projected to occur around mid-century in most High Mountain Asia basins and later under higher-emission scenarios [76], its contribution is projected to decline through the second half of the century. Moreover, most meltwater is routed to runoff rather than recharging upper-layer SM. Because lower SM reduces buffering capacity and disperses variability across competing modes, these underrepresented processes imply that the SM-driven entropy increase (Fig. 5a), projected by CMIP6, may be conservative. Continued cryosphere–hydrology feedbacks are likely to further amplify the disorder of the TP hydroclimatic system and increase the risk of system instability." (Discussion and Conclusion, Line 374)

(3) Supplementary Table 1 lists the 12 CMIP6 models used, but the criteria for selecting these models (based on historical S_{TP} performance) are described only briefly. Please add a quantitative metric (e.g., correlation threshold or skill score) to make the selection process more transparent and reproducible.

Response: We thank the reviewer for this constructive suggestion. We have now replaced the qualitative description with a fully quantitative, two-tier hybrid selection criterion that is described in detail below and in the revised *Methods* section and new added *Supplementary Note S1*.

CMIP6 historical simulations are uninitialized free-running experiments [7, 8]. Although they are driven by the same external forcings as the real world (greenhouse gases, volcanic aerosols, etc.), their internal climate variability (e.g., the timing and amplitude of individual ENSO events) evolves independently of the observed chronology. Consequently, the Pearson temporal correlation (R_T) between the time series of the CMIP6 model's S_{TP} and the GLDAS-NOAH S_{TP} is subject to phase randomisation: a model that faithfully reproduces the correct variability timescales but whose ENSO phasing differs from observations by a few years will appear to have low or even negative R_T for reasons that have nothing to do with model fidelity.

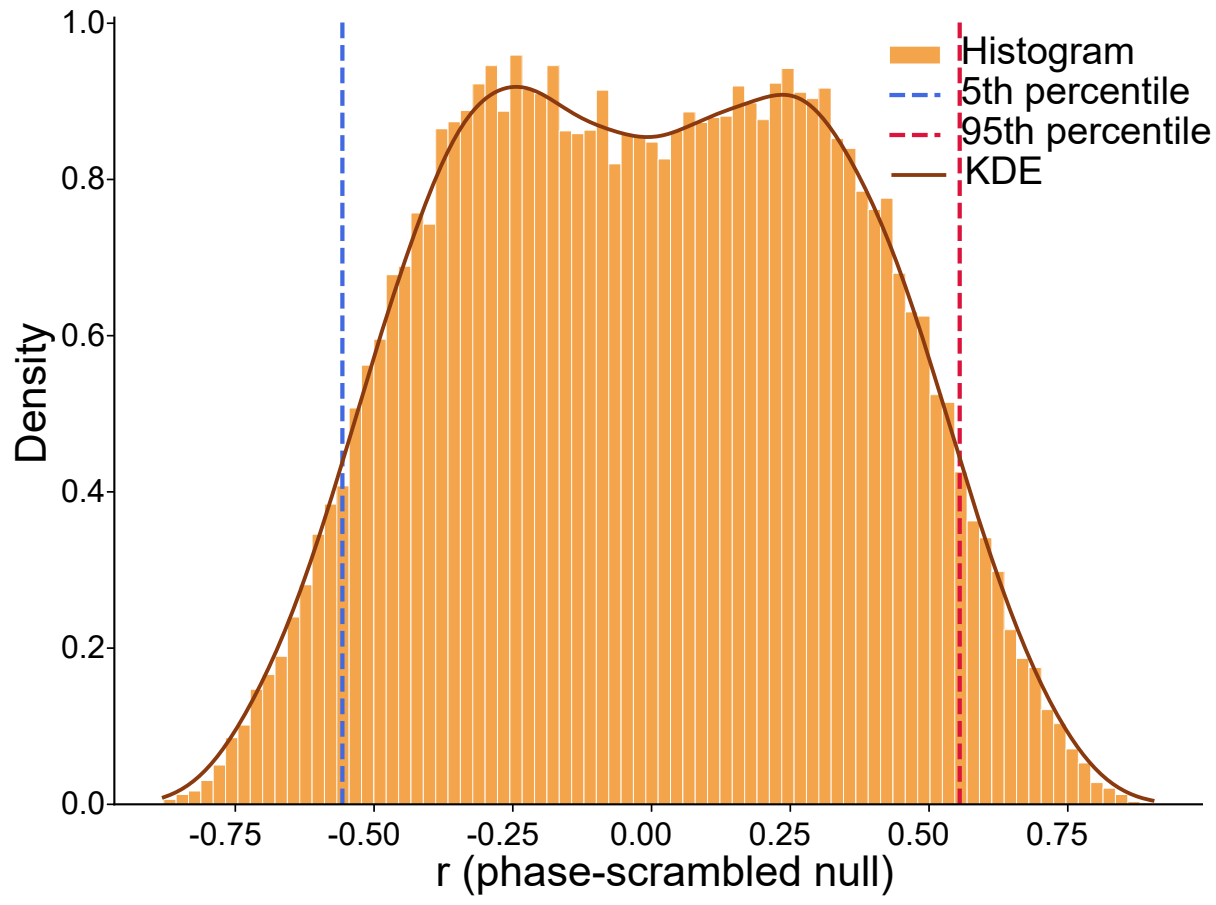

Supplementary Figure S21: Null distribution of R_T derived from phase-scrambled GLDAS surrogates.

To quantify this effect, we computed a phase-scrambling null distribution ($n = 50,000$ surrogates, random uniform phases applied to the Fourier coefficients of the GLDAS S_{TP} series, preserving its power spectral density). As shown in Supplementary Fig. 21, for $N = 145$ monthly samples, the phase-scrambling null distribution gives a standard deviation of $\sigma(r_T) = 0.35$, with corresponding 5th and 95th percentile values of -0.56 and $+0.56$, respectively. This confirms that a model with correct spectral structure but random ENSO phase has essentially a coin-flip chance of exceeding any moderate R_T threshold. Standard significance thresholds ($R_T > 0$, $p < 0.1$ from a t-distribution) are necessary but not sufficient: they would correctly retain well-performing models but would also incorrectly reject models whose phase mismatch is consistent with natural internal variability.

We therefore adopt a two-tier hybrid criterion that uses R_T as the primary filter while introducing a spectral correlation metric (R_F) as a physically motivated secondary screen for phase-uncertain models. The specific details are detailed below:

(1) Metric definitions:

Temporal correlation (R_T): Pearson correlation between the model's historical S_{TP} and the GLDAS-NOAH S_{TP} over the 2000–2014 overlap period.

Spectral correlation (R_F): Pearson correlation between the spectral amplitudes of the two standardised S_{TP} series. R_F measures whether the model reproduces the dominant variability timescales of S_{TP} irrespective of temporal phase. Because R_F is computed on the spectral information rather than the time series itself, it is insensitive to ENSO phase offsets that plague R_T.

(2) Two-tier selection criterion:

A model is selected if it satisfies either of the following tiers:

Tier 1 (temporal skill): $R_T > 0$ and $p < 0.1$. Retains models with a statistically significant positive temporal correlation.

Tier 2 (phase-uncertain models): $|R_T| < 0.01$ and $R_F > 0.80$. Rescues models whose temporal correlation is computationally indistinguishable from zero, but whose spectral characteristics closely matches those of GLDAS.

Supplementary Table S1 Summary of CMIP6 models and selection results.

Model	Experiment	Member	Selected	R_T	p	R_F
ACCESS-CM2	Historical, SSP126, SSP245, SSP585	r1i1p1f1	✓	0.270	0.001	0.855
CESM2-WACCM	Historical, SSP126, SSP245, SSP585	r1i1p1f1	✓	0.166	0.046	0.949
CanESM5-CanOE	Historical, SSP126, SSP245, SSP585	r1i1p2f1	✓	0.431	< 0.001	0.926
EC-Earth3	Historical, SSP126, SSP245, SSP585	r1i1p1f1	✓	0.604	< 0.001	0.910
EC-Earth3-CC	Historical, SSP245, SSP585	r1i1p1f1	✓	0.280	0.001	0.890
EC-Earth3-HR	Historical, SSP245	r1i1p1f1	✓	0.242	0.003	0.749
FGOALS-g3	Historical, SSP126, SSP245, SSP585	r1i1p1f1	✓	0.187	0.024	0.807
MIROC-ES2L	Historical, SSP126, SSP245, SSP585	r1i1p1f2	✓	0.426	< 0.001	0.888
MPI-ESM1-2-LR	Historical, SSP126, SSP245, SSP585	r1i1p1f1	✓	0.262	0.002	0.671
MRI-ESM2-0	Historical, SSP126, SSP245, SSP585	r1i1p1f1	✓	0.168	0.044	0.859
IPSL-CM6A-LR	Historical, SSP126, SSP245, SSP585	r1i1p1f1	✓	0.0001	0.999	0.830
IPSL-CM5A2-INCA	Historical, SSP126	r1i1p1f1	✓	0.161	0.053	0.781
UKESM1-0-LL	Historical, SSP126, SSP245, SSP585	r1i1p1f2	-	-0.040	0.631	0.905
ACCESS-ESM1-5	Historical, SSP126, SSP245, SSP585	r1i1p1f1	-	-0.023	0.786	0.447
CAS-ESM2-0	Historical, SSP245, SSP585	r1i1p1f1	-	-0.174	0.036	0.920
CNRM-ESM2-1	Historical, SSP126, SSP245, SSP585	r1i1p1f2	-	-0.044	0.597	0.633
CanESM5	Historical, SSP126, SSP245, SSP585	r1i1p1f1	-	-0.240	0.004	0.880
CanESM5-1	Historical, SSP126, SSP245, SSP585	r1i1p1f1	-	-0.255	0.002	0.802
E3SM-1-0	Historical, SSP585	r1i1p1f1	-	0.035	0.675	0.782
EC-Earth3-Veg-LR	Historical, SSP126, SSP245, SSP585	r1i1p1f1	-	-0.557	< 0.001	0.834
GISS-E2-1-H	Historical, SSP126, SSP245, SSP585	r1i1p1f2	-	-0.090	0.280	0.674
GISS-E2-2-G	Historical, SSP126, SSP245, SSP585	r1i1p3f1	-	-0.173	0.038	0.847
MIROC6	Historical, SSP126, SSP245, SSP585	r1i1p1f1	-	-0.190	0.022	0.816
CESM2	Historical, SSP126, SSP245, SSP585	r4i1p1f1	-	-0.065	0.437	0.832
GFDL-CM4	Historical, SSP245, SSP585	r1i1p1f1	-	-0.035	0.675	0.893

(3) Validation:

A total of 12 models were retained by applying the above criterion to an initial pool of 25 widely used CMIP6 models (see Supplementary Table 1). Their ensemble mean shows a strong correlation with GLDAS during the historical period ($R_T = 0.68$, $p < 10^{-4}$).

(4) Sensitivity analysis:

To demonstrate that the main results are insensitive to the specific threshold values, we repeated the analysis under two alternative threshold combinations. The results are summarized in Supplementary Table 4.

Supplementary Table S4 Sensitivity of model selection results to threshold choice.

**Configuration**	**Tier 1**	**Tier 2**	***n***	**hist R_T**
Main (used in paper)	$R_T > 0,\ p < 0.10$	$\lvert R_T \rvert < 0.01,\ R_F > 0.80$	12	0.684
Sens-1 (relaxed Tier 2: $\lvert R_T \rvert < 0.10$)	$R_T > 0,\ p < 0.10$	$\lvert R_T \rvert < 0.10,\ R_F > 0.80$	15	0.655
Sens-2 (strict Tier 1: $p < 0.01$)	$R_T > 0,\ p < 0.01$	$\lvert R_T \rvert < 0.01,\ R_F > 0.80$	8	0.679

Here, n denotes the number of models retained under each threshold configuration, and hist $\mathbf{R_T}$ denotes the Pearson correlation between the historical ensemble-mean S_{TP} of each alternative model ensemble and that of GLDAS. All historical correlations exceed 0.65, reflecting that the overall shape of the S_{TP} curve did not change substantially, indicating that the model selection results are insensitive to the specific threshold choices.

We additionally repeated the future projection analysis in the main text using the CMIP6 models selected under the two alternative thresholds. As shown in Supplementary Fig. 22, all results remains robust under different CMIP6 model selections. Specifically, both model ensembles exhibit a projected increase in S_{TP} together with the same dominant soil moisture dipole pattern (U_1) (Supplementary Figs. 22a, b, c, d). Under the high-emission scenario (SSP585), both analyses also identify a common breakpoint around 2052 in the temporal evolution (V_1) and the interregional soil moisture difference (δ_{SM}) (Supplementary Figs. 22e, f). After the breakpoint, V_1 exhibits a significantly steeper downward trend, whereas δ_{SM} changes from no significant trend to a significant increase. These features are highly consistent with the corresponding results presented in Fig. 5, providing further evidence that our conclusions are insensitive to the choice of threshold. Moreover, *IPSL-CM6A-LR* model is retained in all three configurations and contributes the distinctive abrupt-regime-shift trajectory under SSP585 (Fig. 5d) discussed in the main text.

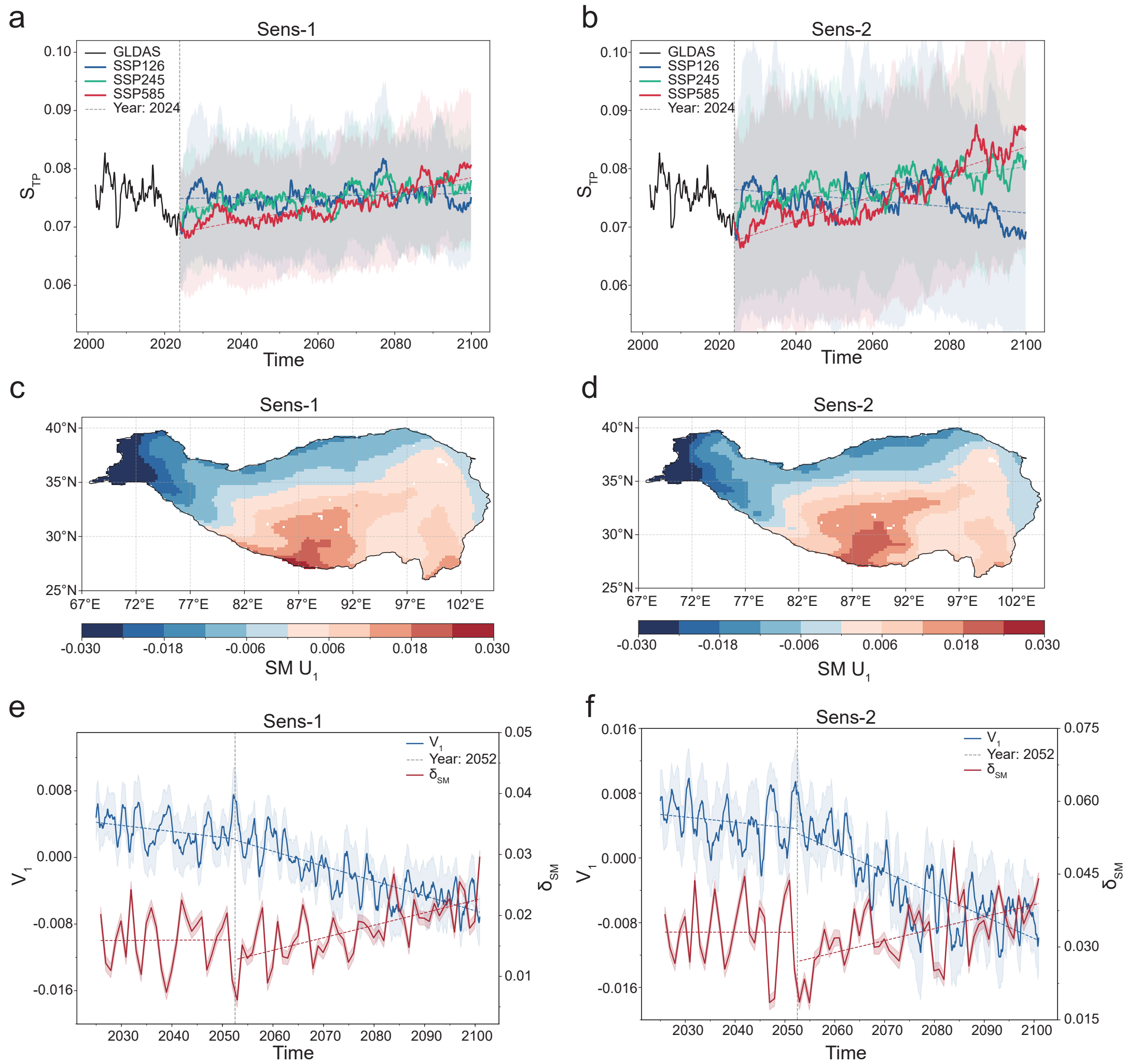

Supplementary Figure S22: Robustness of projected changes in the TP soil moisture system to the model selection threshold. (**a**) and (**b**) Same analysis as Fig. 5a in the main text, but from CMIP6 models selected under Sens-1 and Sens-2, respectively. The high emission scenarios exhibit consistently significant upward trends ($p < 0.001$), indicating a transition toward a more disordered and unstable regime. (**c**) and (**d**) Same analysis as Fig. 5b in the main text, showing the same dipole mode. (**e**) and (**f**) Same analysis as Fig. 5c in the main text. A common breakpoint is identified in 2052 (grey dashed line) by segmented linear regression in both sensitivity experiments. After the breakpoint, V_1 exhibits a significant downward trend ($p < 0.001$), with a slope more than twice that before the breakpoint. In contrast, δ_{SM} shows no significant trend before the breakpoint but increases significantly thereafter ($p < 0.001$).

To address the reviewers' comments, the following text has been added to the *Methods* and *Supplementary Note S1*.

"We selected CMIP6 models for future projection analysis using a two-tier quantitative criterion based on performance over the historical overlap period (2000–2014). Specifically, the monthly surface soil moisture variable (mrsos) was extracted from each CMIP6 model to compute S_{TP}. Two metrics were computed for each model relative to the GLDAS–NOAH reference: (1) the Pearson temporal correlation R_T between the model's S_{TP} and the GLDAS S_{TP}, and (2) the spectral correlation R_F, defined as the

Pearson correlation between the spectral amplitudes of the two standardised S_{TP} series. Unlike R_T, R_F is insensitive to temporal phase offsets caused by differences in the internal variability chronology between the model and the real world.

Because CMIP6 historical simulations are free-running (uninitialized) [87, 88], phase-scrambling tests (50,000 surrogates preserving the GLDAS power spectral density) confirm that $\sigma(R_T) \approx 0.35$ for this series length, meaning R_T alone cannot reliably distinguish a model with correct characteristic timescales from a random-phase surrogate. We therefore applied a two-tier criterion. Tier 1 retains models with a statistically significant positive temporal correlation ($R_T > 0$, $p < 0.1$, one-tailed, $N = 145$). Tier 2 rescues models whose temporal correlation is computationally zero ($|R_T| < 0.01$) but whose spectral structure closely matches GLDAS ($R_F > 0.80$).

To ensure inter-model comparability, the mrsos outputs from all models were resampled to a common $0.25° \times 0.25°$ grid using bilinear interpolation and converted from mass $\mathrm{kg\ m^{-2}}$ to volumetric units ($\mathrm{m^3\ m^{-3}}$). A final ensemble of 12 models is obtained by applying the above criterion to the initial 25 widely used CMIP6 models (see Supplementary Table 1). The ensemble mean of the selected models achieves $R_T = 0.68$ against GLDAS-NOAH ($p < 10^{-4}$). Results are insensitive to the exact threshold values, as CMIP6 models selected under different threshold configurations consistently reproduce the historical S_{TP} trajectories and yield projected future changes that are highly consistent with those shown in Fig. 5 (see Supplementary Table 4 and Supplementary Fig. 22). To ensure internal consistency across hydroclimatic variables, other relevant outputs–including near-surface air temperature (tas), surface snow water equivalent (snw), surface snowmelt flux (snm), and total evaporation flux (evspsbl)–were extracted concurrently from the same final ensemble of 12 models. Detailed descriptions of the model selection procedure are provided in Supplementary Note S1." (Methods, Line 520)

Supplementary Note S1, Page 26:

Supplementary Note S1 CMIP6 Model Selection

S1.1 Phase-scrambling null distribution

CMIP6 historical simulations are uninitialized free-running experiments [2, 3]. Their internal variability chronology (e.g., timing of ENSO events) is independent of the observed record. To quantify the resulting uncertainty in the Pearson temporal correlation R_T, we generated 50,000 phase-scrambled surrogates of the GLDAS S_{TP} series by randomising the Fourier phases while preserving the power spectral density. As shown in Supplementary Fig. 21, for $N = 145$ monthly steps with the observed autocorrelation structure, this yields $\sigma(R_T) \approx 0.35$ and 5th/95th percentiles of 0.56/+0.56. A model with correct spectral structure but random ENSO phase will produce $|R_T| < 0.56$ in 90% of realisations, confirming that R_T alone is an unreliable selection metric at this record length.

S1.2 Spectral correlation R_F

The spectral correlation R_F is defined as the Pearson correlation coefficient between the spectral amplitudes of the two standardised (zero-mean, unit-variance) S_{TP} series. R_F measures whether the model reproduces the dominant variability periods of S_{TP} irrespective of temporal phase.

S1.3 Threshold justification

The thresholds used in the two-tier model selection framework are justified as follows:

- ***Tier 1 threshold (**$p < 0.10$**):** Standard one-tailed significance level used to identify models with statistically significant positive temporal agreement.*
- ***Tier 2 thresholds (**$|R_T| < 0.01$ **and** $R_F > 0.80$**):** Identify models with effectively zero temporal correlation but strong spectral consistency relative to GLDAS, ensuring retention of models that reproduce the observed frequency structure despite phase mismatch.*

S1.4 Sensitivity of results to threshold choice

To verify robustness, the analysis was repeated under two alternative threshold combinations, as summarized in Table S4.

Here, n denotes the number of models retained under each threshold configuration, and hist R_T denotes the Pearson correlation between the historical ensemble-mean S_{TP} of each alternative model ensemble and that of GLDAS. All historical correlations exceed 0.65, indicating that the model selection results are insensitive to the specific threshold choices. Moreover, projected changes in the TP soil moisture system based on CMIP6 models selected under different threshold configurations are highly consistent with the results shown in Fig. 5, including the projected increase in S_{TP}, the dominant soil moisture dipole pattern, and the breakpoint around 2052 in the temporal evolution of V_1 and δ_{SM} (Supplementary Fig. 22).

(4) The coarse resolution of GLDAS-NOAH (0.25°) may smooth out fine-scale heterogeneity, especially in steep terrain in TP. Please add more discussions.

Response: We thank the reviewer for this important comment. We agree that the $0.25° \times 0.25°$ spatial resolution of the GLDAS-NOAH dataset may smooth out fine-scale hydroclimatic heterogeneity, particularly across the complex and steep terrains of the TP. Following the reviewer's suggestion, we have expanded the discussion in the revised manuscript to address this scale dependence.

A coarse resolution may reduce the representation of localized soil moisture gradients and sub-grid variability, which could influence the absolute magnitude of the estimated entropy. We emphasize, however, that our study concerns the temporal evolution and large-scale spatial organization of entropy, i.e, its long-term trend, interannual variability, and ENSO teleconnection, rather than its absolute value. A resolution-dependent offset in the entropy level therefore does not bear directly on our conclusions, provided the entropy evolution itself is robust to resolution. This is exactly what we find: repeating the analysis with a much finer 500 m remote-sensing dataset yields a consistent entropy evolution (Supplementary Fig. 5), confirming that the diagnosed dynamics are not an artifact of the native GLDAS-NOAH grid. In addition, the dominant dipole pattern and its ENSO teleconnection manifest at spatial scales that substantially transcend the native resolution, reflecting a coherent, system-level mode rather than local-scale variability.

The corresponding revision in the Discussion is shown below:

"The relatively coarse resolution of GLDAS-NOAH ($0.25° \times 0.25°$) may further dampen localized SM gradients and sub-grid structure across steep elevation gradients and heterogeneous land surfaces [81,

82], which could influence the absolute magnitude of the estimated entropy. Because our analysis targets the temporal evolution and large-scale spatial organization of entropy rather than its absolute value, such smoothing does not materially affect the dynamics on which our conclusions rest. Consistent with this, the SM entropy evolution remains stable across independent satellite products at substantially finer resolutions ($0.25°$ *and* 500 m*, Supplementary Fig. 5), and the dominant dipole and its ENSO linkage emerge at spatial scales far larger than the native grid, reflecting a collective, system-level mode rather than local-scale variability. Integrating higher-resolution observations and improved cold-region land-surface simulations would further enable the entropy framework to resolve finer-scale hydroclimatic heterogeneity across the TP."* (Discussion and Conclusion, Line 463)

References:

1. Heginbottom, J. A., Brown, J., Melnikov, E. S., et al. Circum-arctic map of permafrost and ground-ice conditions, version 2 (2002). https://nsidc.org/data/GGD318/versions/2

2. Cui, Y. K., Zeng, C., Zhou, J., et al. A spatio-temporal continuous soil moisture dataset over the Tibet Plateau from 2002 to 2015. Sci. Data 6, 247 (2019).

3. Gao, X., Lu, P. & Yi, Y. H. High-resolution daily surface soil moisture mapping over the Qinghai–Tibet Plateau via predictors fusion and machine learning. J. Hydrol. 668, 134982 (2026).

4. Lesk, C. S. & Mankin, J. S. More concentrated precipitation decreases terrestrial water storage. Nature 653, 425–432 (2026).

5. Zhang, W. Q., Liu, L., & Li, X. P., et al. Intensified response of extreme precipitation to rising temperature over the Tibetan Plateau from CMIP6 multi-model ensembles. J. Hydrol. 637, 131397 (2024).

6. Huss, M., & Hock, R. Global-scale hydrological response to future glacier mass loss. Nat. Clim. Change 8, 135–140 (2018).

7. Deser, C., Knutti, R., Solomon, S., et al. Communication of the role of natural variability in future North American climate. Nat. Clim. Change 2, 775–779 (2012).

8. Eyring, V., Bony, S., Meehl, G. A., et al. Overview of the Coupled Model Intercomparison Project Phase 6 (CMIP6) experimental design and organization. Geosci. Model Dev. 9, 1937–1958 (2016).

Reviewer #2:

As one of the climate sensitive regions, the destabilization of Tibetan Plateau would have potentially profound effects on global climate. Soil moisture dynamics, one perspective of stability of Tibetan Plateau, remain poorly quantified especially regarding its quantification and its change with ENSO events. The authors implemented the concept of entropy of soil moisture to diagnose the disorder and internal heterogeneity of the Tibetan Plateau system. Results suggested that the declining entropy of soil moisture indicates an increasing ordered state of the system over the period 2000-2024. The spatial-temporal patterns of the entropy of soil moisture are significantly correlated with ENSO events. The future warming trend especially under SSP585, however, would drive a transition from an ordered to a more disordered state, underscoring substantial risks for climate feedback and water security. In general, the paper is clearly written, and the results are very interesting. However, there are still two major limitations that need to be addressed before publication.

Response: We sincerely thank the reviewer for the positive and constructive assessment, and for recognizing the clarity of the manuscript and the significance of the entropy-based framework. Following the reviewer's suggestions, we have thoroughly revised the manuscript, including restructuring the *Introduction*, expanding the *Discussion and Conclusion* to cover climate implications, refining the figures and captions, and adding the rationale and sensitivity analyses for the smoothing windows. Detailed point-by-point responses follow below.

1. The introduction is not well written.
The importance of Tibetan Plateau is mentioned extensively while the selling points of this paper are not well described especially in the first paragraph. For instance, how has the stability of Tibetan Plateau been studied before? What has been previously reported regarding the impacts of ENSO events on the Tibetan Plateau? Why would the entropy of soil moisture be chosen instead of other parameters, such as precipitation and temperature? In addition, the last paragraph of Introduction shall be what will be investigated in this study instead of conclusions.

Response: We thank the reviewer for this valuable comment. In response, we have substantially revised the *Introduction* to emphasize the scientific motivation, novelty, and study framework. Specifically, we expanded the discussion of previous studies on TP stability, provided a more comprehensive overview of the reported impacts of ENSO on the TP, clarified the rationale for selecting soil moisture as the focus of this study, and revised the final paragraph to more clearly describe the scope and content of the work. The specific revisions are as follows:

(1) To address reviewer's comment that "how has the 'stability' of the TP been studied before?", we have incorporated relevant previous studies on TP stability and highlighted their limitations in the revised manuscript. Specifically, in the first paragraph of the *Introduction*, we clarify that recent work has identified early warning signs of instability in various TP subsystems, suggesting that the region may be approaching critical thresholds. However, these studies are largely based on local, site-specific analyses and provide limited insight into system-wide spatial organization, leaving the overall stability of the TP hydrological system uncertain.

We also summarize the current stability analysis of the TP main based on critical slowing down (CSD)

theory, which uses early-warning indicators such as variance, lag-1 autocorrelation, and estimated recovery rate. While CSD has revealed instability in snow and vegetation subsystems over the TP, it has limitations, including reliance on idealized assumptions and potential biases from empirical data. More importantly, existing CSD indicators primarily characterize local temporal dynamics and provide limited insight into system-level spatial interactions and organization. To address these gaps, we apply Eigen Microstate Entropy as a system-level metric to quantify soil moisture system disorder and assess its implications for stability under both historical variability and projected climate warming. The specific revisions are shown below:

"Evaluating this potential tipping behavior is therefore critical, yet existing assessments remain limited. Current early-warning analyses of the TP, which have identified such destabilization within individual subsystems such as snow cover and vegetation [12, 15–18], assess stability largely on a local, location-by-location basis and reveal little about how the system is organized in space, leaving unresolved whether the Plateau's hydrological system as a whole is gaining or losing stability." (Introduction, Line 56)

"Linking these spatial structures to system stability, however, demands a perspective that prevailing resilience diagnostics do not provide. To date, stability loss in such systems has been detected largely through statistical early-warning indicators grounded in critical slowing down (CSD) theory, principally rising variance and lag-1 autocorrelation, or a declining recovery rate, which signal a decreasing capacity to recover from perturbations. Applied to the TP, these indicators reveal a persistent loss of stability in snow-cover extent since around 2008 [12] and a parallel decline in vegetation resilience [15]. These approaches, however, share key limitations. CSD-based diagnostics rest on idealized assumptions, including local linearity and quasi-stationarity, that are frequently violated in practice [11], and they remain sensitive to data quality issues that can bias the inferred resilience [43, 44]. More fundamentally, they characterize the temporal dynamics of individual locations, but not the spatial organization of a key state variable across the system, the property that governs the systemic stability of the TP as a whole." (Introduction, Line 100)

(2) Regarding why the entropy of soil moisture is chosen instead of other parameters, we have substantially expanded the second paragraph of the *Introduction* to clarify the rationale for selecting SM as the focus of this study. Specifically, we now emphasize that SM is a key state variable of the TP hydrological system, regulating hydrological, ecological, and biogeochemical processes while mediating land–atmosphere interactions. SM anomalies on TP can also influence regional and remote climate variability through their impacts on evapotranspiration, surface energy partitioning, monsoon evolution, and large-scale atmospheric teleconnections. Importantly, we now explicitly highlight SMs role as an integrating state variable, accumulating the combined effects of precipitation, temperature, evapotranspiration, and cryospheric processes (e.g., snowmelt and freeze–thaw cycles), thereby reflecting the net hydrological state of the land surface. The relatively long memory of SM enables it to retain the imprint of past perturbations and represent the system's integrated response, making it more suitable than individual climatic drivers for diagnosing the spatial organization and stability of the TP hydrological system. The revised paragraph from the *Introduction* is presented below:

"Soil moisture (SM) acts as a critical state variable of the TPs hydrological system, regulating surface runoff [19, 20], groundwater recharge [21], vegetation dynamics [22] and carbon fluxes [23], thereby

underpinning ecosystem resilience and regional water security. Through its influence on evapotranspiration (ET) and the partitioning of surface heat fluxes, SM also governs land–atmosphere exchanges of water and energy, and influences both regional and remote climate variability and feedbacks [23, 24]. Specifically, SM anomalies over the TP modulate regional thermal forcing through land–atmosphere interactions, contributing to heat extremes that range from high-altitude heatwaves on the Plateau itself to teleconnected heat events over Europe and East Asia via Rossby wave propagation [25–27]. They also exert hydrological influences beyond the Plateau: wetter spring soil conditions favor an earlier onset of the South Asian summer monsoon and provide predictive information for summer precipitation and flood risk in the Yangtze River basin [28, 29]. These widespread impacts imply that changes in the variability and spatial organization of the TP soil moisture system could have far-reaching consequences for both the Plateau and downstream regions. Rather than acting as an isolated climatic driver, SM over the TP serves as an integrating state, accumulating the combined influence of precipitation, temperature, evapotranspiration, and cryospheric processes such as snowmelt and freeze–thaw cycles [27, 30, 31]. Its relatively long memory allows perturbations to persist rather than dissipate instantaneously, reflecting the net hydrological state of the land surface [32]. Unlike precipitation or temperature, which capture only partial aspects of the water and energy balance, SM records the system's integrated response. Particularly in the highly heterogeneous and sensitive environment of the TP, this makes SM the natural variable for diagnosing the spatial organization and stability of the TP's hydrological system." (Introduction, Line 62)

(3) As for what has been previously reported regarding the impacts of ENSO on the TP, we have provided a more comprehensive overview in the third paragraph of the *Introduction*. Specifically, we now summarize evidence showing that ENSO influences TP hydroclimate through large-scale atmospheric teleconnections, affecting precipitation, snowfall, snowpack accumulation, terrestrial water storage, and lake-level variability across the Plateau. However, we further emphasize that, despite these documented impacts on multiple components of the TP water cycle, its influence on the variability and stability of the TP soil moisture system remains insufficiently quantified. The revised paragraph is reproduced below:

"The El Niño–Southern Oscillation (ENSO), for example, exerts a strong influence on the TP hydroclimate through large-scale atmospheric teleconnections that alter regional circulation and moisture transport [37]. It has been linked to substantial variations in precipitation and snow conditions across the TP, including winter snowfall over the western TP [38], summer rainfall over its southwestern sector [39], and snowpack accumulation that may persist into subsequent seasons [40]. These hydroclimatic perturbations can propagate into terrestrial water storage, reflected by pronounced lake-level shrinkage during major El Niño events, followed by widespread lake expansion in subsequent years [41, 42]. Despite these documented impacts on multiple components of the TP water cycle, whether and how ENSO shapes the variability and large-scale organization of the TP soil moisture system remains poorly understood." (Introduction, Line 89)

(4) Finally, in response to reviewer's comment that the last paragraph of the Introduction should describe what will be investigated rather than the conclusions, we have rewritten the final paragraph to focus on the study's objectives and framework. The revised paragraph is reproduced below:

"In this work, we apply this entropy-based diagnostic framework to quantify the spatiotemporal disorder

and systemic stability of the SM over the TP. Specifically, we first identify the most suitable SM dataset for TP-wide analysis through a comprehensive evaluation of GLDAS-NOAH, ERA5-Land, and MERRA-2 against in situ observations, considering both value agreement and dynamical consistency. Using the selected GLDAS-NOAH dataset, we employ soil moisture entropy to characterize the long-term evolution and interannual variability of system disorder across the TP during 2000–2024, and examine its relationship with ENSO. Finally, we use the Coupled Model Intercomparison Project Phase 6 (CMIP6) simulations to evaluate the future evolution of soil moisture system disorder under continued warming, thereby assessing whether the recent gain in stability is likely to persist." (Introduction, Line 125)

2.The discussion section does not address implications so well.
For example, the authors mentioned the distinct dipole (western negative phases-wetter regions vs southwestern positive phases-drier regions) and cited previous study that negative phase in western regions would cast impacts on the Yangtze River Basin. Then what will the positive phase in southern region impact? What is the new knowledge that the results would contribute to, when we understand ENSO would impact precipitation anomalies and soil moisture? The reason why this question is raised is because the impacts of ENSO events on the Tibetan Plateau has been studied.

Response: We thank the reviewer for this valuable comment. We agree that the original *Discussion and Conclusion* section did not sufficiently elaborate on the broader implications of the identified soil moisture dipole pattern and did not clearly articulate the novel insights of our study beyond previous ENSO-related investigations on the TP. In response, we have fully revised and expanded the *Discussion and Conclusion* section accordingly.

In the revised *Discussion and Conclusion* section, we have expanded the discussion of both branches of the dipole pattern. Specifically, we highlight that persistent drying in the southwestern TP may exacerbate vegetation degradation [1], enhance land surface warming through positive soil moisture–temperature feedbacks [2], and influence regional atmospheric circulation and moisture transport [3]. These effects may further contribute to hydroclimatic extremes within and beyond the TP. The following discussion has been added to the revised manuscript:

"Beyond this overall rise in disorder, our results reveal how the underlying spatial structure of the SM system reorganizes as entropy increases. The projected entropy rise is accompanied by an amplification of the dipole mode marked by strengthening west–southwest contrasts, indicating increasing spatial heterogeneity within the TP hydroclimatic system (Fig. 5b, Supplementary Fig. 15). Such enhanced heterogeneity is expected to exacerbate uneven water availability and intensify compound extremes, with cascading impacts on major downstream river basins in South and East Asia that collectively support nearly two billion people [1, 2]. For example, spring SM anomalies in the western TP have been linked to extreme summer precipitation in the Yangtze River Basin, potentially intensifying downstream flood risk [29]. In the southwestern TP, SM deficits not only exacerbate vegetation degradation [22] but also induce a positive land surface temperature feedback that contributes to regional warming [77], and may further modify large-scale circulation by enhancing moisture transport toward the northern TP and raising the likelihood of extreme precipitation [78]. These contrasting impacts of the two branches underscore that future assessments of TP hydroclimatic stability should move beyond total water availability and treat spatial heterogeneity as a key dimension." (Discussion and Conclusion, Line 400)

On the question of novelty, prompted by the reviewer's insightful comments, we have more explicitly stated what new understanding our results offer in the revised version. The reviewer is right that ENSO's influence on the TP climate field has itself been studied. Our contribution is not to re-document that influence, but to show what it implies for the system as a whole: ENSO governs not merely the magnitude of TP soil moisture, but the soil moisture systems degree of organization, order-versus-disorder state, and hence stability. In the revised manuscript, we make this new knowledge explicit along two lines:

First, we find that the ENSO and TP soil moisture systems are coupled at the system level, expressed as a co-fluctuation of their respective disorder: the entropy of the TP soil moisture system lags that of ENSO by about six months ($r = 0.59$, $p < 0.001$). This shows that a known driver acts on a previously unmeasured property, the system's internal organization, rather than only on local anomalies. The consistent lead of S_{ENSO} further indicates that the ordered/disordered state of the TP soil moisture system carries a degree of predictability from tropical Pacific variability.

Second, we identify how ENSO sets this disorder: through cross-regional spatial heterogeneity rather than basin-wide anomalies. ENSO imprints a west–southwest dipole on TP soil moisture, and it is the strength of this inter-regional contrast that governs the structural disorder of the system. This reveals that the organizational state, and therefore the stability, of the TP soil moisture system is controlled by spatial contrasts across the Plateau, a system-level mechanism that local, sub-region analyses cannot capture. Together, these two findings reframe the ENSO–TP relationship from a collection of local anomaly responses into a single, system-level coupling.

To clarify these points, we have expanded the *Discussion and Conclusion* section as follows:

"Pronounced warming across the TP is driving substantial changes in regional hydrology, raising concerns over the stability of the "Asian Water Tower" [2]. Beyond local impacts, the TP is embedded in a network of teleconnections among potential climate tipping elements, including ENSO, the South Asian monsoon, and even the Amazon rainforest [10, 12]. Destabilization in one component can trigger cascading responses in others, thereby amplifying systemic risks [67]. Our results provide new evidence for such coupling: SM entropy on the TP is modulated by ENSO, as evidenced by the six-month lagged correlation between S_{TP} and S_{ENSO} (Fig. 2d). We further trace the mechanism underlying this coupling: ENSO-induced precipitation anomalies imprint a west–southwest dipole on TP SM (Fig. 3a, Fig. 4a), and the strength of this inter-regional contrast governs the structural disorder of the SM system, as captured by the significant correlation between δ_{SM} and S_{TP} (Fig. 3a). The entropy framework is central to uncovering this chain. Conventional ENSO–TP assessments quantify hydroclimatic anomalies within individual sub-regions or seasons, such as developing-ENSO effects on southwestern TP summer rainfall [39] and moisture sources for winter precipitation over the western Plateau [38], focusing on the mean state or amplitude of local variability rather than how variability is organized across the Plateau. Such metrics cannot, by construction, represent how variability is organized across the Plateau as a whole. By measuring the degree of disorder in the spatiotemporal organization of the SM field, our entropy-based diagnostic instead resolves two features that these approaches miss: a system-level co-fluctuation of disorder between the ENSO and TP soil moisture systems, and the controlling role of cross-regional spatial heterogeneity in setting that disorder. This reframes the ENSO–TP relationship from a collection of local anomaly responses into a single, system-level coupling, and reinforces the view

that the TP interacts dynamically with other global climate components, so that instabilities on the Plateau may trigger large-scale propagation through teleconnections [3, 6, 12].” (Discussion and Conclusion, Line 351)

Minor comments:

1. Is it possible to use the same colorbar for Fig.3a and Fig.5d so that the amplification of soil moisture entropy would be more explicit?

Response: Thank you for this suggestion. Fig. 3a and Fig. 5d have been revised to use a unified color scale, enabling direct comparison and more clearly highlighting the amplification of soil moisture entropy. The revised figures are shown below.

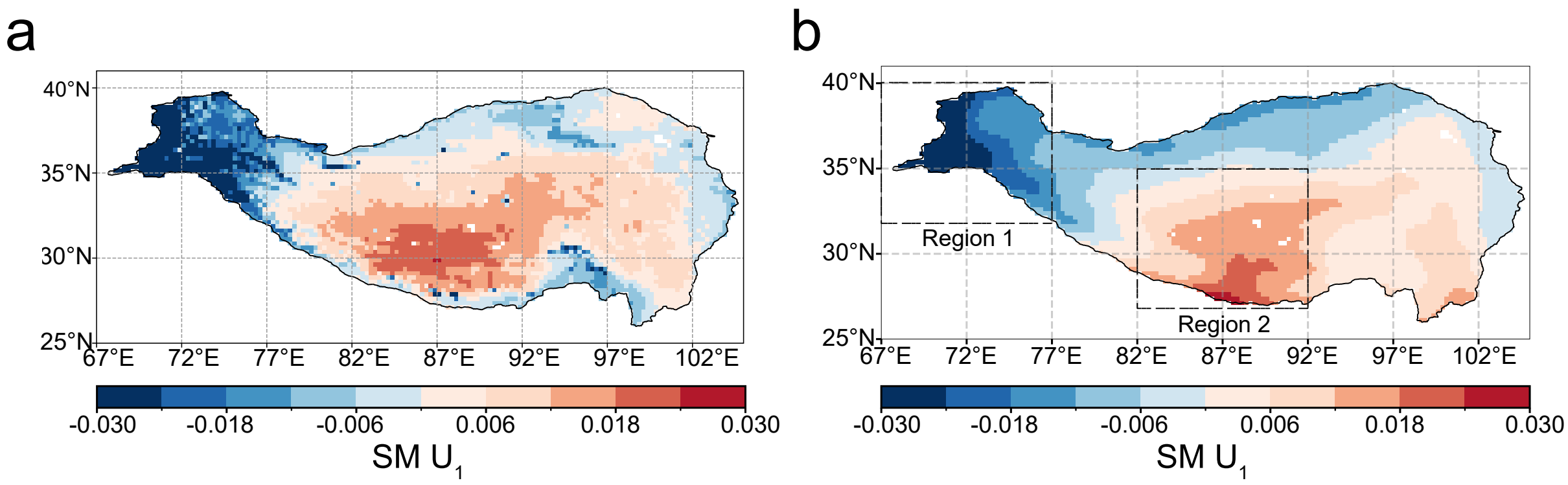

Figure R1: Revised Fig. 3a (a) and Fig. 5d (b) using a unified color scale.

2. Figure caption in Fig.5: “ensemble-mean... of soil moisture mode (U_1) under SSP585”, which ensemble? Is the mean of c_1–c_4?

Response: We thank the reviewer for catching this ambiguity. The term “ensemble mean” here refers to the **multi-model** mean and is computed in two steps: we first apply the eigen-microstate decomposition to each of the 12 selected CMIP6 model separately, obtaining the leading soil-moisture mode (U_1) per model under SSP585. We then average these 12 model-specific U_1 patterns to obtain the ensemble-mean pattern shown in Fig.5. It is therefore **not** the mean of the four leading eigen-microstate contributions to S_{TP} (c_1–c_4) shown in the adjacent panel of the original figure. We recognise that placing the c_1–c_4 contribution panel next to the U_1 panel was the likely source of confusion. In the revised figure, the c_1–c_4 contribution panel has been moved to Supplementary Fig. 15, and the caption now states this procedure explicitly:

"***Fig. 5***... ***(b)*** ~~*Ensemble-mean spatial pattern of the dominant soil moisture mode (U_1)*~~ *Multi-model ensemble mean of the leading soil-moisture eigen-microstate (U_1) under SSP585, obtained by averaging the individual U_1 patterns across the 12 selected CMIP6 models. Rectangles define the key dipole centers: Region 1 (western TP, negative phase) and Region 2 (southwestern TP, positive phase).*"

3. The lag of six months between S_{ENSO} and S_{TP} is later not used in the smoothing window analysis. Instead, three-year or two month smoothing window were used for comparison. The choices of the

sizes of smoothing window seem to be very subjectively, any support to the choices?

Response: We thank the reviewer for this comment, which made us realize that the original manuscript did not clearly separate the roles of three distinct numbers, the six-month lag, the 3-year window, and the 2-month window, giving the impression that they should be mutually consistent or derived from one another. We clarify the distinction below and have added a dedicated Supplementary Note S3 documenting every window choice and its sensitivity analysis.

(1) On the six-month lag and why it is not applied as a window/alignment parameter:

The six-month lag and the smoothing windows answer different questions and are not interchangeable. The six-month lag is a lead–lag offset between two entropy time series: it characterizes how the disorder of the TP soil-moisture system (S_{TP}) follows that of the ENSO system (S_{ENSO}) at the system level (Fig. 2d). The smoothing windows (3-year and 2-month), by contrast, are *estimation and aggregation windows* applied when constructing each series *before* any comparison: the 3-year window is the sampling window required for a stable entropy/spectrum estimate, and the 2-month aggregation matches the overlapping bi-monthly definition of MEI.v2. A window length is therefore not a temporal offset, and selecting it is independent of, and not a substitute for, the lead–lag offset. The lag is thus applied exactly where it is defined (the S_{ENSO}–S_{TP} lagged correlation, Fig. 2d) and is not omitted from the window analysis. The two simply operate at different stages.

We would further clarify why the lag is physically meaningful for S_{TP}–S_{ENSO} but should not be imposed on V_1–MEI.v2 or δ_{SM}. It follows directly from the different mathematical nature of the two diagnostics within the Eigen Microstate framework. The leading temporal mode V_1 is a first-order, instantaneous indicator: by construction ($\boldsymbol{A} = \sum_I \sigma_I \boldsymbol{U}_I \otimes \boldsymbol{V}_I$), $V_1(t)$ is the linear projection of the soil-moisture field onto the dominant spatial pattern U_1 at each time step. It therefore tracks the direct, precipitation-mediated imprint of ENSO. Because surface SM responds to precipitation at an $\sim$1-month lag (Fig. 4b, $r = 0.86$) and precipitation responds to ENSO promptly, V_1 is essentially contemporaneous with ENSO (the $\sim$1-month delay being absorbed by the 12-month smoothing). Thus, no time lag was used in the V_1–MEI.v2 analysis, which is the physically expected result.

Entropy, by contrast, is a higher-order, window-integrated functional of the entire eigenvalue spectrum, $S_{\text{EM}} = -\sum_I p_I \ln p_I$, estimated over a multi-year window. It does not measure the response to any single event. It measures how the organization of the whole field is redistributed across modes. This quantity is inherently cumulative and low-pass: the reorganization of the field lags the forcing because it integrates snowmelt, freeze–thaw memory, and spatially asynchronous seasonal anomalies (e.g. during El Niño, suppressed southwestern summer rainfall and enhanced western winter snowfall are accumulated over seasons), extending the entropy-level response to $\sim$6 months. The six-month lag is therefore an emergent property of the entropy coupling, generated by the windowed-entropy operation itself, and it is not a property of the instantaneous field.

Consequently, transferring this lag to the prompt, mode-level comparisons would mix two different timescales and is physically inappropriate. In fact, doing so weakens rather than strengthens the correspondence. We verified this explicitly: re-deriving the V_1–temperature spatial correlation with the six-month lag still recovers the canonical ENSO pattern over the tropical Pacific, but with a somewhat weaker pattern amplitude than the contemporaneous (lag-free) case (Fig. R2a, b). This is exactly

what the argument above predicts: because V_1 is an instantaneous descriptor, offsetting it from a contemporaneous ENSO index degrades the match, whereas the contemporaneous comparison maximizes it. The lagged result therefore confirms both that the ENSO–EM1 link is robust (the pattern persists either way) and that the contemporaneous comparison is the physically appropriate choice for a mode-level quantity. The six-month lag belongs to the integrated, entropy-level coupling, not to the instantaneous mode. We regard the coexistence of a prompt mode-level response (V_1, ∼0–1 month) and a delayed system-level response (S_{TP}, ∼6 months) as a physically coherent result in its own right: it demonstrates that the lag is produced by the integrative, entropy-level reorganization of the field rather than by the response time of the raw soil-moisture signal. We apply the lag where it is physically meaningful and use contemporaneous comparisons for the prompt spatial-contrast analyses.

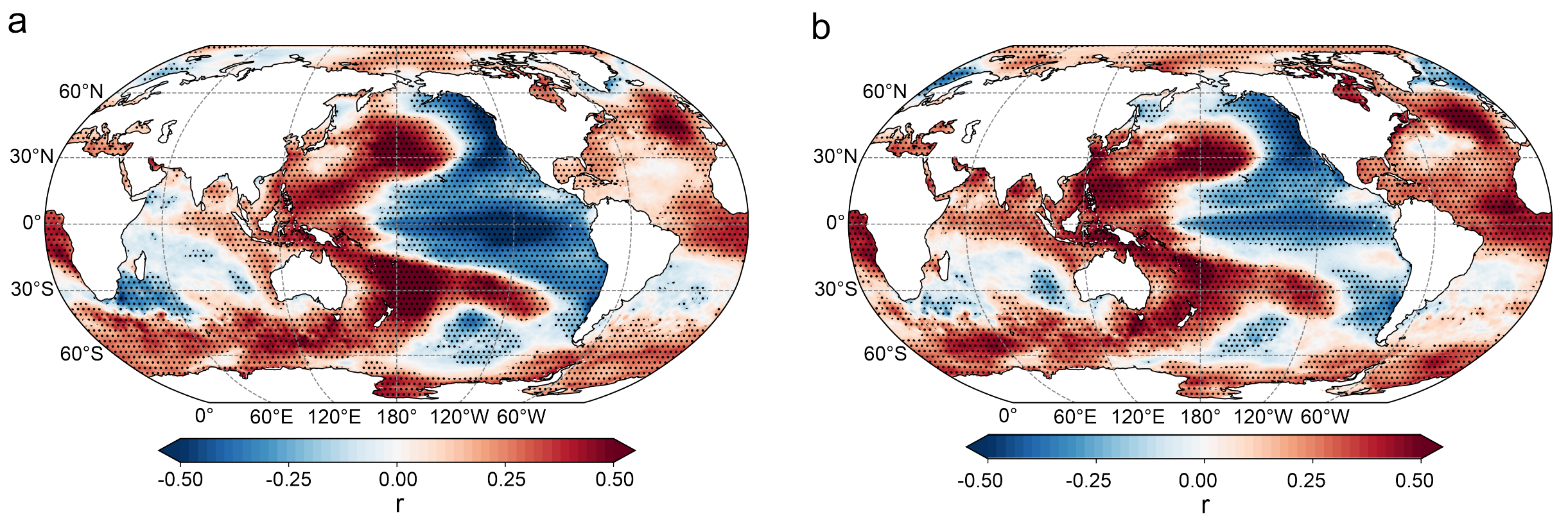

Figure R2: Spatial correlation maps between the temporal evolution of the soil moisture dipole mode (V_1) and global ocean surface air temperature. (**a**) Contemporaneous (lag-free) case. (**b**) With a 6-month lag applied. Both V_1 and temperature time series were smoothed using a 12-month moving average with a 1-month step. Both cases recover the characteristic ENSO-like pattern over the tropical Pacific, but the contemporaneous case (**a**) yields the strongest correlation, whereas applying the 6-month lag (**b**) reproduces the same pattern with reduced amplitude. This is consistent with V_1 being an instantaneous indicator of the soil-moisture field, for which a contemporaneous comparison with ENSO is physically appropriate. The six-month lag instead characterizes the integrated, entropy-level coupling between S_{ENSO} and S_{TP} (Fig. 2d).

(2) 3-year moving window for S_{TP}:

The 3-year moving window used to calculateS_{TP} was selected based on both statistical and physical considerations. First, because S_{TP} is derived from monthly soil moisture anomalies, a 3-year window provides a sufficient sample size to ensure stable and robust estimates of entropy; substantially shorter windows may introduce sampling instability. Second, the 3-year window aligns with the characteristic interannual timescale of ENSO variability, allowing the targeted signal to be resolved while avoiding the blending of multiple ENSO events in longer windows. This choice is further supported by the temporal evolution of the ENSO-related soil moisture dipole mode (V_1), whose dominant periodicities (approximately 2–3.8 years) closely match the selected window timescale.

We additionally performed sensitivity tests using 2- and 4-year windows and found that the resulting S_{TP} series remained highly consistent with the 3-year estimate, with Pearson correlation coefficients

exceeding 0.7 in all cases (Supplementary Fig. 23a). Furthermore, the maximum lag correlations between S_{TP} and S_{ENSO} under the 2- and 4-year windows also remained above 0.5 (Supplementary Fig. 23b), confirming that our main conclusion regarding the temporal coupling between SM entropy and ENSO entropy is insensitive to the choice of window length.

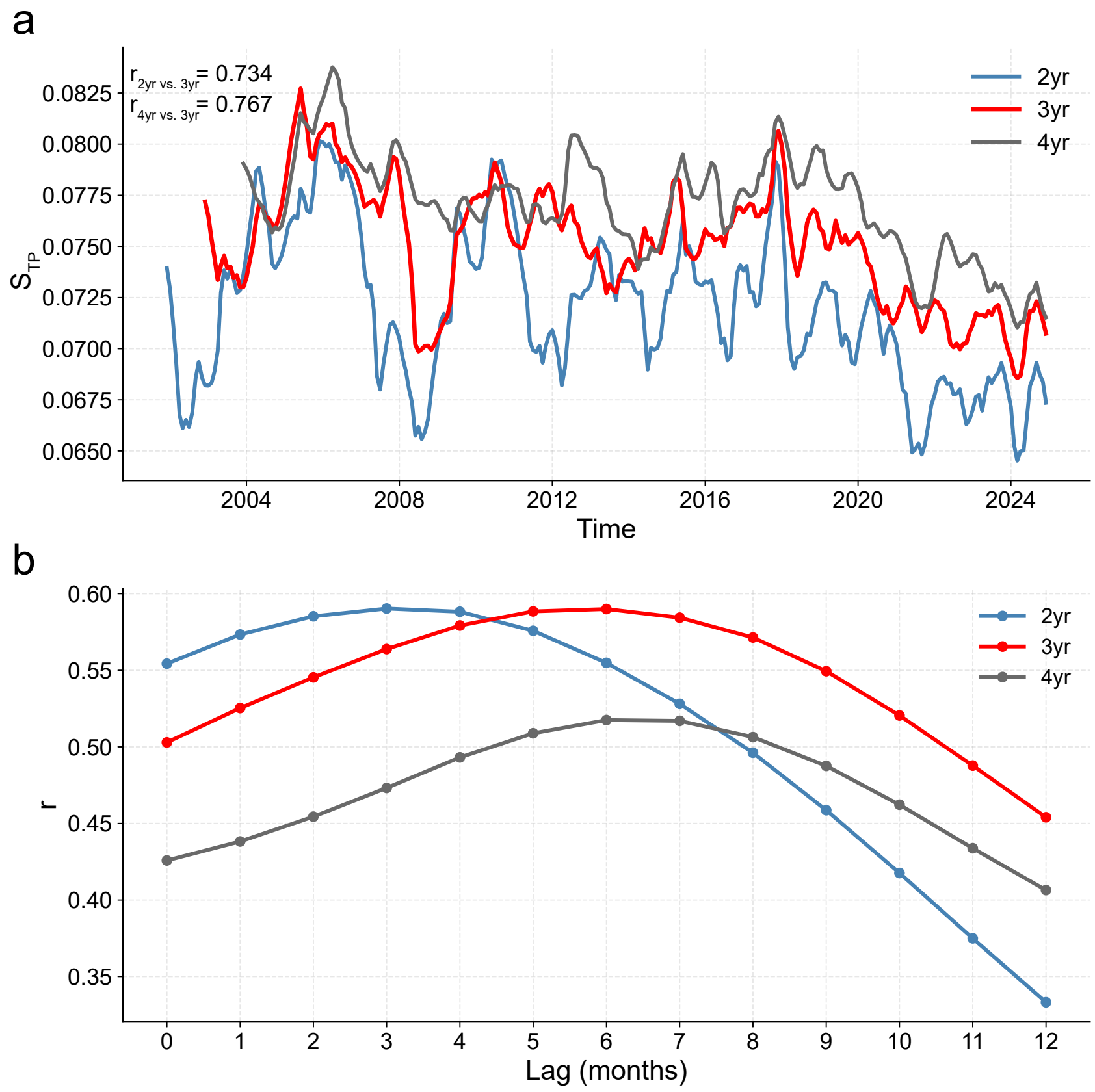

Supplementary Figure S23: Sensitivity analysis of moving-window length for S_{TP} estimation. (**a**) Comparison of S_{TP} derived using 2-, 3-, and 4-year moving windows, showing high consistency with the 3-year estimate. (**b**) Lag correlations between S_{TP} and S_{ENSO} across window lengths, confirming a robust lead–lag relationship.

(3) 3-year moving window for δ_{SM}:

Regarding the choice of the 3-year moving window used for δ_{SM} (Fig. 3c), we note that δ_{SM} is defined as the inter-regional soil moisture difference between regions corresponding to the negative and positive phases of U_1. It is introduced as a mechanistic indicator for variations in S_{TP}, reflecting soil moisture spatial heterogeneity associated with system disorder, such that higher disorder corresponds to greater heterogeneity and vice versa. Since S_{TP} is constructed using a 3-year moving window, δ_{SM} is computed on the same timescale to ensure consistency within the attribution framework.

(4) 2-month moving window for correlation:

In Supplementary Fig. 10, a 2-month temporal aggregation is applied to the monthly soil moisture anomalies in the positive and negative U_1 phase regions (SM U_1^P and SM U_1^N), as well as to their inter-regional difference (δ_{SM}). This was done to match the bi-monthly definition of the MEI.v2 index, which is used here as an indicator of ENSO intensity, thereby enabling a more direct comparison

between soil moisture variability and ENSO evolution. The same temporal aggregation was also adopted in Fig. 3d to ensure consistency in the temporal representation of δ_{SM} when comparing its distributions across different ENSO phases.

We further repeated the analysis using monthly anomalies without temporal smoothing for SM U_1^P, SM U_1^N, and δ_{SM} (Supplementary Fig. 24). The resulting patterns remained highly consistent with those obtained using the 2-month window, confirming that our conclusions are insensitive to the choice of temporal aggregation.

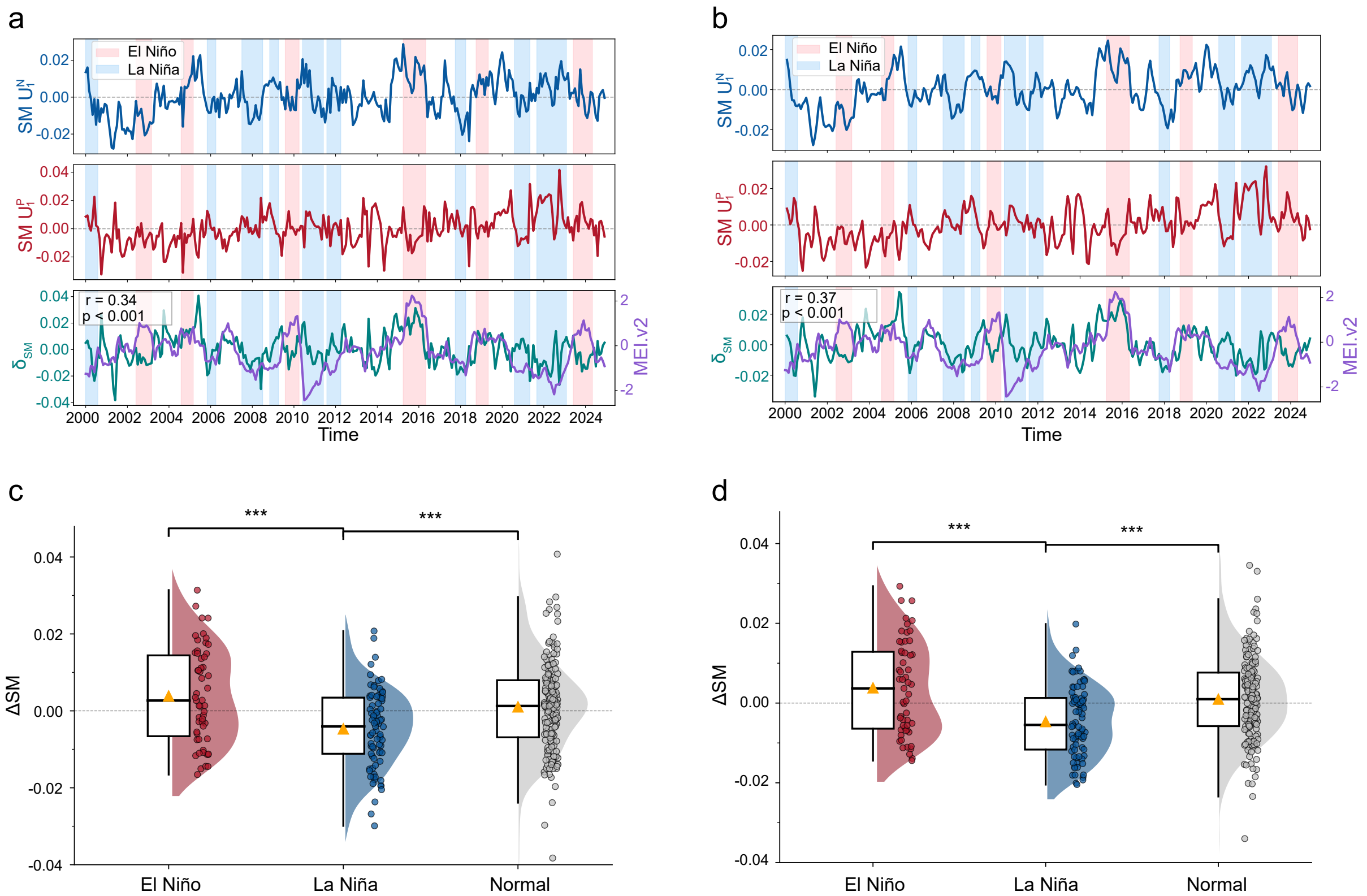

Supplementary Figure S24: Insensitivity of the relationship between SM spatial heterogeneity and ENSO to smoothing window. (**a**) and (**b**) show the SM anomalies in the negative and positive U_1 phase regions (SM U_1^N and SM U_1^P, respectively), together with their inter-regional difference (δ_{SM}) and the MEI.v2 index. Panel (**b**) applies a 2-month running mean to SM-related variables, while panel (**a**) shows monthly values. The correlations between δ_{SM} and MEI.v2 are 0.34 and 0.37, respectively ($p < 0.001$), indicating weak sensitivity to smoothing. (**c**) and (**d**) show violin plots of δ_{SM} based on monthly values and a 2-month running mean, respectively, during El Niño (red), La Niña (blue), and neutral (gray) periods. Boxplots show the median, interquartile range (IQR), whiskers within 1.5×IQR, and mean values (yellow triangles). Significant differences (***, $p < 0.001$) were identified between El Niño and La Niña, and between La Niña and neutral, based on one-way ANOVA and two-sided Mann–Whitney U-test. Both cases consistently show enhanced (reduced) δ_{SM} during El Niño (La Niña), with weak contrasts under neutral conditions.

(5) Updated paper text:

The following text has been added to the Methods and Supplementary Note S3.

"To quantify the temporal evolution of entropy, moving-window analyses were performed using variable-specific parameter settings. For soil moisture entropy (S_{TP}), a 3-year moving window with a 1-month step was adopted. This window provides sufficient samples for robust entropy estimation while preserving the characteristic interannual variability associated with ENSO-related soil moisture dynamics. For ENSO entropy (S_{ENSO}), a 1-year moving window with the same monthly step was used to resolve individual ENSO events while avoiding signal cancellation caused by blending successive opposite-phase ENSO events. The rationale for these window selections and the associated sensitivity analyses are provided in Supplementary Note S3." (Methods, Line 601)

Supplementary Note S3, Page 29:

Supplementary Note S3 Rationale for moving-window selection

The window lengths for each entropy or correlation analysis were not selected independently but followed a common criterion: for each variable, we chose the shortest window that provides sufficient samples to ensure stable estimates while remaining as close as possible to the characteristic timescale of the process being resolved. The resulting differences across variables arise for two reasons: (i) the variables have different temporal resolutions (monthly soil moisture versus daily temperature), and (ii) the variables represent processes operating on different timescales, ranging from ENSO-related interannual variability to the bi-monthly MEI.v2 index. Sensitivity analyses further confirm that the main conclusions are insensitive to reasonable variations in window length.

S3.1 Three-year window for S_{TP}

The 3-year moving window used to calculate soil moisture entropy (S_{TP}) was selected based on both statistical and physical considerations. From a statistical perspective, because S_{TP} is derived from monthly data, a 3-year window provides a sufficient sample size for robust estimation of the underlying spatiotemporal dependence structure; substantially shorter windows may introduce sampling instability into the entropy estimate.

From a physical perspective, a 3-year window is broadly consistent with the characteristic timescale of ENSO variability (typically 2–7 years), allowing the associated interannual soil moisture signal to be resolved without blending multiple ENSO events within a longer averaging window. This choice is further supported by the temporal evolution of the ENSO-related soil moisture dipole mode (V_1), which was identified from the full study-period dataset and exhibits dominant periodicities of approximately 2–3.8 years.

To further evaluate the sensitivity of this choice, we repeated the analysis using alternative window lengths of 2 and 4 years. The resulting S_{TP} time series remained highly consistent with the original 3-year estimate, with Pearson correlation coefficients exceeding 0.7 in both cases (Supplementary Fig. 23a). In addition, the maximum lag correlations between S_{TP} and S_{ENSO} remained above 0.5 for both alternative windows (Supplementary Fig. 23b), confirming that the main conclusions are robust to the choice of window length. However, the 2-year window yields a more fluctuating S_{TP} time series, whereas the 4-year window produces a noticeably smoother evolution. Therefore, the 3-year window provides a balanced compromise between preserving temporal variability and reducing estimation noise.

S3.3 Two-month window for correlation analysis

In Fig. 3 and Supplementary Fig. 10, a 2-month moving average was used to analyze monthly soil moisture anomalies in the positive and negative U_1 phase regions (SM U_1^P *and* SM U_1^N*), as well as their inter-regional difference* (δ_{SM})*. This was done to match the bi-monthly definition of the MEI.v2 index, enabling a more direct comparison between soil moisture variability and ENSO intensity.*

Repeating the analysis using unsmoothed monthly anomalies produced highly consistent results (Supplementary Fig. 24), confirming that the inferred ENSO–soil moisture relationship is insensitive to the choice of temporal aggregation.

References:

1. Zhu, B., Tu, M. M., Wang, M. Q., et al. Soil moisture constraints override atmospheric aridity in governing vegetation dynamics on the warming Qinghai–Tibetan Plateau. Ecol. Indic. 185, 114742 (2026).

2. Zhong, S. S., Liu, Y. M., Xia, Y. N., et al. Influence of spring soil moisture in the central and western Tibetan Plateau on the thermal conditions of underlying surface. Clim. Dyn. 64, 123 (2026).

3. Rao, X. Z., Zhang, J. & Chen, T. X. Coupling of land surface thermodynamics with atmospheric dynamics effect on summer extreme precipitation in the northern Tibetan Plateau. J. Geophys. Res. Atmos. 130, e2025JD044632 (2025).

Reviewer #3:

The manuscript Warming-driven rise in soil moisture entropy signals destabilization of the Asian Water Tower presents a novel application with the use of Eigen Microstate entropy to Tibetan Plateau (TP) soil moisture. It combines observational products, entropy-based diagnostics, ENSO teleconnections, and CMIP6 projections to show the connection of that recent wetting has reduced entropy and increased hydrological organization. Moreover that future warming is projected to increase entropy and regional contrasts, potentially signaling destabilization and even the risk of regime shifts in some models. The study is methodologically rigorous and provides essential insides into telecoupling of Earth system elements across long distances, and it has the potential to make a strong contribution.

Response: We sincerely thank the reviewer for the careful reading and encouraging assessment of our work. We are especially grateful that the reviewer recognizes the novelty of applying the Eigen Microstate entropy framework to Tibetan Plateau soil moisture, the methodological rigor of our approach, and its broader value for understanding Earth system telecoupling. We address all comments point by point below and have revised the manuscript accordingly.

Overall I find the main storyline clear and compelling, but somewhat overloaded with figures and partly redundant diagnostics spread across the main text and Supplement.

Next to this, the Results section contains already substantial interpretive and discussion-like sections that should be moved to Discussion. Interestingly, (and perhaps as a result of this), the authors chose in the Discussion section not to refer to any figure or result (e.g. missing references to own findings or numbers from Results) to allow to connect their claims with own specific quantitative findings but rather refer to other studies.

So my recommendations are i) streamline the storyline by reducing the analytics to the relevant figures only, ii) move interpretations and discussions from Results to Discussion, iii) connect and refer in the Discussion with findings from this study.

Therefore, I recommend major revisions (as mainly moving existing text) for this manuscript.

Response: We thank the reviewer for these constructive and insightful comments. We fully agree with the three recommendations and have revised the manuscript accordingly.

First, we have streamlined the storyline by retaining only the figures essential to the core narrative in the main text and relocating redundant diagnostics to the Supplement materials, so that each main-text figure now carries a distinct role. Second, we have moved the interpretive and discussion-like passages out of the *Results* section, which now focuses on reporting findings, while the corresponding interpretation has been transferred to the *Discussion and Conclusion*. Third, we have fully revised the *Discussion and Conclusion* so that it explicitly connects our claims to the specific figures, results, and quantitative findings of this study, rather than relying primarily on references to other works. Detailed, point-by-point revisions implementing each of these three recommendations–including the specific figures relocated, the passages moved from *Results* to *Discussion and Conclusion*, and the added quantitative back-references–are provided in our responses to the corresponding comments below.

We believe these changes have made the manuscript more focused and the argumentation more self-contained, and we are grateful to the reviewer for prompting them.

Figures:

The current main essential main-text figures to keep (Fig. 1 – Fig 4, and Fig 5 (partly) Within Fig. 5, panels (b), (d), (e), and (f) are directly tied to the main conclusions on future entropy changes and potential regime shifts. However, the LFCA conceptual model (Fig. 5a) and the detailed eigen-mode contribution panel (Fig. 5c) are nice but not essential for the main text. I recommend moving these to the Supplement and referring to them there when introducing entropy as an early-warning metric.

Response: We thank the reviewer for this specific and constructive suggestion. We fully agree that streamlining Fig. 5 strengthens the manuscript by focusing the reader directly on the projected future entropy changes and potential regime shifts. Accordingly, we have made the following revisions:

(1) **Revised Fig. 5:** We have retained the original panels (b), (d), (e), and (f), as they provide the key evidence supporting our main conclusions. These have been re-lettered as panels (a), (b), (c), and (d) in the revised Fig. 5. The updated figure and caption are presented below.

(2) **Relocation to Supplementary Information:** As recommended, the LFCA conceptual model (formerly Fig. 5a) and the detailed eigen-mode contribution panel (formerly Fig. 5c) have been moved to the Supplementary Information. They are now presented as Supplementary Fig. 12 and Supplementary Fig. 15, respectively.

(3) **Textual Updates:** The corresponding figure references in the main text have been updated accordingly.

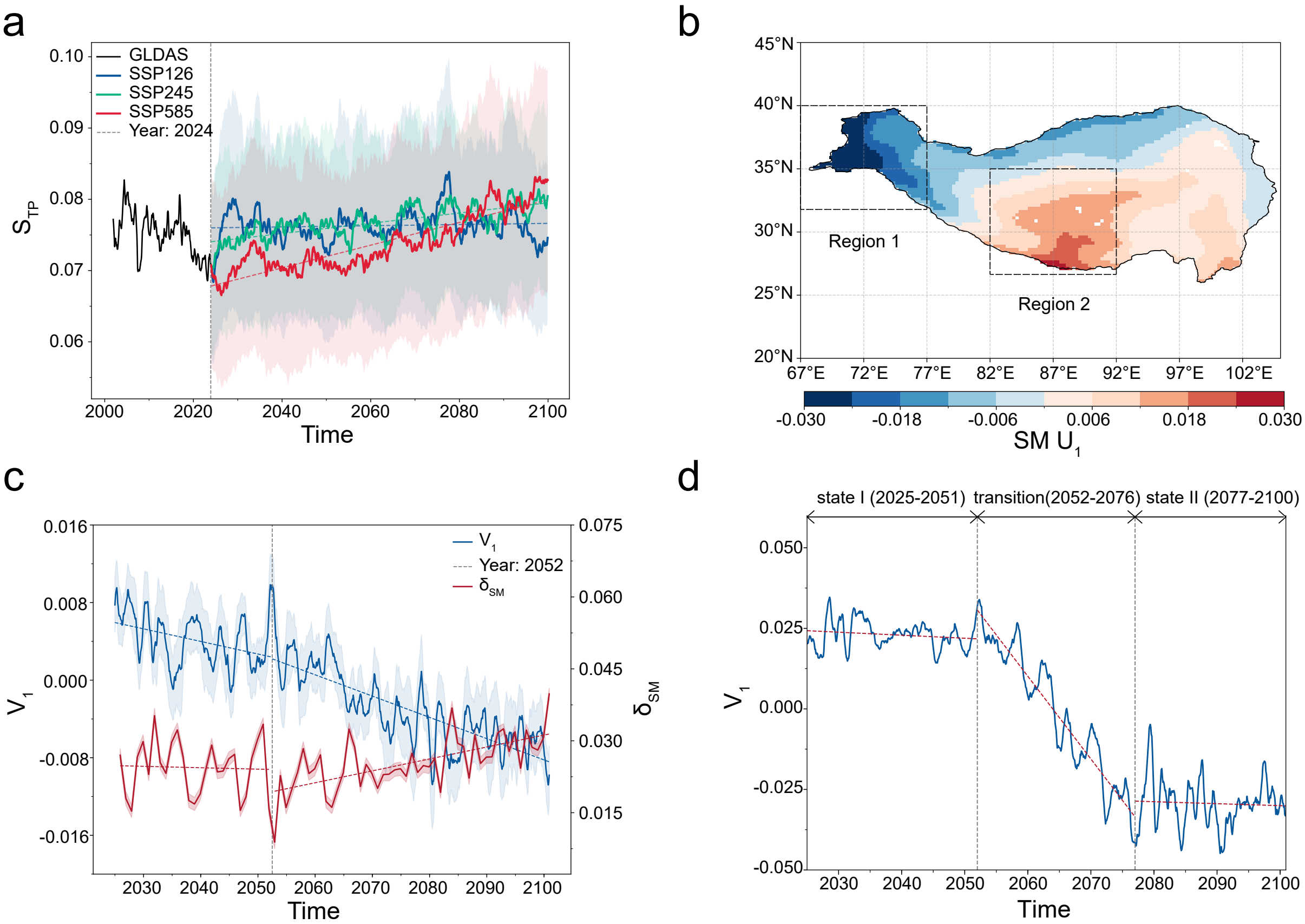

Figure 5: Projected changes in soil moisture system over the TP. (**a**) Observed (2000–2024, black) and projected (2025–2100) S_{TP} from CMIP6 models. Unlike the historical decline, future scenarios (SSP126, SSP245, SSP585) consistently exhibit significant upward trends ($p < 0.001$), suggesting a shift towards a more disordered and unstable regime. (**b**) Multi-model ensemble mean of the leading soil-moisture eigen-microstate (U_1) under SSP585, obtained by averaging the individual U_1 patterns across the 12 selected CMIP6 models. Rectangles define the key dipole centers: Region 1 (western TP, negative phase) and Region 2 (southwestern TP, positive phase). (**c**) Ensemble-mean temporal evolution of the dominant SM mode (V_1, blue) and the inter-regional SM difference (δ_{SM}, red). A critical breakpoint is identified in 2052 (grey dashed line) by segmented linear regression. V_1 exhibits a significant downward trend after the breakpoint ($p < 0.001$), with a slope exceeding twice its previous value. No significant trend is observed before the breakpoint in δ_{SM}, while a significant increase occurs thereafter ($p < 0.001$). (**d**) Representative trajectory of V_1 from the IPSL-CM6A-LR model (SSP585), detailing the three distinct stages: a relatively stable state during 2025–2051, a rapid regime shift period (2052–2076, $p < 0.001$), and the settlement into a new stable state during 2077–2100.

In the Supplement, several figures are primarily diagnostic or partially redundant with main-text plots. For example, S5–S7 (various forms of S_{TP}–ENSO correlation), S9 (Power spectral densities (PSD) of V_1), S12 (detailed monthly climatologies for the dipole regions), S19 (V_1 vs temperature in IPSL-CM6A-LR), and S21 (ONI-based ENSO event identification) could potentially be merged, simplified, or even removed without loss of the main message.

Response: We thank the reviewer for this helpful suggestion on streamlining the Supplementary

Information. Following this advice, we have carefully reviewed the SI and consolidated or removed several figures to reduce redundancy while preserving the key messages. The specific revisions are as follows:

(1) **Consolidation of S5–S7:** Former Supplementary Figs. 5–7 have been merged into a single figure (now Supplementary Fig. 6). This provides a more concise and coherent representation of the relationship between S_{TP} and ENSO. The revised figure and caption are shown below.

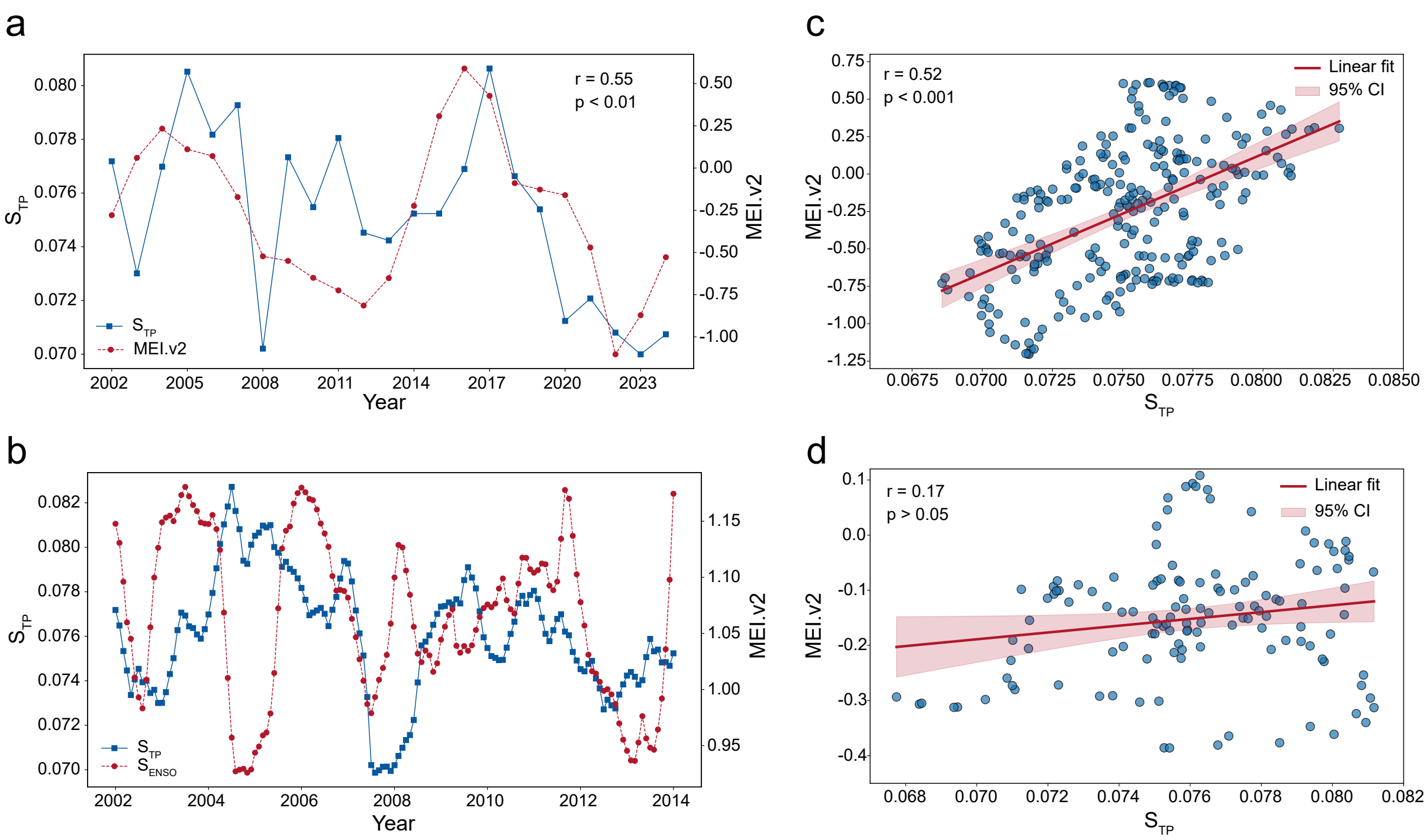

Supplementary Figure S6: Relationship between S_{TP} and ENSO. (**a**) Temporal evolution of S_{TP} (blue) and the ENSO index (MEI.v2, red). (**b**) Temporal evolution of S_{TP} (blue) and the S_{ENSO} (red) in CMIP6 historical simulations. (**c**) Scatterplot of S_{TP} versus the ENSO index (MEI.v2) during all periods. (**d**) Same as (**c**) but for neutral periods.

(2) **Simplification of S9:** Former Supplementary Fig. 9 has been simplified and renumbered as Supplementary Fig. 8. To better emphasize the interannual timescale relevant to this study, we removed the PSD of the raw V_1 time series and retained only the PSD derived from the 12-month smoothed series. The revised figure is presented below.

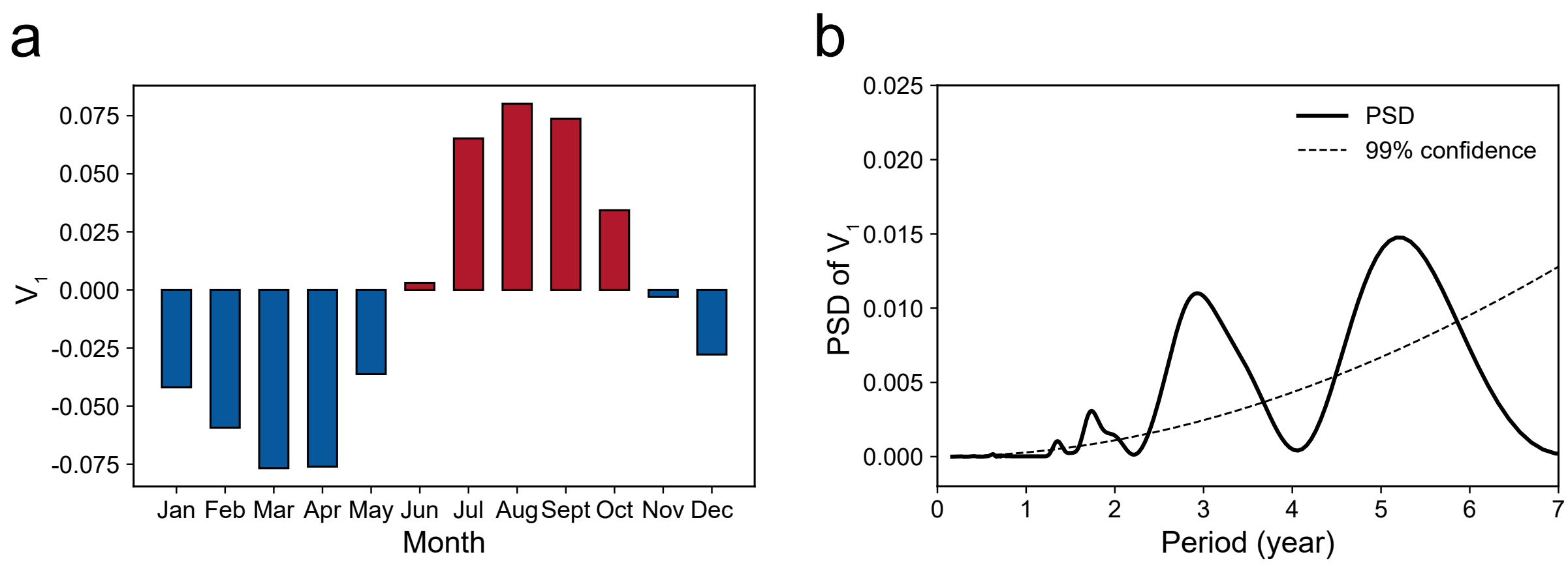

Supplementary Figure S8: Monthly climatology and power spectrum density of the temporal evolution of soil moisture EM1. (**a**) Monthly climatology of temporal evolution V_1. (**b**) Power spectral densities (PSD) of 12-month smoothed V_1 (solid line), representing interannual variability, and its 99% significance levels (dashed lines).

(3) **Removal of S12:** Former Supplementary Fig. 12 has been removed, as its essential information is already captured in the revised Supplementary Fig. 8a. We have updated the corresponding text in the *Results* section to reflect this change:

~~"*Distinct moisture regimes characterize the dipole: the western TP exhibits a significant negative phase, with SM peaking in May due to snowfall, spring melt, and low ET, whereas the southwestern TP shows a significant positive phase, with SM peaking in August under the influence of monsoonal rainfall (Supplementary Figs. 9a, 12).*~~ *Distinct moisture regimes characterize the dipole: the western TP exhibits a significant negative phase, with SM peaking in spring due to snowfall, spring melt, and low ET, whereas the southwestern TP shows a significant positive phase, with SM peaking in summer under the influence of monsoonal rainfall (Supplementary Fig. 8a).*" (Results, Line 261)

(4) **Removal of S19 and S21:** As suggested, former Supplementary Figs. 19 (V_1 vs. temperature) and 21 (ONI-based identification) have been removed, as they provided primarily diagnostic details not strictly essential to the main conclusions.

Results

The results section is generally well-structured going through observations, entropy behavior, ENSO modulation, and projections. However, here frequently the goes beyond reporting results or observations of data behavior into mechanistic speculation, broader system implications, and even discussion of socio-hydrological impacts.

While these interpretations are reasonable and justified, they would be much better placed in the Discussion. For examples

L190: attributing the SM–entropy relationship to greater hydrological buffering capacity of wetter soils and detailed ecological feedbacks.

L172 ff: proposing multi-step feedback chains (e.g., vegetation composition shifts, land–atmosphere feedbacks, cloud changes) in 2.2–2.4.

L356 ff: descriptions of cascading risks to downstream water resources and food security already hinted at in the Results.

Also the extended passages on buffering capacity, vegetation–soil feedbacks, and tipping cascades can be moved into the Discussion, where they can be integrated with the broader literature and discussed more cautiously.

Response: Thank you for this important suggestion. We agree that several parts of the original *Results* section contained interpretation and broader implications beyond the scope of a *Results* section. Accordingly, we fully revised the manuscript to keep the *Results* focused more strictly on the reported findings, while relocating these interpretive and forward-looking statements to the *Discussion and Conclusion*. Specifically:

(1) In Section 2.2 (around former L190), we have removed the relevant explanatory statements and relocated them to the *Discussion and Conclusion*, where they are used to interpret the relationship between soil moisture entropy and hydrological conditions. The revised text is as follows:

~~*"This association likely reflects the greater hydrological buffering capacity of wetter soils, which allows the system to absorb external perturbations, such as drought events or rapid increases in atmospheric evaporative demand, without large deviations in SM [57]. Enhanced buffering stabilizes the temporal evolution of soil moisture, concentrating variability into a narrower set of dominant system states and thus reducing entropy. In contrast, dry soils with limited buffering respond more abruptly to perturbations, generating larger fluctuations and a more dispersed distribution of soil moisture states, which increases entropy."*~~ (L190, in the *Results* section of the original version)

"Future projections reveal a rising trend in S_{TP} accompanied by a decline in SM, in contrast to recent decades (Fig. 5a, Supplementary Fig. 14). Declining SM implies a reduction in hydrological buffering capacity, so that the system responds more strongly to external forcing such as drought and enhanced atmospheric evaporative demand [68]. As these responses amplify, system variability is no longer concentrated in a few coherent modes but becomes increasingly distributed across many competing eigen microstates, raising the entropy S_{TP}. Because a more even distribution over eigen microstates reflects the loss of a dominant, coherent spatiotemporal pattern, this rise in disorder signals a decline in the predictability of the TP water cycle, suggesting that water-stressed conditions weaken the coherent organization that underpins the Asian Water Tower." (Discussion and Conclusion, Line 374)

(2) In Sections 2.2–2.4, we deleted the multi-step feedback descriptions involving vegetation shifts, land–atmosphere coupling, and cloud-related feedbacks from the *Results*. These elements are now synthesized in the *Discussion and Conclusion*, where they are related to the identified mid-century breakpoints in entropy changes and spatial contrast, as well as the emergence of regime-like behavior in the IPSL-CM6A-LR model, offering potential mechanistic explanations for these observed patterns. The corresponding revisions are shown below:

~~*"The abrupt SM decline after mid-century may reflect the emergence of interacting positive feedbacks that amplify drying once key thresholds are exceeded [13]. Reduced SM can weaken evapotranspiration and reduce cloud formation, enhancing short-wave radiation and shifting the surface energy balance toward sensible heating, thereby intensifying land–atmosphere warming [20]. At the same time, progressive drying may favor stress-tolerant, shallow-rooted vegetation with lower water-use efficiency and reduced*~~

~~*soil water retention, weakening the systems buffering capacity [39]. The convergence of these fast atmospheric and slower ecological adjustments could render the SM system increasingly sensitive to small climatic perturbations, potentially giving rise to threshold-like behavior consistent with the modeled transition."*~~ (L323, in the *Results* section of the original version)

"This tendency is most pronounced in the IPSL-CM6A-LR model, which undergoes a regime-shift-like transition: a rapid mid-century decline into a persistently drier state, except for relatively wetter conditions in the northeast (Fig. 5d, Supplementary Fig. 18). Such threshold behavior is consistent with the interacting positive feedbacks that amplify perturbations and progressively push the system toward a critical threshold [11]. A fast atmospheric feedback, in which reduced SM shifts the surface energy balance toward sensible heating and reinforces land–atmosphere warming [24], can combine with a slower ecological feedback, in which progressive drying favors stress-tolerant vegetation with reduced water retention [46]. The convergence of these fast and slow adjustments can progressively destabilize the SM system, providing a physical basis for the abrupt, threshold-like response captured by the entropy diagnostic and reinforcing that the projected instability risk reflects a potential regime shift rather than gradual drying alone." (Discussion and Conclusion, Line 419)

(3) We further revised the *Results* to remove the forward-looking descriptions of cascading impacts on downstream water resources and food security. These broader implications are now consolidated in the *Discussion and Conclusion*, where they are framed more cautiously in the context of continued warming-driven increases in TP soil-moisture disorder and the associated growth in spatial heterogeneity, with potential consequences for future water-resource management. The corresponding revisions are shown below:

"A broadly increasing trend in SM across the TP is reshaping regional hydrological dynamics by enhancing soil water storage, altering the partitioning of ET, and modifying runoff generation. These changes affect both the timing and magnitude of water availability across the Plateau ~~and its downstream basins~~, with important implications for the stability of the regional water cycle. Here, we quantify system disorder using eigen microstate entropy, which captures underlying shifts in structural organization." (Line 173, in the *Results* section of the original version)

~~*"In conclusion, entropy offers a system-level perspective on the non-linear, cumulative mechanisms of climate-hydrology coupling, informing climate prediction and water resource management on the TP."*~~ (Line 268, in the *Results* section of the original version)

"Such enhanced heterogeneity is expected to exacerbate uneven water availability and intensify compound extremes, with cascading impacts on major downstream river basins in South and East Asia that collectively support nearly two billion people [1, 2]." (Discussion and Conclusion, Line 403)

Discussion

As already mentioned above the Discussion broadly reflects the results but often speaks at a higher conseptual level without consistently referring back to specific figures or quantitative numbers.

For instance, statements about "new evidence for such teleconnections" and ENSO modulation of TP SM entropy should explicitly referenced to own findings such as 'the six-month lagged correlation between S_{TP} and S_{ENSO}' and the corresponding figure(s), and the spatial dipole of EM1 and its link

to δ_{SM} and S_{TP}.

Response: We thank the reviewer for pointing this out. We agree that the *Discussion and Conclusion* should more explicitly refer to specific figures and quantitative results. In the revised manuscript, we have strengthened these links to ensure that the *Discussion and Conclusion* is consistently grounded in our results, including clearer references when discussing ENSO-TP SM teleconnections and the relationship between the SM heterogeneity reflected in the spatial dipole pattern (EM1) and entropy (S_{TP}). The revised text is provided below:

"Destabilization in one component can trigger cascading responses in others, thereby amplifying systemic risks [67]. Our results provide new evidence for such coupling: SM entropy on the TP is modulated by ENSO, as evidenced by the six-month lagged correlation between S_{TP} and S_{ENSO} (Fig. 2d). We further trace the mechanism underlying this coupling: ENSO-induced precipitation anomalies imprint a west–southwest dipole on TP SM (Fig. 3a, Fig. 4a), and the strength of this inter-regional contrast governs the structural disorder of the SM system, as captured by the significant correlation between δ_{SM} and S_{TP} (Fig. 3a). The entropy framework is central to uncovering this chain." (Discussion and Conclusion, Line 354)

Likewise, when discussing future destabilization, rise in entropy, and mid-century regime shifts, the Discussion should e.g explicitly cite the 'projected upward trends in S_{TP}' (figure reference and approximate trend magnitudes). Also the identified breakpoint timing in entropy and V_1, and the model-specific trajectory of IPSL-CM6A-LR, including which spatial regions dry vs remain wetter.

So, the Discussion would greatly improve by adding targeted back-references (figure numbers and a few key quantitative values) showing a much clearer logical connection between evidence to interpretation and help a reader better to track where each statement is supported.

Response: We thank the reviewer for this suggestion. We agree that the *Discussion and Conclusion* would benefit from more explicit links to quantitative evidence. In the revised manuscript, we have added targeted back-references to figures and key numerical results when discussing future entropy increases and enhanced heterogeneity in the dipole regions, which together suggest a progressively more imbalanced and disordered system structure. We further relate these patterns to LFCA-based results, which may indicate potential signals of critical transitions. In addition, we now explicitly cite the identified breakpoints in entropy and V_1, highlighting evidence for mid-century regime shifts, and we refer to the IPSL-CM6A-LR simulations, which exhibit model-specific regime-like behavior, including distinct spatial patterns of drying and wetting. The revised text is provided below:

"Future projections reveal a rising trend in S_{TP} accompanied by a decline in SM, in contrast to recent decades (Fig. 5a, Supplementary Fig. 14). Declining SM implies a reduction in hydrological buffering capacity, so that the system responds more strongly to external forcing such as drought and enhanced atmospheric evaporative demand [68]...." (Discussion and Conclusion, Line 374)

"The projected entropy rise is accompanied by an amplification of the dipole mode marked by strengthening west–southwest contrasts, indicating increasing spatial heterogeneity within the TP hydroclimatic system (Fig. 5b, Supplementary Fig. 15). Such enhanced heterogeneity is expected to exacerbate uneven water availability and intensify compound extremes, with cascading impacts on major downstream river

basins in South and East Asia that collectively support nearly two billion people [1, 2]..." (Discussion and Conclusion, Line 401)

"The projected rise in entropy reproduces the early-warning signals of critical transitions observed in conceptual models, pointing to a heightened risk of abrupt, nonlinear shifts rather than smooth change in TP hydroclimate (Supplementary Fig. 12) [18]. Consistent with this, segmented regression identifies a breakpoint in both entropy and the dipole-mode evolution (V_1) around the mid-21st century under high-emission scenarios, after which spatial contrast intensifies sharply (Fig. 5c, Supplementary Fig. 17). This tendency is most pronounced in the IPSL-CM6A-LR model, which undergoes a regime-shift-like transition: a rapid mid-century decline into a persistently drier state, except for relatively wetter conditions in the northeast (Fig. 5d, Supplementary Fig. 18). Such threshold behavior is consistent with the interacting positive feedbacks that amplify perturbations and progressively push the system toward a critical threshold [11]...." (Discussion and Conclusion, Line 414)

Minor:

1) The authors note that originally entropy is only a measure of structural organization rather than a direct stability metric. However, much of the language ("destabilization", "early-warning signal", "critical transition") reads as if entropy is being used as a near-direct proxy for system resilience.

Response: We thank the reviewer for this careful reading, and we fully agree. We want to be explicit that our position is unchanged: eigen microstate entropy, as defined in our framework, measures the evenness of the probability distribution across dominant spatiotemporal modes. It is a measure of spatiotemporal organization, not a direct measure of resilience or stability. The reviewer has correctly identified that the unsuitability lies not in this interpretation itself but in its consistency with our wording: although we stated that entropy is a structural diagnostic rather than a stability metric, several word choices ("destabilization," "early-warning signal," "critical transition") nonetheless read as if entropy were a near-direct proxy for resilience, in tension with our own stated interpretation. We agree that this inconsistency between our position and our language was undesirable, and we have resolved it by bringing the wording throughout the manuscript, including the *title*, into line with the indirect, structural interpretation we intend.

To make explicit the indirect sense in which any remaining stability-related wording should be read, we emphasize that entropy and resilience are conceptually distinct quantities and are not equated in our analysis. Entropy characterizes how variability is distributed across spatiotemporal modes, whereas resilience concerns the system's recovery behavior following perturbations, and the two need not vary monotonically with one another. What we invoke is the empirical regularity that, across a range of high-dimensional complex systems, a build-up of structural disorder, reflected in rising spatial or structural entropy, has been observed to precede abrupt regime shifts and has been proposed as an early indicator of an approaching tipping point [1–4]. On this basis we treat rising entropy as a structural diagnostic that is consistent with, but not a measure of, reduced stability, an interpretation further supported by our LFCA model demonstration, in which rising entropy precedes the abrupt transition in a controlled setting.

Guided by this principle, we have systematically moderated the stability-related language throughout

the text so that it consistently reflects this indirect framing. Most prominently, we have revised the manuscript title to remove the direct claim of "destabilization." In the main text, (1) "destabilization" is replaced by phrases such as "increasing disorder"; (2) "early-warning signal" is reframed as "a potential indicator consistent with early-warning behavior"; (3) "critical transition" is qualified as "transition-like behavior" or "a regime-shift-like change identified in a subset of models." We have also added a clarifying statement in the *Discussion and Conclusion* that explicitly distinguishes entropy as a structural diagnostic from a direct stability metric and notes that the link to stability is indirect. The specific revised text is as follows:

~~"*Warming-driven rise in soil moisture entropy signals destabilization of the Asian Water Tower*"~~ *Warming-driven rise in soil moisture entropy signals growing instability risk in the Asian Water Tower*" (Title)

"*However, CMIP6 climate projections reveal a marked reversal: entropy increases under continued warming and regional contrasts intensify, with some models exhibiting* ~~*an abrupt mid-century transition*~~ *abrupt, transition-like behavior around mid-century. Our findings suggest that while current wetting provides a stabilizing buffer, continued warming is projected to amplify spatial heterogeneity, thereby* ~~*destabilizing in the Asian Water Tower, with significant risks for downstream water security.*~~ *elevating instability risk in the Asian Water Tower and threatening downstream water security.*" (Abstract, Line 37)

"*By quantifying the degree of disorder, entropy reveals whether variability is concentrated in a few dominant modes (order) or dispersed across many (disorder), offering a physically interpretable measure of systemic structure and* ~~*early-warning signals*~~ *a potential indicator of heightened risk of abrupt transitions [47–49].*" (Introduction, Line 117)

~~"*To evaluate soil water stability, we use eigen microstate entropy as a diagnostic of system disorder.*~~ *Here, we quantify system disorder using eigen microstate entropy, which captures underlying shifts in structural organization.*"(Results, Line 209)

"*In complex systems, rising entropy has been proposed as* ~~*an early-warning signal of reduced stability,*~~ *a potential indicator consistent with early-warning behavior linked to reduced stability and preceding abrupt transitions across diverse contexts, from theoretical models to ecological systems [47, 48].*" (Results, Line 302)

"*Across scenarios, S_{TP} reveals a pronounced upward trend with warming (Fig. 5a), indicating a progressive rise in disorder and* ~~*a potential early warning signal of stability loss*~~ *a heightened risk of reduced stability in the TP's SM system.*" (Results, Line 321)

"*This tendency is most pronounced in the IPSL-CM6A-LR model, which undergoes a regime-shift-like transition: a rapid mid-century decline into a persistently drier state, except for relatively wetter conditions in the northeast (Fig. 5d, Supplementary Fig. 18).*" (Discussion and Conclusion, Line 419)

"*First, entropy is not interpreted here as a direct measure of stability, such as the recovery rate in the CSD framework [11], but as a diagnostic of the systems degree of structural organization. Higher disorder reflects a progressive dispersion of variability across multiple competing modes, which in complex, high-dimensional systems is commonly associated with the loss of coherent organization, reduced resilience, and heightened sensitivity to perturbations [48, 56, 79, 80]. We therefore frame our results as indicating a heightened risk of nonlinear transition rather than evidence that tipping is inevitable.*" (Discussion

and Conclusion, Line 431)

"More broadly, our work highlights the value of entropy-based diagnostics ~~for detecting early-warning signals of hydrological instability~~ *for characterizing the risk of hydrological instability."* (Discussion and Conclusion, Line 477)

2) The connection to Earth system tipping elements and tipping cascades is good. However, the most compelling evidence for an actual regime-like shift is found in one CMIP6 model (IPSL-CM6A-LR), while other models show smoother changes. This should be emphasized more clearly in the Discussion and Conclusions.

Response: We thank the reviewer for this important comment. We agree that the robustness of the apparent regime-like shift needs to be more clearly qualified, particularly in terms of its dependence on individual model behavior. In response, we have revised the *Discussion and Conclusion* to explicitly clarify that the evidence for abrupt shifts is observed only in IPSL-CM6A-LR, while all other CMIP6 models show more gradual and continuous responses.The revised text is provided below:

"Finally, the clearest regime-shift signal is largely confined to a particular model (IPSL-CM6A-LR), whereas other models show more gradual responses, leaving open whether such signals represent genuine thresholds or model-specific behavior. Resolving these questions will require improved representation of cryospheric, hydrological, and land–atmosphere processes together with coordinated multi-model assessments." (Discussion and Conclusion, Line 445)

3) Moreover CMIP6 are just models of what we currently know how the Earth system is functioning but we are not sure if the same interactions will still be valid under future Earth system states. Thus, the authors should rater frame their results more carefully rather as heightened risk of nonlinear transitions and plausible early-warning signals rather than as evidence that such tipping is likely or inevitable. Explicitly acknowledging current limitations in CMIP6s representation of TP hydrology, snow and permafrost processes, and land–atmosphere coupling, and clarifying that some abrupt shifts might be model-specific artifacts

Response: We thank the reviewer for this thoughtful comment. We agree that CMIP6 simulations reflect our current understanding of Earth system processes, while the relationships and feedbacks represented in these models may not fully hold under future climate states, particularly in complex high-elevation regions such as the Tibetan Plateau.

Accordingly, we have revised the manuscript to adopt a more cautious interpretation of our results. Specifically, we now frame our findings as indicating a heightened risk of nonlinear transitions and potential early-warning signals, rather than as evidence that tipping behavior is likely or inevitable. We also explicitly acknowledge current limitations of CMIP6 in representing Tibetan Plateau hydrology, snow processes, permafrost dynamics, and land–atmosphere coupling. The revised text is provided below:

"Second, because the Asian Water Tower is defined by its cryospheric water storage, the fidelity of the future projections depends on how well these processes are represented. CMIP6 models, while grounded

in our current understanding of Earth system processes, may not fully capture system behavior under future climate states, particularly given known limitations in representing TP cryospheric dynamics and land–atmosphere coupling [71, 72]. Accordingly, while Pp emerges as the primary driver of projected entropy changes, the contributions of snow accumulation, glacier melt, permafrost thaw, and surface runoff to entropy dynamics remain insufficiently assessed and constitute an important source of uncertainty. " (Discussion and Conclusion, Line 437)

4) Avoid oscillating within a subsection between present and future, or between ENSO and non-ENSO drivers; this will make each block easier to follow.

Response: We thank the reviewer for this structural observation. Upon careful re-reading, we agree that Sections 2.2 and 2.4 in the Results, as well as parts of the *Discussion and Conclusion*, contained oscillations between different temporal frames and between ENSO and non-ENSO drivers that may impede readability. In the revised manuscript, we have reorganized these sections as follows:

In Section 2.2, we now present the long-term entropy decline driven by wetting as a self-contained block first, covering the overall trend, the acceleration after 2018, and the spatial relationship between SM and entropy. Only after this block is complete do we introduce ENSO modulation as a distinct second block, with a clear transitional sentence separating the two. This ensures that the non-ENSO long-term signal and the ENSO-driven interannual variability are each presented as coherent, uninterrupted narratives.

In Section 2.4, we focuse exclusively on CMIP6 projections without returning to historical data, eliminating the present-future oscillation identified by the reviewer. The original historical statement (e.g., " ~~Historically, the TP's soil moisture was governed by a single dominant mode of uniform wetting, characterized by a highly concentrated p and low total entropy.~~") has been removed from this section.

In the *Discussion and Conclusion*, we have also reorganized the structure to ensure that each paragraph maintains a consistent thematic focus, avoiding overlap between ENSO and non-ENSO drivers, as well as between present-day observations and future projections. Specifically, the first paragraph is devoted to the broader significance and underlying mechanisms of the ENSO–TP teleconnection revealed by the entropy metric. The second, third, and fourth paragraphs shift their focus exclusively to future changes. Specifically, they elucidate the potential mechanisms underlying the negative relationship between systemic disorder and water availability, highlight the amplification of regional heterogeneity, and explore the possible positive feedback mechanisms and broader implications associated with the regime-shift-like change identified in a subset of models. The fifth and sixth paragraphs discuss the inherent constraints of the current framework, emphasizing that the application of entropy metrics to evaluate hydroclimatic system stability requires cautious interpretation, while also noting the data limitations associated with in situ soil moisture observations, assimilation products, and CMIP6 simulations. Finally, the concluding paragraph highlights the overarching significance of this research, emphasizing its novel insights into hydrological stability and its implications for future climate adaptation.

5) Some descriptive passages closely restate what is already visually obvious from multiple panels

(e.g. repeated explanations of El Niño increasing spatial contrasts and La Niña reducing them). Trimming these repeated descriptions will free space for more concise, evidence-based linking of results and discussion.

Response: We thank the reviewer for this helpful suggestion. We have revised the *Results* section to reduce repetitive descriptive passages and improve conciseness. In particular, we removed redundant explanations of ENSO-induced spatial patterns and instead focus on presenting key quantitative relationships. The revised text retains the essential directional information (El Niño vs. La Niña) in a more concise form. The corresponding text has been revised as follows:

"A series of connection analyses support this mechanism. As shown in Fig. 3c, S_{TP} correlates strongly with the SM difference between the positive- and negative-phase regions of U_1 ($r = 0.66$, $p < 0.001$) ~~, demonstrating that greater spatial contrasts correspond to higher entropy, whereas more coherent conditions correspond to lower entropy. We confirm that ENSO intensity is positively associated with this inter-regional difference ($r = 0.65$, $p < 0.001$; Supplementary Fig. 11a): El Niño events amplify regional disparities, while La Niña suppresses them. This spatial asymmetry is further illustrated in Fig. 3d, where El Niño years typically feature positive SM anomalies between the dipole regions–wetter in the negative-phase region and drier in the positive-phase region. By contrast, La Niña years show the opposite pattern, while neutral years exhibit minimal contrasts. The monthly inter-regional difference also closely tracks the ENSO index (Supplementary Fig. 11b, $r = 0.37$, $p < 0.001$), underscoring the robust linkage between ENSO variability and regional hydroclimatic contrasts.~~ *This spatial asymmetry is modulated by ENSO intensity ($r = 0.65$, $p < 0.001$; Supplementary Fig. 10a), with El Niño (La Niña) corresponding to enhanced (reduced) contrasts and weak contrasts during neutral conditions (Fig. 3d). This linkage is further supported by the significant monthly tracking between the ENSO index and inter-regional differences ($r = 0.37$, $p < 0.001$; Supplementary Fig. 10b)."* (Results, Line 253)

6) Add information on the chosen length and step of moving windows used for entropy and correlation calculations and number of modes retained or focused on in figures (e.g. rationale for limiting to the first four eigen microstates).

Response: We thank the reviewer for this helpful suggestion. We have fully revised the manuscript to clarify the moving-window settings for entropy and correlation analyses (*Methods* and Supplementary Note S3), and the rationale for focusing on the first four eigen microstates in the figures. The point-by-point revisions are organized below.

(1) Moving-window settings for entropy and correlation analyses:

In this study, the three moving-window settings were not chosen independently but follow a single principle: for each variable we use the shortest window that still yields a stable estimate, keeping the analysis close to the characteristic timescale of the process being resolved. Because soil moisture and temperature are sampled at different resolutions (monthly versus daily) and their target signals operate on different timescales, this common criterion leads to a 3-year window for S_{TP}, a 1-year window for S_{ENSO}, and a 2-month aggregation for the correlation analysis. Specifically, a 3-year moving window with a 1-month step was used for soil moisture entropy (S_{TP}), providing sufficient

samples for stable entropy estimation while preserving the characteristic interannual variability associated with ENSO-related soil moisture dynamics. A 1-year moving window with the same monthly step was used for ENSO entropy (S_{ENSO}), allowing individual ENSO events to be resolved without blending successive opposite-phase events. For the correlation analysis between soil moisture and ENSO, a 2-month temporal aggregation was applied to the monthly soil moisture anomalies to match the widely used bi-monthly definition of the MEI.v2 index. These methodological details have now been added to the *Methods* section. In addition, to directly address the reviewer's comment, we have added a new dedicated section (Supplementary Note S3) that provides the full rationale for each window selection together with supporting sensitivity analyses.

The corresponding revisions are as follows.

"To quantify the temporal evolution of entropy, moving-window analyses were performed using variable-specific parameter settings. For soil moisture entropy (S_{TP}), a 3-year moving window with a 1-month step was adopted. This window provides sufficient samples for robust entropy estimation while preserving the characteristic interannual variability associated with ENSO-related soil moisture dynamics. For ENSO entropy (S_{ENSO}), a 1-year moving window with the same monthly step was used to resolve individual ENSO events while avoiding signal cancellation caused by blending successive opposite-phase ENSO events. The rationale for these window selections and the associated sensitivity analyses are provided in Supplementary Note S3." (Methods, Line 601)

Supplementary Note S3, Page 29:

Supplementary Note S3 Rationale for moving-window selection

The window lengths for each entropy or correlation analysis were not selected independently but followed a common criterion: for each variable, we chose the shortest window that provides sufficient samples to ensure stable estimates while remaining as close as possible to the characteristic timescale of the process being resolved. The resulting differences across variables arise for two reasons: (i) the variables have different temporal resolutions (monthly soil moisture versus daily temperature), and (ii) the variables represent processes operating on different timescales, ranging from ENSO-related interannual variability to the bi-monthly MEI.v2 index. Sensitivity analyses further confirm that the main conclusions are insensitive to reasonable variations in window length.

S3.1 Three-year window for S_{TP}

The 3-year moving window used to calculate soil moisture entropy (S_{TP}) was selected based on both statistical and physical considerations. From a statistical perspective, because S_{TP} is derived from monthly data, a 3-year window provides a sufficient sample size for robust estimation of the underlying spatiotemporal dependence structure. And substantially shorter windows may introduce sampling instability into the entropy estimate.

From a physical perspective, a 3-year window is broadly consistent with the characteristic timescale of ENSO variability (typically 2–7 years), allowing the associated interannual soil moisture signal to be resolved without blending multiple ENSO events within a longer averaging window. This choice is further supported by the temporal evolution of the ENSO-related soil moisture dipole mode (V_1), which was identified from the full study-period dataset and exhibits dominant periodicities of approximately 2–3.8 years.

To further evaluate the sensitivity of this choice, we repeated the analysis using alternative window lengths of 2 and 4 years. The resulting S_{TP} time series remained highly consistent with the original 3-year estimate, with Pearson correlation coefficients exceeding 0.7 in both cases (Supplementary Fig. 23a). In addition, the maximum lag correlations between S_{TP} and S_{ENSO} remained above 0.5 for both alternative windows (Supplementary Fig. 23b), confirming that the main conclusions are robust to the choice of window length. However, the 2-year window yields a more fluctuating S_{TP} time series, whereas the 4-year window produces a noticeably smoother evolution. Therefore, the 3-year window provides a balanced compromise between preserving temporal variability and reducing estimation noise.

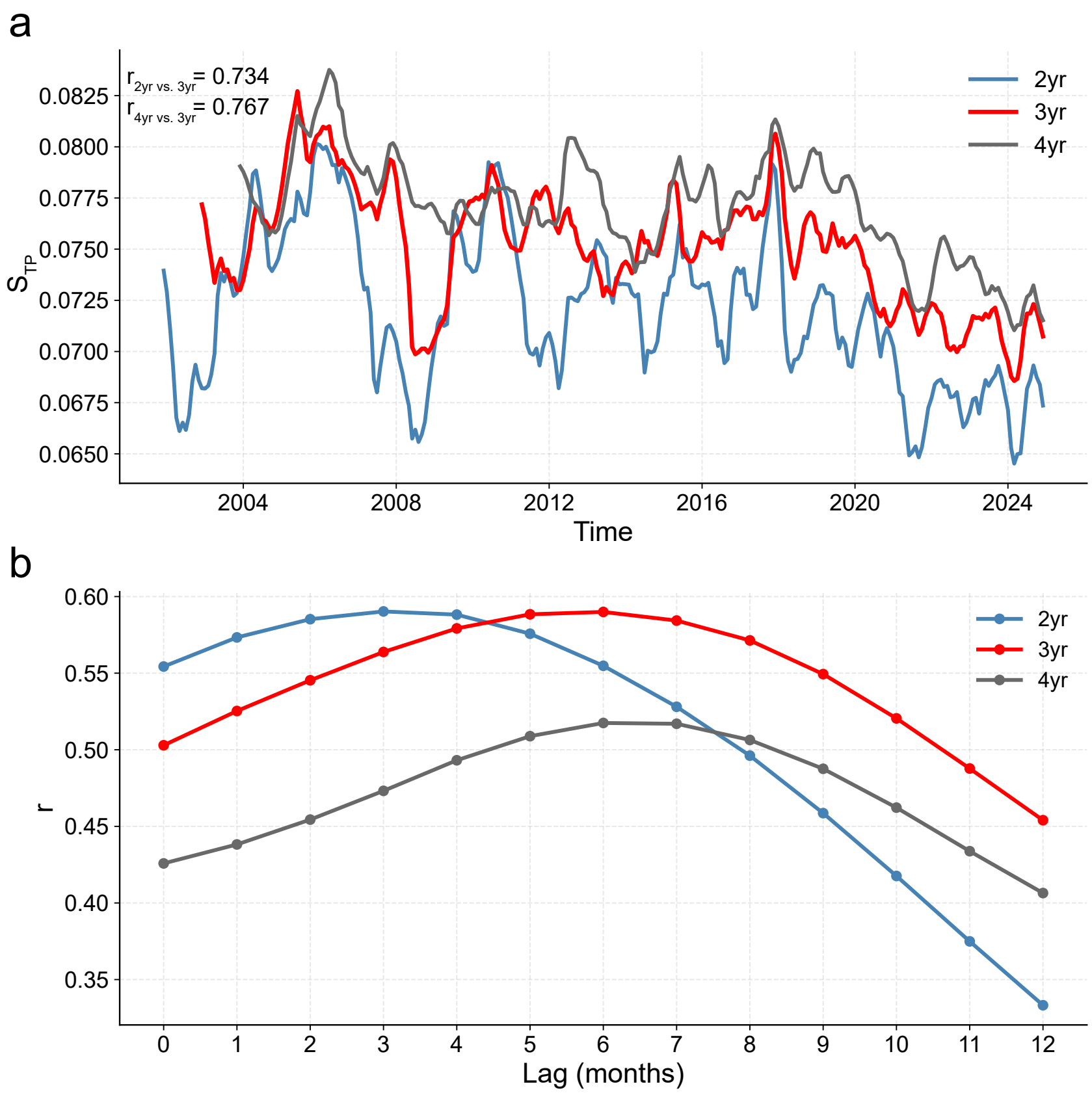

Supplementary Figure S23: Sensitivity analysis of moving-window length for S_{TP} estimation. (**a**) Comparison of S_{TP} derived using 2-, 3-, and 4-year moving windows, showing high consistency with the 3-year estimate. (**b**) Lag correlations between S_{TP} and S_{ENSO} across window lengths, confirming a robust lead–lag relationship.

S3.2 One-year window for S_{ENSO}

The 1-year moving window used to calculate ENSO entropy (S_{ENSO}) was adopted because the daily temporal resolution of the underlying temperature fields provides sufficient sampling within each annual window ($N = 365$) for stable entropy estimation. A substantially longer window would increase the likelihood of averaging across successive opposite-phase ENSO events (e.g., the 1997 El Niño followed by the 1998 La Niña), potentially causing signal cancellation and obscuring the temporal evolution of ENSO complexity. This choice is also consistent with previous studies that have used annual windows to characterize the temporal evolution of ENSO variability [4, 5].

S3.3 Two-month window for correlation analysis

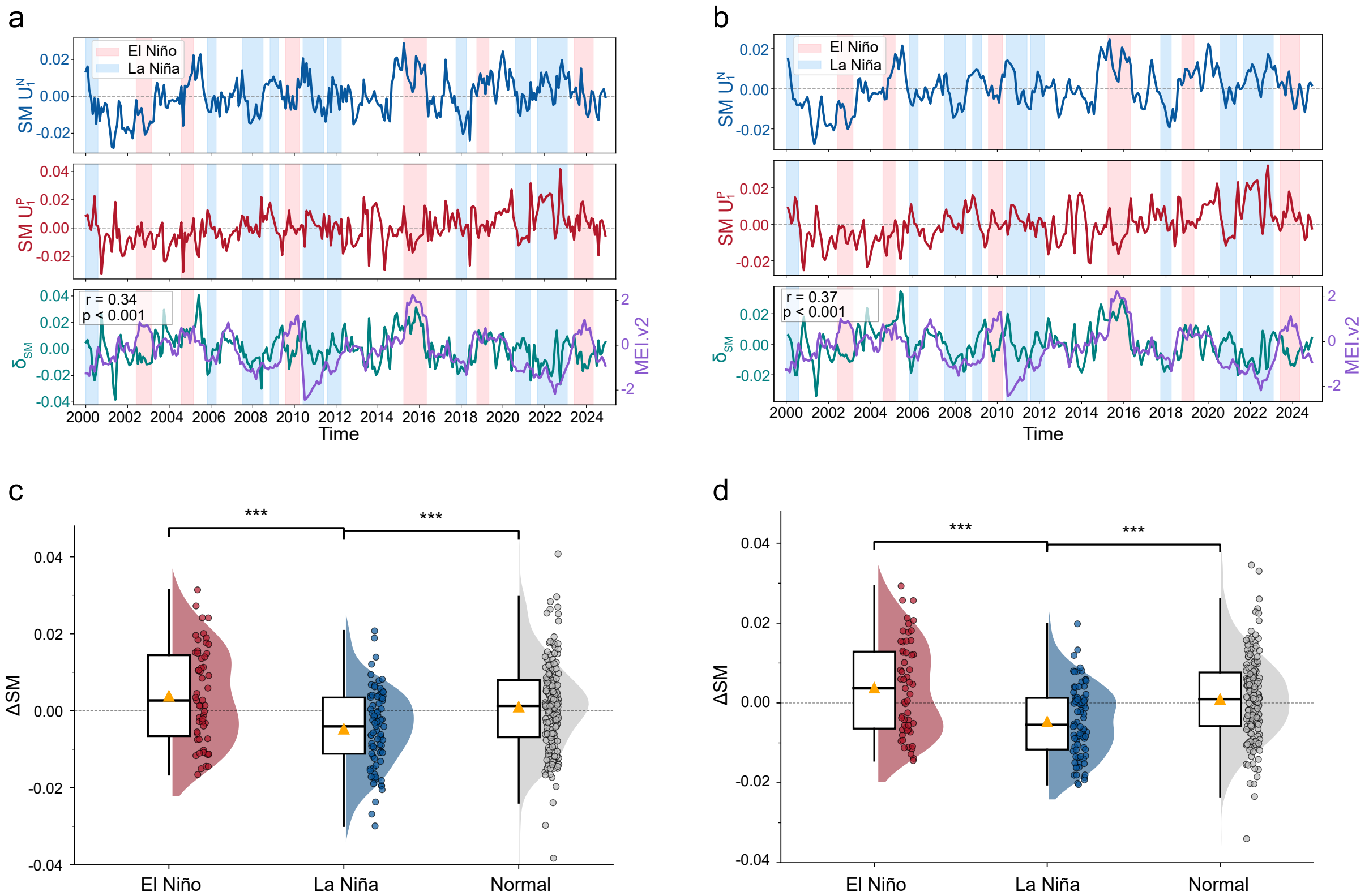

Supplementary Figure S24: Insensitivity of the relationship between SM spatial heterogeneity and ENSO to smoothing window. (**a**) and (**b**) show the SM anomalies in the negative and positive U_1 phase regions (SM U_1^N and SM U_1^P, respectively), together with their inter-regional difference (δ_{SM}) and the MEI.v2 index. Panel (**b**) applies a 2-month running mean to SM-related variables, while panel (**a**) shows monthly values. The correlations between δ_{SM} and MEI.v2 are 0.34 and 0.37, respectively ($p < 0.001$), indicating weak sensitivity to smoothing. (**c**) and (**d**) show violin plots of δ_{SM} based on monthly values and a 2-month running mean, respectively, during El Niño (red), La Niña (blue), and neutral (gray) periods. Boxplots show the median, interquartile range (IQR), whiskers within 1.5×IQR, and mean values (yellow triangles). Significant differences (***, $p < 0.001$) were identified between El Niño and La Niña, and between La Niña and neutral, based on one-way ANOVA and two-sided Mann–Whitney U-test. Both cases consistently show enhanced (reduced) δ_{SM} during El Niño (La Niña), with weak contrasts under neutral conditions.

In Fig. 3 and Supplementary Fig. 10, a 2-month moving average was used to analyze monthly soil moisture anomalies in the positive and negative U_1 phase regions (SM U_1^P and SM U_1^N), as well as their inter-regional difference (δ_{SM}). This was done to match the bi-monthly definition of the MEI.v2 index, enabling a more direct comparison between soil moisture variability and ENSO intensity.

Repeating the analysis using unsmoothed monthly anomalies produced highly consistent results (Supplementary Fig. 24), confirming that the inferred ENSO–soil moisture relationship is insensitive to the choice of temporal aggregation.

(2) Rationale for focusing on the first four eigen microstates:

In Supplementary Fig. 15, we show the temporal evolution of the mean contributions of the four dominant eigen microstates to S_{TP} under the SSP126, SSP245, and SSP585 scenarios. These four leading modes were selected because they collectively explain more than 70% of the total variability

of entropy in each scenario, indicating that they capture the dominant features of soil moisture entropy evolution.

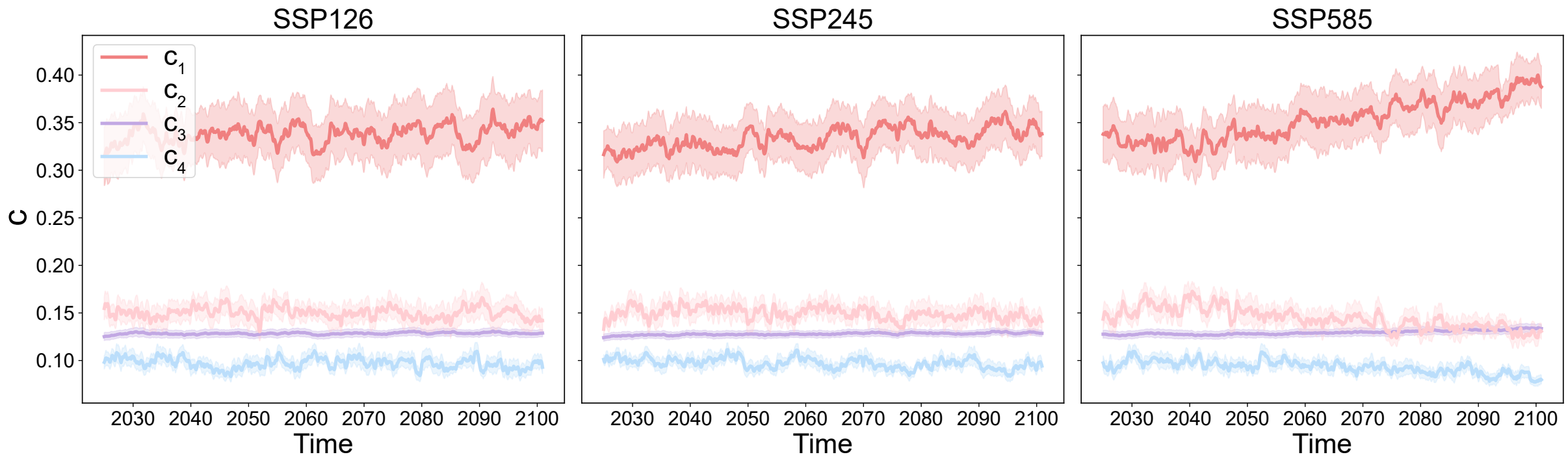

Supplementary Figure S15: Temporal evolution of mean contributions of the four dominant eigen microstates to S_{TP} under SSP126, SSP245, and SSP585 scenarios, collectively explaining over 70% of the variability in S_{TP} under each scenario.

7) Given the fact that there are already too many figures the authors may consider to potentially add a conceptual figure on diagnostic steps. Add one small conceptual workflow panel, as a panel in Fig. 1 (e.g. Fig. 1e), or in the Methods as Fig. M1 (if the journal allows), or in SI if space is tight. A compact schematic of the workflow from datasets EMT/entropy ENSO link dipole mechanism CMIP6 projections). This could help non-specialist readers follow a fairly technical pipeline and Clarify where each method/figure fits. Keep it very simple: boxes and arrows, not detailed equations.

Response: We thank the reviewer for this constructive suggestion. We fully agree that a conceptual workflow panel would significantly improve the clarity of the manuscript. In the revised manuscript, we have added a compact schematic of the research workflow to Fig. 1 in main text (Fig. 1e). This panel summarizes the diagnostic steps, from dataset selection and entropy-based analysis to mechanism attribution and future projections. To further assist readers in navigating the technical pipeline, we have explicitly annotated each step with its corresponding figure or *Methods* section, as reviewer suggested. The updated Fig. 1 is provided below.

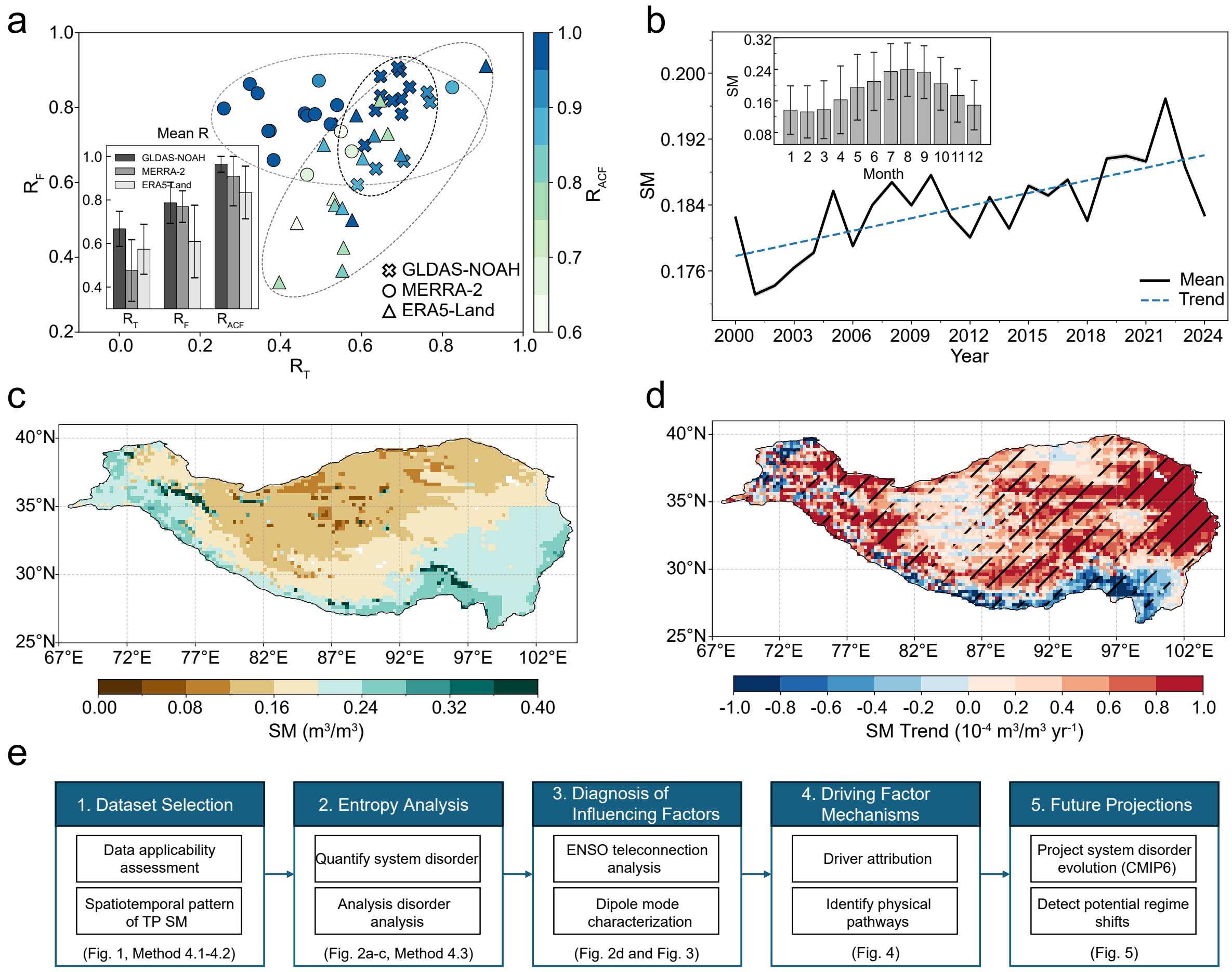

Figure 1: Dataset evaluation, spatiotemporal characteristics, and analytical framework of SM over the TP. (**a**) Temporal and spectral correlations (R_T and R_F), with colors indicating the correlation between autocorrelation sequences (R_{ACF}), computed between in-situ observations and corresponding SM product estimates. The inset bar chart summarizes the three evaluation metrics, with error bars indicating the standard deviation. (**b**) Temporal evolution of annual mean SM from 2000 to 2024, with shading indicating the standard error. The blue dashed line represents a significant upward trend ($p < 0.001$). The inset bar chart shows the climatological monthly mean SM, with error bars denoting the standard deviation. (**c**) Spatial distribution of mean SM. (**d**) Spatial patterns of SM trends from 2000 to 2024. Regions with $p < 0.05$ are marked with diagonal lines. (**e**) Conceptual schematic of the research workflow.

References:

1. Goltz, C., & Böse, M. Configurational entropy of critical earthquake populations. Geophys. Res. Lett. 29, 51–1 (2002).

2. Tirabassi, G. & Masoller, C. Entropy-based early detection of critical transitions in spatial vegetation fields. Proc. Natl. Acad. Sci. 120, e2215667120 (2023).

3. Liu, T., Niu, X. Z., Zhang, M. L., et al. Phase transition revealed by eigen microstate entropy. arXiv preprint arXiv:2512.23086 (2025) https://doi.org/10.48550/arXiv.2512.23086.

4. Xu, L., Patterson, D., & Levin, S. A., et al. Non-equilibrium early-warning signals for critical transitions in ecological systems. Proc. Natl. Acad. Sci. 120, e2218663120 (2023).

Point-by-point response to Reviewers' comments

In the following, the referee comments will be shown in blue, our responses in black, original manuscript content in *black italics*, and the quoted changes we made to the manuscript main text in *green italics*.

Reviewer #1:

I greatly appreciate the authors' detailed response, which has effectively addressed my previous questions. In consideration of the uncertainties also mentioned by the other reviewers, I have carefully examined the site comparisons in the attached Figure FS2 and found that the error, or discrepancy, at the Naqu site is considerably large. I would kindly ask the authors to explain this phenomenon, specifically regarding whether it results from inherent errors at the monitoring station itself or from uncertainties induced by the particular conditions at this site.

Response: We thank the Reviewer for this careful examination. We agree that the Naqu monitoring network shows relatively larger discrepancies among the four networks, and we have looked into this in detail. Our assessment is that the discrepancy likely results from site-specific environmental conditions and the limited observational coverage.

The Naqu network lies at ∼4500 m in a cold semi-arid alpine climate with pronounced seasonal freeze–thaw cycles [1, 2]. Dielectric probes respond only to liquid water, so observed values drop sharply during the frozen season, whereas the products use different parameterizations of frozen-soil hydrology that may not fully represent liquid-ice partitioning, unfrozen water dynamics, and freeze-thaw transitions, thereby introducing systematic cold-season disagreement [3, 4]. In addition, the 5 cm point measurements are compared against layer-averaged products (0-5 cm for MERRA-2, 0-7 cm for ERA5-Land, 0-10 cm for GLDAS-NOAH), and this depth mismatch is amplified where vertical moisture gradients are strong.

In addition, limited long-term in situ observations may have contributed to this discrepancy. Among the 11 Naqu monitoring sites, only two sites (Naqu and MS) have records longer than six years, while the remainder are shorter than four years [1, 2]. Coverage is sparsest during 2015–2016, which coincides with the largest deviations, and a data gap from September 2017 to July 2018 leaves a one-year interval between adjacent observations (August 2017 and August 2018), amplifying the apparent fluctuation in the plotted series.

Nevertheless, we emphasis that the in-situ comparison enters our analysis at a single point: the selection of the soil moisture product. All entropy diagnostics are computed entirely from gridded fields and use no station data. The product ranking is unchanged whether Naqu is included or excluded, with GLDAS-NOAH performing best overall in both cases.

We have added the above analysis to the *Results* section of the revised manuscript as follows:

"We find that GLDAS-NOAH consistently outperforms the other datasets across all evaluation criteria. Specifically, it exhibits the highest R_T with in situ observations and the lowest variability, followed by ERA5-Land, while MERRA-2 performs the worst (Fig. 1a). This superior performance is broadly consistent across the four examined subregions, demonstrating its stable temporal fidelity at finer spatial

scales (Supplementary Fig. 2). However, all three products show their largest discrepancies at the Naqu subregion. These deviations may reflect the high-altitude cold alpine environment with pronounced seasonal freeze–thaw cycles, together with the short and discontinuous in situ records available there [62, 63].” (Results, Line 164)

References:

1. Zhang, P., Zheng, D. H., van der Velde, R., et al. Status of the Tibetan Plateau observatory (Tibet-Obs) and a 10-year (2009–2019) surface soil moisture dataset. Earth Syst. Sci. Data 13, 3075–3102 (2021).

2. Zhang, P., Zheng, D. H., van der Velde, R., et al. A dataset of 10-year regional-scale soil moisture and soil temperature measurements at multiple depths on the Tibetan Plateau. Earth Syst. Sci. Data 14, 5513–5542 (2022).

3. Deng, M. S., Meng, X. H., Lu, Y. Q., et al. Impact and sensitivity analysis of soil water and heat transfer parameterizations in community land surface model on the Tibetan Plateau. J. Adv. Model. Earth Syst. 13, e2021MS002670 (2021).

4. Letcher, T., Eylander,J., Shoop, S., et al. Are the Noah and Noah-MP land surface models accurate for frozen soil conditions? Cold Reg. Sci. Technol. 220, 104149 (2024).

Reviewer #2:

Thank you for your thorough and thoughtful revisions. All my concerns have been fully addressed. Great work!

Response: We thank the Reviewer warmly for the positive assessment and for the constructive comments throughout the review process, which have substantially improved the manuscript.

Reviewer #3:

The authors have addressed the major issues by streamlining the manuscript's storyline and structure, including merging figures where necessary, moving the interpretations from the 'Results' section to the 'Discussion' section, and changing the wording to a more appropriate style with more careful phrasing. They have also provided more rigorous references for the discussion points in the figures and tables.

Response: We thank the Reviewer for the positive assessment and for the guidance that shaped these revisions.
